# Evolution and origin of vomeronasal-type odorant receptor gene repertoire in fishes

**DOI:** 10.1186/1471-2148-6-76

**Published:** 2006-10-03

**Authors:** Yasuyuki Hashiguchi, Mutsumi Nishida

**Affiliations:** 1 Division of Molecular Marine Biology, Ocean Research Institute, University of Tokyo, Tokyo, Japan

## Abstract

**Background:**

In teleost fishes that lack a vomeronasal organ, both main odorant receptors (ORs) and vomeronasal receptors family 2 (V2Rs) are expressed in the olfactory epithelium, and used for perception of water-soluble chemicals. In zebrafish, it is known that both ORs and V2Rs formed multigene families of about a hundred copies. Whereas the contribution of V2Rs in zebrafish to olfaction has been found to be substantially large, the composition and structure of the V2R gene family in other fishes are poorly known, compared with the OR gene family.

**Results:**

To understand the evolutionary dynamics of V2R genes in fishes, V2R sequences in zebrafish, medaka, fugu, and spotted green pufferfish were identified from their draft genome sequences. There were remarkable differences in the number of intact V2R genes in different species. Most V2R genes in these fishes were tightly clustered in one or two specific chromosomal regions. Phylogenetic analysis revealed that the fish V2R family could be subdivided into 16 subfamilies that had diverged before the separation of the four fishes. Genes in two subfamilies in zebrafish and another subfamily in medaka increased in their number independently, suggesting species-specific evolution in olfaction. Interestingly, the arrangements of V2R genes in the gene clusters were highly conserved among species in the subfamily level. A genomic region of tetrapods corresponding to the region in fishes that contains the V2R cluster was found to have no V2R gene in any species.

**Conclusion:**

Our results have indicated that the evolutionary dynamics of fish V2Rs are characterized by rapid gene turnover and lineage-specific phylogenetic clustering. In addition, the present phylogenetic and comparative genome analyses have shown that the fish V2Rs have expanded after the divergence between teleost and tetrapod lineages. The present identification of the entire V2R repertoire in fishes would provide useful foundation to the future functional and evolutionary studies of fish V2R gene family.

## Background

Olfaction is a sense for recognizing environmental chemicals. In many animals, olfaction plays crucial roles in various activities, such as foraging, migration, and mating. In vertebrates, odor chemicals are perceived by three evolutionary distinct groups of seven-transmembrane G protein-coupled receptors (GPCRs). Genes encoding the main odorant receptors (ORs) form the largest multigene family in vertebrates. For example, over 1,000 distinct OR copies have been identified in the mouse genome [1,2]. In addition to ORs, vertebrates have two distinct families of GPCRs for chemical receptors, called vomeronasal receptors family 1 (V1Rs) and family 2 (V2Rs). In mammals, these receptors are mainly expressed in the vomeronasal organ [3-6], and are considered to be used for detecting pheromones. The V1R gene repertoire has been described in several mammalian species, the numbers of intact genes varying from a few to over 150 [7,8] among species. The repertoire of V2R genes has been described in mice and rats [9]. The number of intact V2R genes is counted 61 in mice and 57 in rats [9].

In teleost fishes that lack a vomeronasal organ, on the other hand, both main odorant and vomeronasal receptors are expressed in the olfactory epithelium [10-12]. Recent database studies have revealed that fish ORs and V2Rs form multigene families with a hundred copies, respectively [13-15]. In addition, one V1R homolog has also been found in several fishes, and its expression confirmed in the olfactory epithelium [12].

In the previous study, we identified 88 V2R genes and pseudogenes in the zebrafish genome [15]. This number is not small compared with the number of OR sequences in this species (133 copies) [13]. Thus, in zebrafish, the contribution of V2R to olfaction seems substantially large. The repertoire of V2R genes in each fish is considered to reflect the ability of olfaction in fish species, given that different V2Rs bind to different sets of odor chemicals. The repertoire of V2R genes in fishes, however, is almost unknown except in zebrafish [15] and fugu [16,17].

It has been indicated that fish V2Rs recognize mainly amino acids [18-20]. Consequently, they are considered to be receptors for naturally occurring odors, not pheromones, because, in fishes, amino acids are common odorant substances found in natural waters [21]. However, recent studies on mammals have indicated that some V2Rs recognize peptides released by individuals and are used for chemical communication. For example, a peptide pheromone secreted from the extraorbital lacrimal gland of male mice (the peptide pheromone was contained in the tears of male mice) was suggested to be recognized by V2Rs [22]. V2Rs may also be used as receptors for small peptides that serve as ligands for major histocompatibility complex (MHC) molecules [23]. MHC-based sexual selection is also known to involve olfactory mechanisms in fishes [24,25]. For instance, female three-spined stickleback *Gasterosteus aculeatus *has been suggested to assess the degree of MHC diversity of their potential partners by sensing peptides for MHC ligands [26]. V2Rs in fish are possibly also involved in chemical communication by small peptides like MHC ligands as in mammals. Therefore, understanding the evolutionary dynamics of fish V2Rs may provide some insights into the mechanisms of sexual selection and speciation in fishes.

**Figure 1:**
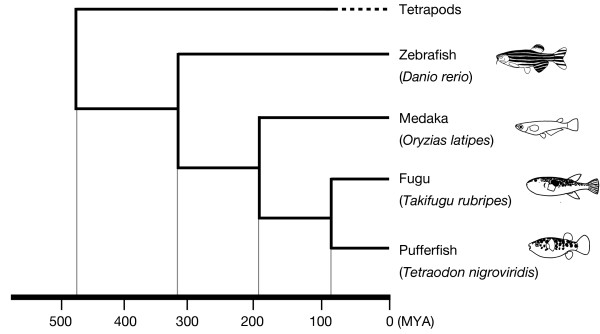
Phylogenetic relationship and estimated divergence times [25] of zebrafish, medaka, fugu, and pufferfish.

The purpose of this study is to understand the evolutionary dynamics of the fish V2R gene family. For this purpose, we identified V2R sequences in zebrafish *Danio rerio*, medaka *Oryzias latipes*, fugu *Takifugu rubripes*, and spotted green pufferfish *Tetraodon nigroviridis *from their draft genome sequences by using several computational methods. Recent advances in the molecular phylogenetic study of fishes have enabled us to infer the evolutionary process of fish V2Rs with relatively reliable divergence time estimation (Figure 1) [27]. In this study, the comparison and phylogenetic analysis of V2R repertoires in these fishes implied the organization and evolution of the fish V2R gene family. In addition, a possible evolutionary origin of the fish V2R family was inferred by comparing the genomic regions in fishes containing V2R genes with the homologous regions in some tetrapod species.

## Results

### Repertoires of V2R genes in zebrafish, medaka, fugu, and pufferfish

**Figure 2:**
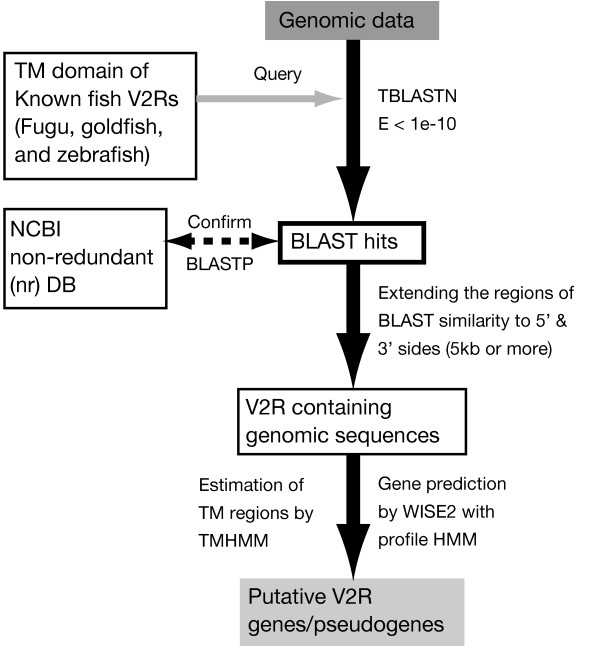
A flow chart of the procedure of V2R gene identification from draft genome sequences.

In order to characterize V2R genes in teleost fishes, we searched zebrafish, medaka, fugu, and pufferfish genomic sequences using the trans-membrane (TM) domain of several representative fish V2Rs known in fugu [16], goldfish [10], and zebrafish [15] as queries. In this study, we identified V2R genes by combining a low-threshold TBLASTN search with profile hidden Markov model- (HMM) based gene prediction. The identification procedure of V2R genes/pseudogenes is shown as a flow chart in Figure 2. Putative fish V2R sequences obtained by this search were classified into two classes, putatively functional genes and nonfunctional pseudogenes. In this study, a gene was considered putatively functional if it had a complete coding sequence with up to two disruptions. If a gene had more than two disruptions or lacked the regions from TM1 to TM7, it was considered as a pseudogene. We confirmed the exon/intron junctions by looking at the alignments of predicted V2R sequences, because V2R genes contain multiple exons. Almost all exon/intron boundaries in our predicted genes were demarcated by standard GT/AG splice sites. We identified several V2R sequences with the complete TM regions but with short N-terminal region. Particularly in fugu and medaka, these sequences were found mainly in the scaffolds less than 3 kb. These short sequences appear to be attributed to incomplete genomic data in these fishes. In the present study, V2R sequences less than 2,400 bp were considered to be "partial sequences", and were not used in the phylogenetic analysis to avoid the loss of information. These partial sequences are indicated in Supplementary tables [see Additional files 3, 4, 5, 6]. These partial sequences were also counted as putatively functional genes unless they have more than three disruptions.

As a result of the database search and gene prediction, we identified 57, 39, 38, and 23 V2R sequences in zebrafish, medaka, fugu, and pufferfish genomes, respectively. In these V2R sequences, 46 zebrafish, 22 medaka, 30 fugu, and 12 pufferfish genes were considered to be putatively functional. The proportion of putatively functional members in the V2R sequences in zebrafish (82%) and fugu (78%) were higher than in medaka (56%) and pufferfish (52%). However, these percentages may not be reliable because some of the V2R partial sequences may be attributed to the redundancy of the draft genome sequences. Indeed, several putative V2R partial sequences identified in the previous version of the zebrafish genome assembly (Zv. 4) [15] turned out to be redundant in the latest version of the genome assembly (Zv. 5) examined in this study.

### Phylogenetic relationships of fish V2R genes

**Figure 3:**
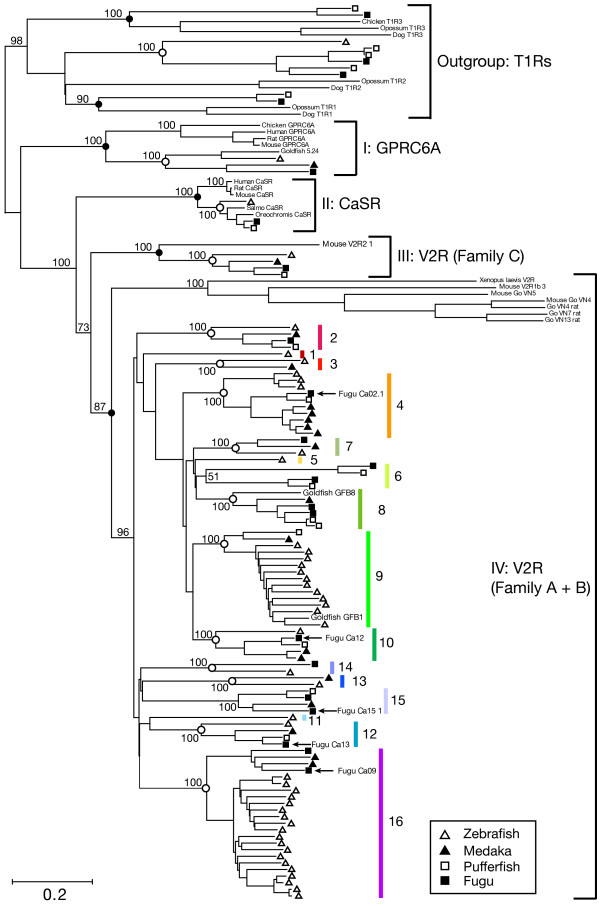
**Phylogenetic relationships of fish V2Rs and related GPCRs**. The tree is reconstructed by the neighbor-joining method with protein JTT matrix distances. Bootstrap values are shown on the major internal node only. The triangles and squares at the tips of the tree indicate species: open triangle, zebrafish; solid triangle, medaka; open square, pufferfish; solid square, fugu. Five fugu and two goldfish V2Rs used for constructing the profile HMM are shown in the tree. Solid circles at internal nodes indicate the position of the most recent common ancestor between teleost fishes and tetrapods. Open circles indicate the position of the most recent common ancestor among four fishes. Colors of each vertical bar indicate subfamilies in the fish V2R gene family. Genbank accession numbers of known V2Rs and related GPCRs used in this analysis are as follows: goldfish V2Rs (AF083080, AF083081), fugu V2Rs (AB008858–AB008862), mouse V2Rs (XP_144968, XP_145559, XP_142496), rat V2Rs (NP_775440–NP_775442), a V2R of *Xenopus laevis *(AB113361), CaSRs (AAF77923, AAT06805, AB008857, A56715, AAD40638, P48442), GPRC6As (AF158963, AY770492, XP_426177, NP_683766, CAD59483), mouse family C V2R (NP_064302, AAC08413).

Figure 3 shows a neighbor-joining (NJ) tree for 92 putatively functional fish V2Rs and several related GPCRs in vertebrates. The phylogenetic tree indicated that the divergences of V2Rs (family A+B) [9], Ca^2+^-sensing receptors (CaSRs), GPRC6A (fish 5.24 receptors and their putative orthologs in mammals [28-30]), and family C V2R (V2R2) receptors [9] predated the separation of tetrapods and teleosts (solid circles in Figure 3). In the NJ tree, the monophyly of each of these families was supported with high bootstrap probability. This result was also supported by maximum parsimony (MP) trees reconstructed by several representative V2R and related GPCR genes [see Additional file 1], although in maximum likelihood (ML) analysis, the monophyly of V2R (family A+B) was not supported [see Additional file 2]. However, this discrepancy may be attributed to the reduction of phylogenetic information of the dataset (In MP and ML analyses, only 57 OTUs were used out of 134 OTUs in full dataset). In NJ tree reconstructed by using full dataset, indeed, the monophyly of V2R (family A+B) was supported with a high boostrap probability (87%). All four fishes examined in this study had one CaSR, one GPRC6A, and one family C V2R, although for CaSR in medaka and GPRC6A in pufferfish, only partial sequences were found in their draft genome sequences [see Additional files 4 and 6]. In this article, we consider two V2R families, family A+B and family C V2Rs defined by Yang and colleagues [9], as distinct GPCR groups that diverged before the separation of teleosts and tetrapods (Figure 3). In the following, the term "V2R" is used simply to mean V2Rs family of the A+B.

Figure 3 also indicated that all fish V2Rs formed a monophyletic clade with high bootstrap probability (96%). We defined "subfamily" within the fish V2R family on the basis of phylogenetic grouping into clades that diverged before the divergence between the lineages of zebrafish and other fishes. Within the fish V2R family, 11 subfamilies could simply be recognized (2, 3, 4, 7, 8, 9, 10, 12, 13, 14, and 16; open circles in Figure 3). In addition, five monophyletic clades specific to zebrafish, or two or three of other fishes were found (1, 5, 6, 11, and 15). These clades also seem to have originated before the divergence of the lineages of zebrafish and other fishes, because the divergences of these clades predated those of adjacent subfamilies (Figure 3). Consequently, 16 virtually equivalent subfamilies could be identified within the fish V2R clade (Figure 3) and any intact fish V2Rs were included in one of these subfamilies. Almost all of these subfamilies could also be identified in the MP and ML trees [see Additional files 1 and 2].

In addition, to determine the gene subfamily to which each of "V2R pseudogenes" and "partial sequences" belongs, a BLASTP search was conducted against putatively functional fish V2R amino acid sequences by using the translated pseudogene sequences of fish V2Rs as a query. Query sequences were assigned to the subfamily to which the best hit of the query belonged with more than 80% amino acid identity. As a result, all V2R pseudogenes identified in this study could be assigned to one of the 16 subfamilies defined by phylogenetic analysis.

Table 1 shows the numbers of putatively functional genes and pseudogenes belonging to each V2R gene subfamily in the four fishes. The distribution of V2R genes in the 16 subfamilies substantially differed among species. Zebrafish has many genes belonging to subfamilies 9 and 16 (14 and 19 genes, respectively). Medaka has a relatively larger number of genes in subfamily 4 (7 genes and 7 pseudogenes). Fugu has a larger number of genes in subfamily 16 (7 genes and 1 pseudogenes), although some of these genes may be redundant because four out of seven genes are partial sequences [see 5]. Pufferfish have only up to three putatively functional genes in any subfamilies. Nine subfamilies (2, 4, 7, 8, 9, 10, 12, 14, and 16) were common in all fishes, when pseudogenes were taken into account (Table 1). Genes in subfamilies 1, 5, and 11 were zebrafish specific, and were not found in medaka, fugu, and pufferfish. In contrast, genes in subfamilies 6 and 15 were not found in zebrafish.

**Table 1:** Numbers of V2R genes belonging to different subfamilies in the four fishes.

Subfamily ^a^	Species			
	
	Zebrafish	Medaka	Fugu	Pufferfish
1	1 (0)	0 (0)	0 (0)	0 (0)
2	1 (2)	1 (0)	1 (0)	1 (0)
3	1 (0)	1 (0)	0 (0)	0 (0)
4	3 (0)	7 (7)	5 (0)	1 (4)
5	0 (2)	0 (0)	0 (0)	0 (0)
6	0 (0)	0 (0)	2 (0)	2 (0)
7	1 (0)	1 (0)	1 (0)	1 (0)
8	0 (1)	2 (2)	4 (5)	2 (0)
9	14 (1)	1 (0)	2 (2)	1 (1)
10	1 (1)	3 (3)	1 (0)	1 (0)
11	1 (0)	0 (0)	0 (0)	0 (0)
12	1 (2)	1 (0)	1 (0)	1 (0)
13	1 (1)	1 (0)	0 (0)	0 (0)
14	1 (0)	0 (2)	1 (0)	0 (1)
15	0 (0)	1(0)	4 (0)	1 (1)
16	19 (1)	2 (3)	7 (1)	0 (4)
family C	1 (0)	1 (0)	1 (0)	1 (0)

Total	46 (11)	22 (17)	30 (8)	12 (11)

Number in parentheses indicates the number of pseudogenes.

^a ^Subfamilies of V2Rs are defined in the neighbor-joining tree shown in Figure 3 (see Results).

### Genomic distributions of V2R genes in zebrafish, medaka, fugu, and pufferfish

**Figure 4:**
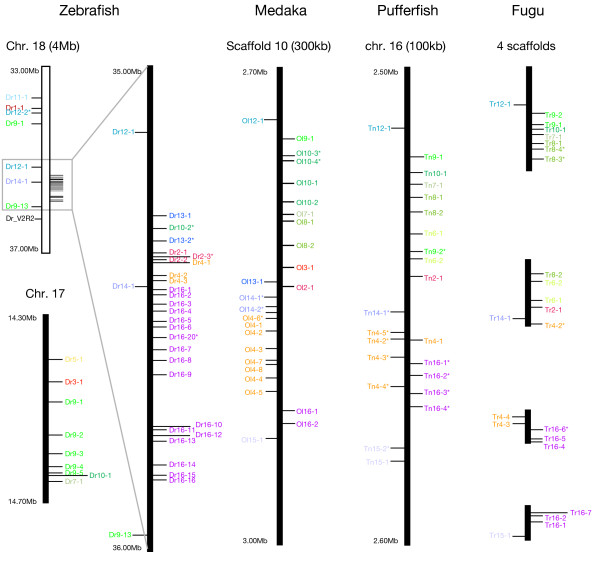
**Chromosomal locations of V2R genes in zebrafish, medaka, pufferfish, and fugu**. Each subfamily is indicated in color as in Figure 3. Asterisks indicate pseudogenes. Note that the scale of the V2R cluster is different among species. The length of the V2R cluster region in each species is shown in parentheses. In fugu, only four representative scaffolds that contain V2R genes are shown.

Physical maps of V2R gene clusters in the four fishes are shown in Figure 4. Most zebrafish V2R genes were located on three different chromosomes, 5, 17, and 18. On chromosome 5, two putatively functional V2R genes were found at ca. 1 Mb intervals, with the same transcriptional orientation (not shown in Figure 4). Both of these genes belonged to subfamily 16. On chromosome 17, nine V2R genes and pseudogenes were encoded within a 400 kb region. All genes shared the same transcriptional orientation. The largest V2R gene cluster was found on chromosome 18. Thirty-four zebrafish V2R genes/pseudogenes and one family C V2R gene were located within a 4 Mb region on chromosome 18. Twenty-nine genes and pseudogenes in this region were concentrated within a 1 Mb region (Figure 4). On the other hand, in medaka and pufferfish, most of their V2R genes and pseudogenes found in this study were encoded in one chromosomal region. In medaka, 25 V2R genes and pseudogenes were clustered within a 300 kb region of scaffold 10 of its draft genome sequences. Surprisingly, in pufferfish, 22 V2R genes and pseudogenes were clustered within only a 100 kb region of chromosome 16. Similarly, in fugu, most of the V2R sequences may also be encoded in a single gene cluster with high density, although they were separated into several different scaffolds. A fugu V2R gene cluster was reconstructed by referring to that of pufferfish (Figure 4), but the true relationship across the scaffolds has not been known so far. In fugu, only four representative scaffolds containing V2R genes are shown. In all fishes examined, genes in the same subfamily tended to be located close to one another (e.g. subfamilies 4 and 16), suggesting tandem gene duplication as the primary mechanism for expansion of the gene family.

Comparison of the gene clusters among four fishes revealed similarities in the arrangements of fish V2R genes in the gene subfamily level (Figure 4). First, the arrangement of V2R genes in the middle of the cluster begins with subfamilies 2, 14, 4, and end with subfamily 16, in all four fishes. Second, the position and orientation of one subfamily 12 gene also seems common to all species. Such concordance is likely to reflect the gene order of the V2R cluster in the common ancestor of the four fishes.

### Comparison of the genomic region containing V2R gene cluster in fish with corresponding regions in tetrapods

**Figure 5:**
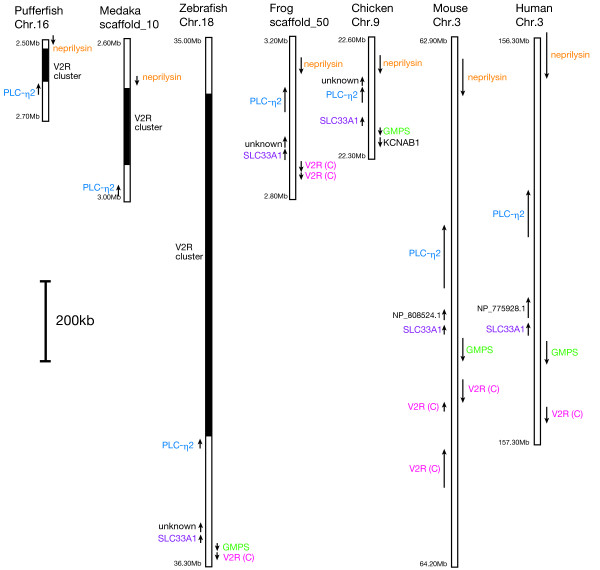
**Genomic region around the V2R gene cluster in fishes and the corresponding region in tetrapods**. The fish V2R cluster is shown in black. Positions and transcriptional orientations of genes are indicated by arrows. V2R (C) indicates the family C V2R gene.

To find the tetrapod genomic region corresponding to the fish region that contains the V2R gene cluster, putative orthologs of several genes that are commonly located proximal to fish V2R gene clusters were identified in tetrapod genomic sequences (Figure 5). Initially, we characterized *neprilysin*, *phospholipase C-η2 *(*PLC-η2*), *acetyl coenzyme A transporter 1 *(*SLC33A1*), and *GMP syntase *(*GMPS*) homologs in fish genomes, near the fish V2R gene cluster. The V2R clusters in pufferfish and medaka were located between *neprilysin *and *PLC-η2 *(Figure 5). In zebrafish, *PLC-η2 *was found near the V2R gene cluster in chromosome 18, but the *neprilysin *homolog was not found. On the other hand, *SLC33A1 *and *GMPS *were identified near the family C V2R gene on zebrafish chromosome 18, but not in medaka scaffold 10 and pufferfish chromosome 16. Subsequently, putative orthologs of these four genes were identified in the tropical clawed frog (*Xenopus tropicalis*), chicken (*Gallus gallus*), mouse (*Mus musculus*), and human (*Homo sapiens*) genomes. All four genes (*neprilysin*, *PLC-η2*, *SLC33A1*, and *GMPS*) were tandem linked on specific chromosomal regions in all tetrapod species examined (Figure 5). The relative positions and orientations of these genes in fishes were well consistent with those in tetrapods, indicating that the synteny of the genomic region is highly conserved between teleosts and tetrapods. However, the V2R gene cluster located between *neprilysin *and *PLC-η2 *genes in fishes was not found in any tetrapods. On the other hand, one or more family C V2R genes near *SLC33A1 *and *GMPS *genes were observed commonly in zebrafish, frog, mouse, and human genomes, although not in the chicken genome.

## Discussion

### Large variation of V2R gene repertoire among fish species

In this study, we identified the V2R gene repertoire in fishes by searching zebrafish, medaka, fugu, and pufferfish draft genome sequences. Our results provide the first global outline of the fish V2R gene repertoire. In this study, we showed that the numbers of functional V2Rs varied substantially among fishes. We identified 46 putatively functional V2R genes in zebrafish, that were much more than in other fishes (22 medaka; 30 fugu; 12 pufferfish). The larger number of V2R genes in zebrafish is mainly attributed to species-specific gene duplication events in subfamilies 9 and 16 (Figure 3). Table 2 shows the numbers of putatively functional chemical receptor genes identified in the four fishes. The size of the putatively functional V2R repertoire varies by 3.8-fold among the four fishes. This size variation of fish V2R is larger than that of OR (2.4-fold, Table 2). In addition, the numbers of fish V1R and taste receptors (taste 1 and 2 receptors) are almost the same in all fishes (Table 2); fish V1R gene, in particular, is a single copy in all fishes examined [12]. Thus, the V2R family is considered to be the most variable chemical receptor gene family in fishes. Such a high variation in sizes of the V2R gene family is also observed in mammals. Sixty one and 57 functional V2R genes have been identified in the mouse and rat, respectively [9], whereas no functional V2R gene has been found in human and other primates [34].

**Table 2:** Numbers of odorant/pheromone receptors, taste receptors, and trace amine receptors identified in the four fishes.

			Species				
				
GPCR gene family	Type^a^	Family size variation (ratio)	Zebrafish	Medaka	Fugu	Pufferfish	Sources
Odorant/pheromone receptors	V2R	3.8	46	22	30	12	This study
	V1R	1	1	1	1	1	[12]
	OR	2.4	102	-	44	42	[13, 14]
							
Taste receptors	T1R	1.3	4	5	4	5	[31, 32]
	T2R	1.5	4	-	4	6	[31]
							
Trace amine-associated receptor	TAAR	7.1	57	-	8	-	[33]

Only putatively functional genes are considered. The numbers of ORs, T2Rs, and TAARs in medaka, and TAARs in pufferfish have been unknown.

^a ^V2R, vomeronasal receptor family 2; V1R, vomeronasal receptor family 1; OR, main odorant receptor; T1R, taste 1 receptor; T2R, taste 2 receptor; TAAR. trace amine-associated receptor.

Recently, the trace amine-associated receptor (TAAR) family has been shown to be used as odorant receptors in mice [35]. Homologs of TAAR genes are also found in fishes [33], and some of them are suggested to be expressed in the olfactory epithelium in zebrafish [35]. Therefore, also in fishes, TAAR family is likely to be used for perception of odor chemicals. Interestingly, the size of the functional TAAR repertoire varies by 7.1-fold between zebrafish and fugu (Table 2). It is possible that the differences of V2R and TAAR gene repertoires mainly cause the difference of odor sensitivity among fish species.

### Evolution of the fish V2R gene family

Phylogenetic analysis of V2Rs and several "V2R-related" GPCRs (family 3 GPCRs) [36] revealed that the four GPCR families, GPRC6As, CaSR, family C V2R, and V2Rs (family A+B), were separated before the divergence of teleost fish and tetrapods (Figure 3). In fishes, GPRC6As and CaSRs appear to be functionally distinct from V2Rs (family A+B) because their expression profiles seem different from the fish family A+B V2R genes. It is known that in fish, CaSR is expressed mainly in the kidneys and functions as a salinity sensor [37,38]. In goldfish, 5.24 receptor (GPRC6A ortholog) is expressed in olfactory cells, responding to naturally occurring amino acids [28,29]. However, goldfish 5.24 receptor is expressed not only in the olfactory epithelium, but also in the gills, tongue, and other tissues [28]. In human, GPRC6A is reported to be widely expressed in the brain and peripheral tissues with highest levels in the kidney, skeletal muscle, testis, and leucocytes [30]. Additionally, GPRC6A and CaSR were single copy genes in the four fishes. This also suggest that these receptors are not involved in the distinction of the diverse array of odor chemicals. Fish family C V2R is also a single copy gene in all four fishes (Table 1), and thus, may be functionally distinct from other fish V2Rs (family A+B); however, the expression of the fish family C V2R is not yet known and further studies are needed.

The present phylogenetic analysis also indicated that fish V2Rs were further subdivided into at least 16 subfamilies that diverged before the divergence between the lineages of zebrafish and three other fishes (Figure 3). The number of V2R genes in each subfamily was remarkably different among species as discussed above. Generally, the evolution of vertebrate odorant and vomeronasal receptors is characterized by rapid gene turnover and lineage-specific phylogenetic clustering [8,9,13,39]. These characteristics are also observed in fish V2Rs. Indeed, the phylogenetic tree shows that fish V2R genes tend to form species-specific gene clades (e.g. zebrafish subfamilies 9 and 16, medaka subfamily 4; Figures 3 and 4). These V2R subfamilies unique to each species may be related to the adaptation to species-specific environmental odor and/or pheromonal signals. On the other hand, almost one-to-one orthologous relationships were found among the four fishes in four subfamilies, 2, 7, 10, and 12 (Figure 3). These V2Rs may be used to perceive common odor substances to these fishes, like amino and nucleic acids [21].

In fishes, most V2R genes were located on one specific genomic region as a gene cluster (Figure 4). Interestingly, the arrangements of V2R genes in the cluster appear to be well conserved among species at the gene subfamily level (Figure 4). This suggests that the ancestral fish V2R gene cluster was already present in the most recent common ancestor of the four fishes. In zebrafish, two gene clusters were found on chromosomes 17 and 18. Two indirect pieces of evidence strongly suggest that the two zebrafish V2R gene clusters originated from one chromosomal translocation occurred in the zebrafish lineage. First, genes in each of these clusters were not phylogenetically clustered (Figure 3). Second, a genomic region near one gene in subfamily 12 (12-1) that contains several genes in subfamilies 7, 9, and 10 were lacking only in the zebrafish chromosome 18 gene cluster (Figure 4), and these genes were located on the chromosome 17 gene cluster (Figure 4).

From the synteny of *neprilysin*, *PLC-η2*, and several genes between fishes and tetrapods, we could identify the genomic region of tetrapods that corresponds to the fish genomic region containing the V2R gene cluster (Figure 5). Physical maps of this region (Figure 5) show that the V2R gene cluster located between *neprilysin *and *PLC-η2 *is fish-specific and are not found in tetrapods. This indicates that the V2R gene cluster in fishes has been originated after the separation of teleost and tetrapod lineages. The maps also show that one or more family C V2R genes are located near *SLC33A1 *and *GMPS *genes commonly found in the zebrafish, frog, mouse, and human in common. This suggests that the family A+B of fish V2Rs has been originated by a tandem duplication of the ancestral V2R gene occurred before the divergence of teleosts and tetrapods (Figure 3), and the family C of V2Rs is the sister of the family A+B (see also Figure 3). This may also indicate that one of the ancestral V2R family A+B located in the genomic region between *neprilysin *and *PLC-η2 *was increased in their number by tandem gene duplications, only in the lineage of teleosts, but not of tetrapods.

In the last part of this section, we summarize the long-term evolutionary scenario of the fish V2R gene family. Before the divergence of teleost fish and tetrapod lineages, an ancestral V2R (family A+B) gene and family C V2R gene had diverged from one common ancestral V2R gene by tandem gene duplication. After teleost-tetrapod divergence (ca. 480 MYA; Figure 1) [27], in the common ancestor of the four fishes, prototypes of fish V2R gene subfamilies observed in the present fishes (Figure 3) had been generated by tandem gene duplications from the ancestral V2R (family A+B) gene. Then, these prototype V2R genes formed a gene cluster. During the evolution of the lineages of the four fishes, gene duplications and losses occurred in each of these lineages. In the zebrafish lineage that had diverged from other fish lineages at ca. 320 MYA, the V2R gene cluster separated into two, and genes in subfamilies 9 and 16 have increased their numbers within the gene clusters in chromosomes 17 and 18, respectively. In the medaka lineage (diverged from fugu and pufferfish lineage at ca. 190 MYA), subfamily 4 genes have increased their copies within the gene cluster. In the fugu and pufferfish lineage, it appears that V2R sequences in some subfamilies are pseudogenized only in the pufferfish (Table 1). For instance, in subfamily 4, fugu has five putatively functional genes, but in pufferfish, four out of five are pseudogenes. Similarly, in subfamily 16, fugu has seven genes and one pseudogene, whereas pufferfish has four pseudogenes and no functional gene. This implies that, in the pufferfish lineage, after the separation from the fugu lineage (ca. 85 MYA), some V2Rs might lose their function by some biological reasons, such as the adaptation to freshwater environment. In the future, to elucidate the more detailed evolutionary process of V2R (family A+B) gene family, data from nonteleost fishes, namely "ancient fish" [40], seem to be useful. In addition, to clarify the divergence period of V2R family to the other family 3 GPCR families, examination of Elasmobranchii fishes (i.e. sharks and rays) may be one of important steps of studies.

### Functional significance of the genes located near the fish V2R gene cluster

Two protein-coding genes, *neprilysin *and *PLC-η2*, are located near the fish V2R gene cluster (Figure 5). Interestingly, it appears that the products of these genes can interact with V2Rs. Langenau and colleagues [41] reported that the transcription of *neprilysin *was upregulated in the ovary of yellow perch (*Perca flavescens*), incubating it with 17α, 20β-dihydroxy-4-pregnen-3-one (17, 20-P) that stimulated fish ovulation. Neprilysin is a membrane-bound neutral peptidase expressed in various tissues [41]. Some peptides that are cleaved by neprilysin may be released into the water from individuals and spawned eggs, and perceived by some V2Rs. Such peptides might be used as a signal in reproduction and/or identity in fishes. Recent studies in mammals have indicated that V2Rs are receptors for peptide sex pheromones [22] and MHC ligands [23]. These studies may be consistent with the hypothesis that fish V2Rs are also used to recognize peptides that serve as reproductive and/or individuality signals. It is also interesting that 17, 20-P is one of the components of the pre-ovulately pheromone which affects male goldfish [42].

PLC-η2 is one of the η-type phospholipase C, localized specifically in neurons. V2Rs are included in the family 3 GPCRs, which is known to be coupled with phospholipase C [36]. Western blot analysis showed that, in mouse, PLC-η2 protein was not localized in the olfactory epithelium or the vomeronasal organ [43]. This suggests that PLC-η2 is not coupled with V2Rs; however, it was also shown that PLC-η2 was localized in the main- and sub-olfactory bulbs [43]. This implies some functional relationship between PLC-η2 and the odorant recognition system in vertebrates.

The close linkage between the genes encoding V2Rs and neprilysin/PLC-η2 in fishes seems to be an intriguing finding in light of the function of fish V2R genes. Functional studies of neprilysin and PLC-η2 could provide further information on the evolutionary mechanisms of fish V2Rs.

## Conclusion

In this paper, we have presented the repertoire of V2R gene family in the four teleost fish species. The number of intact V2R genes in these fishes varied from 12 in pufferfish to 46 in zebrafish. Phylogenetic analysis has shown that the evolution of fish V2Rs are characterized by rapid gene turnover and lineage-specific clustering, as known in ORs and V1Rs [8,9,13,39]. Such evolutionary patterns may suggest that fish V2Rs are involved in the lineage-specific adaptation to different odor environments for each fish species. In addition, we elucidated substantial parts of the long-term evolution of fish V2R gene family, by phylogenetic and comparative genome analyses. We believe that our results provide one of the foundation to the future functional and evolutionary studies of fish V2R gene family.

## Methods

### Identification of fish V2R genes from draft genome sequences

The 6.5× coverage of the zebrafish genome sequence (Zv.5), 8.7× coverage of the fugu genome sequence (Fugu version 4), and 8.3× coverage of the pufferfish genome sequence (Genoscope Tetraodon 7) are available at ENSEMBL [44]. The 6.7× medaka genome sequence is available from the National Institute of Genetics (NIG) DNA Sequencing Center, Mishima, Japan [45]. First, a TBLASTN search was conducted with E-value 10^-10 ^against the genomic data by using the TM domain of several representative V2R amino acid sequences known in fugu, goldfish, and zebrafish as queries. Obtained sequences were confirmed by BLASTP searches against the NCBI non-redundant (nr) database. In each of these sequences, if the best hit in this search was a previously known fish V2R, it was considered a V2R sequence. Second, each region of BLAST similarity was extended in 5' and 3' directions to perform a detailed prediction of V2R coding sequences. Each region of BLAST similarity was extended 4 kb in the 5' and 1 kb to the 3' direction. Gene prediction, described as follows, was conducted in each of these extended regions. After the first round of gene prediction, if the total coding sequence of the V2R gene was not included in this region, the 5' sequence was further extended up to 10 kb.

For each of these regions, V2R coding sequences were estimated based on the profile HMM-based gene prediction with the program WISE2 [46]. Seven full-length fugu [16] and goldfish [10] V2Rs were aligned using the program ClastalW [47] with the default settings. A profile HMM was constructed from the alignment by using the program HMMER software package [48], and used for gene prediction. In addition to V2R (families A+B and C) homologs, CaSRs and a homolog of goldfish 5.24 amino acid receptors (GPRC6A) [28] were also identified. These "V2R-related" GPCR family 3 receptors were included in phylogenetic analysis. The obtained putative protein sequences were examined by the TMHMM method [49] for the presence of seven transmembrane domains. Chromosomal positions of putative V2R genes and pseudogenes in zebrafish and pufferfish could be determined by mapping them onto chromosome contigs. The list and sequences of fish V2Rs are available as supplementary information [Additional files 3, 4, 5, 6, 7, 8, 9, 10].

### Phylogenetic analysis

Deduced amino acid sequences of 90 putatively functional V2R genes (including family C V2Rs) in zebrafish, medaka, fugu, and pufferfish and several related GPCRs (CaSRs, GPRC6As, and T1R taste receptors in some vertebrates) were aligned by using the program FFT-NS-i (MAFFT 5.731) [50] and slightly modified by eyes. Seventeen vertebrate T1Rs were used as outgroups. Phylogenetic tree was constructed using the neighbor-joining method [51] with JTT matrix distances [52] implemented in the program MEGA 3.1 [53]. The reliability of each tree node was assessed by the bootstrap method with 1,000 replications. The MP and ML trees were reconstructed by using reduced dataset consisted of 45 representative fish V2R (including family C) genes, three tetrapod V2R genes, and nine related GPCR family 3 genes, because in these methods, it is virtually impossible to reconstruct trees from the full dataset (134 OTUs). Initially, an alignment of nucleotide sequences of these genes were constructed based on the alignment of corresponding amino acid sequences. For the ML methods, the optimal model of sequence evolution was determined to be the Tamura-Nei + I + gamma model by the hierarchical likelihood-ratio test implemented in MODELTEST 3.7 [54]. The ML tree was searched by a heuristic-search algorism implemented in PAUP 4.10 b [55]. Similar to the ML tree reconstruction, the MP tree was also obtained by a heuristic-search algorism implemented in PAUP 4.10 b. The reliability of MP tree nodes was assessed by the bootstrap method with 1,000 replications. In the MP and ML trees, the fugu T1R1 sequence was used as an outgroup.

### Identification of tetrapod genomic regions corresponding to the region in fishes with the V2R gene cluster

Initially, several protein-coding genes were characterized around V2R gene clusters in zebrafish, medaka, and pufferfish, by using ENSEMBL genome browser [44]. Fugu was not included in this analysis because fugu V2R clusters were separated into at least four unconnected scaffolds and thus we could not identify the gene order around V2R clusters. Next, putative orthologs of these genes were identified in the genomic sequences of the frog, chicken, mouse, and human by TBLASTN searches. The identities of these putative orthologs were confirmed by "reciprocal best hits" of TBLASTN searches between fishes and tetrapods.

## Authors' contributions

YH carried out data mining, data analysis, and manuscript preparation. MN supervised YH and finalized the manuscript. Both authors read and approved the final manuscript.

## Supplementary Material

Additional File 1 **Maximum parsimony tree of 45 representative V2R genes (including family C V2Rs) and related family 3 GPCR genes of zebrafish, medaka, fugu, and pufferfish**. Fugu T1R1 is used as an outgroup sequence. Bootstrap values higher 50% are shown on interior branches. The first two letters in each OTU indicates species: Dr, zebrafish (*D. rerio*); Ol, medaka (*O. latipes*); Tr, fugu (*T. rubripes*); Tn, pufferfish (*T. nigroviridis*). Click here for file

Additional File 2 **Maximum likelihood tree of the 45 representative V2R genes and related family 3 GPCR genes in the four fishes**. Fugu T1R1 is used as an outgroup sequence. Click here for file

Additional File 3 **Name, chromosomal position, transcriptional orientation, and functionality of each V2R sequence in zebrafish**. In the "functionality" column, F and P represent putatively functional gene and pseudogene, respectively. A numeral after "F" indicates the number of disruption in the sequence. "Partial" indicates that the V2R sequence is putatively functional but its length is less than 2,400 bp. Click here for file

Additional File 4 Name, chromosomal position, transcriptional orientation, and functionality of each V2R sequence in medaka. Click here for file

Additional File 5 Name, chromosomal position, transcriptional orientation, and functionality of each V2R sequence in fugu. Click here for file

Additional File 6 Name, chromosomal position, transcriptional orientation, and functionality of each V2R sequence in pufferfish. Click here for file

Additional File 7 Deduced amino acid sequences of zebrafish V2Rs. Click here for file

Additional File 8 Deduced amino acid sequences of medaka V2Rs. Click here for file

Additional File 9 Deduced amino acid sequences of fugu V2Rs. Click here for file

Additional File 10 Deduced amino acid sequences of pufferfish V2Rs. Click here for file
