# Supplementary figures and images for: Evolution and origin of vomeronasal-type odorant receptor gene repertoire in fishes
Source: BMC Evol Biol. 2006 Oct 3;6:76. doi: 10.1186/1471-2148-6-76 (PMC1601972; doi:10.1186/1471-2148-6-76)

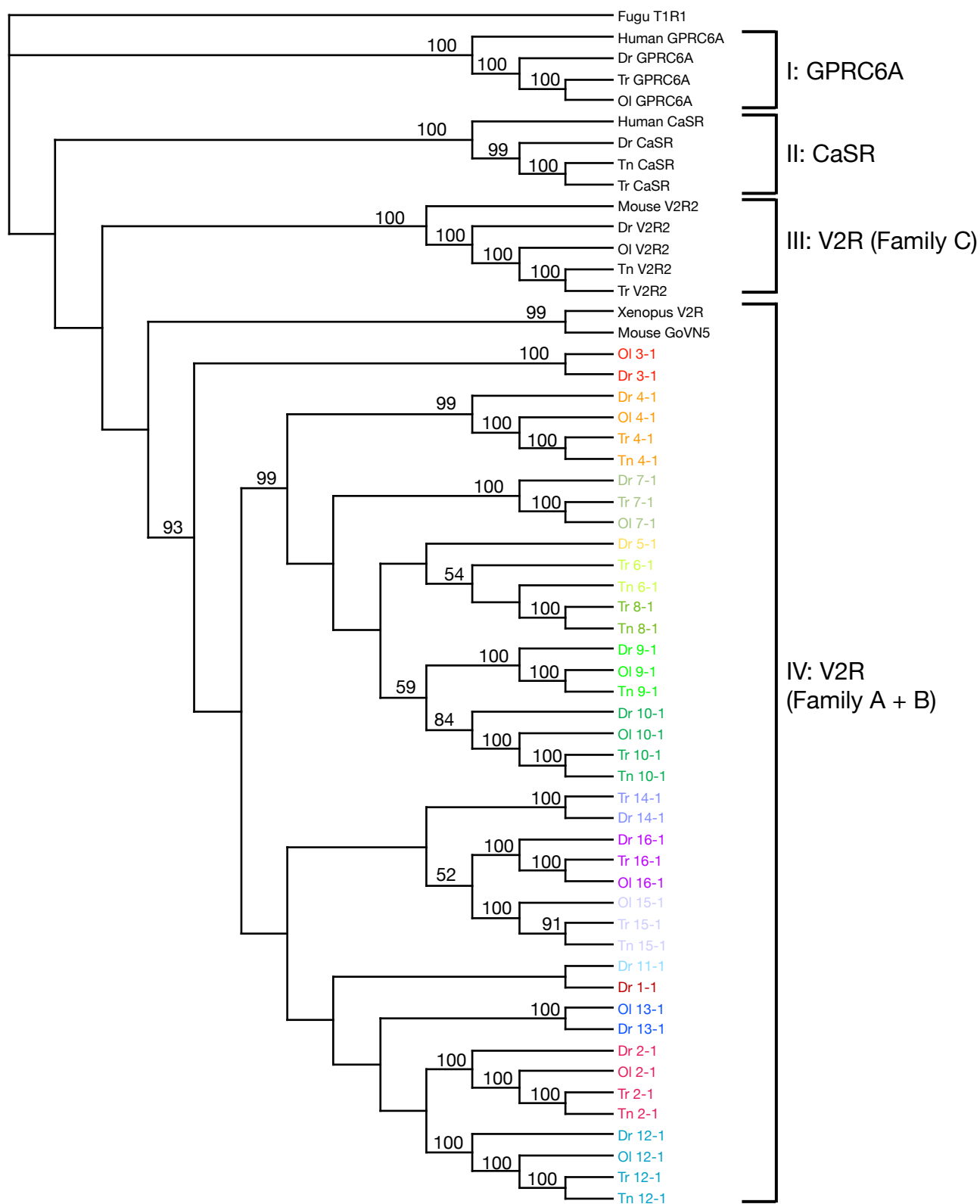

Supplement: Additional File 1 — Maximum parsimony tree of 45 representative V2R genes (including family C V2Rs) and related family 3 GPCR genes of zebrafish, medaka, fugu, and pufferfish. Fugu T1R1 is used as an outgroup sequence. Bootstrap values higher 50% are shown on interior branches. The first two letters in each OTU indicates species: Dr, zebrafish (D. rerio); Ol, medaka (O. latipes); Tr, fugu (T. rubripes); Tn, pufferfish (T. nigroviridis). [file 1471-2148-6-76-S1.pdf]

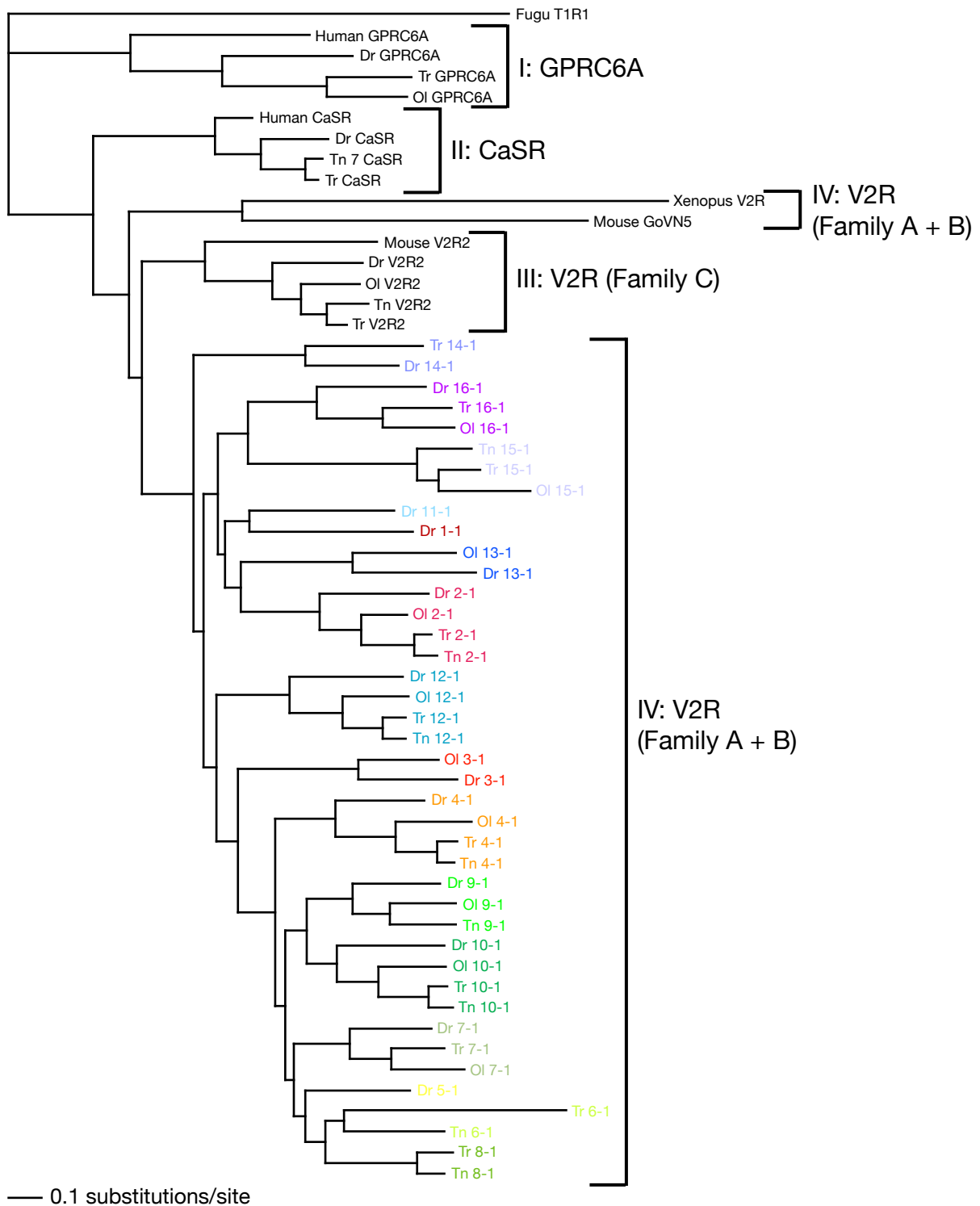

Additional file 2

Supplement: Additional File 2 — Maximum likelihood tree of the 45 representative V2R genes and related family 3 GPCR genes in the four fishes. Fugu T1R1 is used as an outgroup sequence. [file 1471-2148-6-76-S2.pdf]
