# Supplementary material for: Evolution and origin of vomeronasal-type odorant receptor gene repertoire in fishes
Source: BMC Evol Biol. 2006 Oct 3;6:76. doi: 10.1186/1471-2148-6-76 (PMC1601972; doi:10.1186/1471-2148-6-76)
Supplement: Additional File 7 — Deduced amino acid sequences of zebrafish V2Rs. [file 1471-2148-6-76-S7.pdf]

>Dr_16_17_F

MWVILNISIYLIFNFIMSASILGSNTCQLQGHFRLNEMYHDGDVILGGLFEVDFLTVFPDLDFK
TEPEPPYCVQFDMESFQQAQTMAFAIDEINKNPNLLPNITLGYHLFDNCVMLGMAFRAAISM
ASGTEEFFNNINCTGPPPVIGIVGDSSSTVSIAISSILGLFRVPIVSHYATCSCLSDRKKYPSFFR
TIPSDAFQVRAMVQILKYFRWTWVGLIYSDDDYGVYAAQSFQQEMQLFGGCVAFSEVLPHD
NNLEDIKHITKMIQASTARVIVVFSTPSFLIPLIDELVLQNMTDRQWIASEAWATSFVHHSPRLL
PFLKGTLGIAIRRGEIQGLYEFLLRLQPTNDPKNNMIRIFWEIMFGCNFETGGKKNSGDQVINI
CTGQEDLRTTKTPYTEVSELRASYNVYKAVYALAHALNDLMQCEDGRGPFSVNSCADITNL
KPWQLVHYLQNVNFTTDFGDHVSFDKNGDALAIYDVLNWQPSSDGSIRAQTVGVVNEEVAT
GMVLTLDEDALYWNFESKKVNNTPPSSVCSESCAPGTRLATIKGLPVCCFDCLPCGDGEISN
ITGAVECTMCPDEFWSNLDKDQCIPKEVEFLSYEDPLGISLTTASLLGTCFCVLVMFVFAVHR
NTPIVRANNSELSFLLLLSLKMCFLCALLFIGQPQLWTCQLRHVVFGISFVLCISSILVKTMVVI
AVFKSSRPESKGAMKWFGAVQQRCTVLVLTAIQVVICAVWLSTASPAPHKNNQYIRSKIVYEC
AIGSVAGFSMLLAYIGLLAAVSFLLAFLARNLPDNFNEAKFITFSMLIFCAVWIAFVPAYVSSPG
KYAVAVEIFAILASSFGLLLAIFAPKCYIILLHPERNTKKAIMGKE

>Dr_16_18_F

MWITLCIYLYLSLNYICAHSILRTSDSCRLQRRFHLNGMYKDGDVILGGLFQVHFFTVFPEQS
FRTEPEPPFCEKFDMESFQQAQTMAFAIEEINKNPNLLPNITLGFHLYDNCVRLGMAFRAAIS
LASGTEESFQNLNCTGPPPVIGIVGDPSSTPSIAISSLLGLFRVPIVSYYATCSCLSDRKKYPSF
FRTIPSDAFQVRAMVQILRHFGWTWVGLIYSDDDYGVYAAQSFHLEMQMFGHCVAFSEILP
HNNNQRDIQRIMGVIQASTARVVVVFSTSSFLLPLIDEVASQNMTGRQWIASEAWATSPVYH
SPRLLPFLGGTLGIAVRHGEIPGLHDFLLHLGPGNESRNNMLRIFWENMFGCSFEPGVKDT
NSEIKLCTGQEDLSTTNTPYTDVSGLRAAYNVYKAVYALAHGLHDLMQCEKGRGPFRGESC
ADITNLKPWQLVHYLQKVNFTTGFGDHVSFDENGDALAIYDVLNWQPSSDGSIQIFTVGVVK
ERTETGMVLTIDEDAIYWNFETKKSPQSVCSESCSPGTRRATRKGLPVCCFDCLPCRDGEIS
NTTDAIECMLCPDEFWSSPDKDQCVPKEVEFLSYEDPLGISLTTASLLGSCFCVLVMIIFGLH
RNTPIVRANNSELSFMLLLSLKMCFLCVLLFIGRPQLWTCQLRHAVFGISFVLCISSILVKTMV
VIAVFKSSRPEGKGAMKWFGAAQQRCTVLVLTVLQVVICSVWLSTASPTPYKNNQYIRSKIVY
ECAIGSVAGFSLLLGYIGLLAAVSFLLAFLARNLPDNFNEAKFITFSMLIFCAVWIAFVPAYVSS
PGKYAVAVEIFAILASSFGLLVAIFAPKCYIILLHPERNTKNAIMGRE

>Dr_5_1_F

DVTIGGIFSFHNSWEEIMPTFTSKPEQPKCKSLSLREFQNAQTMIYAIEEINKRADILPGLSLG
YRIFDSCGSIEMALRASLSLVNGENASHLSCQRPKTVQAIIAETSSTPTIAISATVGPLHLPVIS
HFATCACLSDRKKHPSFFRTVPSDYYQSRALAKLVKYFGWTWVGALCSDNDYGNNGMNTF
IKAATEFGVCVEFSEAFFRTHPREEILRIVDVVKKSSSKVIVAFVSYSDMEVLLLELAKQNITGL

QWIGSESWISDMNIATGKWQYILRGSMGFAIPKAEIQGLREFLIKIHPSSNIYLYKELWESVFQ
CRLSTEQSSESKSMCTGNESLNNVQNQYTDVTELQIANNVYKAVFAIAHALNSSVGCSKWD
TNQRECNTWQVLQALREVSFFTETGEKVFFDKNGDPAARYDLLNWQQGEEGATKFVKVGF
YDASLQPEFQLSFNNITIMWAKNQHQVPVSVCSESCPMGTRKAVKKGKPLCCYDCIQCAE
GEISNKTDSVMCLKCPPEFWSNKWRDTCVPKLVEFLSFEDVMGIVLIIFSLLGVSFTLGIAIIFF
VHKDTPIVKANNSELSFLLLFSLTLCFLCSLTFIGRPTEWSCMLRHTAFGITFVLCISCVLGKTI
VVLMAFKATLPGSNVMKWFGPLQQKLSVITFTLLQVVICVLWLTLSPPFPYMNMNYYQERIIL
ECNLGSAFGFWAVLGYIGLL
ILCFILAFLARKLPDKFNEAKFITFSMLIFCAVWITFIPAYVSSPGKFTVAVEIFAILASSFALLFCIF
APKCYIILLRPEENTKKHMMSKS
>Dr_3_1_F
MKNFNILLFCSLLYVQTSSMCQLLRTFEMPNIFKVGDIMIGGIFPIFNKQENIIGSFERNMQRIK
CTGFDLRAFRWIQSMMFAVDEINKNEELLPHISLGYKIMDSCASPTNVLRAVFSLVSEQKEQE
FTSQCHLPLTALVAESGSSQSLAVAGMLGPFRVPMVSYFSTCACLSDRKKYPSFFRTIPSDF
YQAKALASLVKQFGWTWIGALQSDNDYGRNGISAFTKEVEKMGVCIAFVGTILRTYPQSKITE
VVEMIKESTVKVILAFVPEGDLYPLMKEVVNRNITGIQWIASEAWVTAARPSTPEMFKSFGGT
VGFVVRKMAMLKLRPYLENISPYSPTQSAFVSDFWETVVGCKPCLNCEPSANSTLNGQMC
TGQEKLTFTDKFFDVTQVRVTYNVYQAVYAIAHAIHKVLYCQGDNNLSKMCLNVSQITPKQVS
DQLERVNFIDEYGENVFFDENGDPPASYELINWQLNQGEVQHVTVGYFSKSPDGTYKLTIKE
DNVHWSTENLIPKAVCSDTCPKGTRKAQIKGRPVCCFDCIPCADGSISNTTGAADCTLCPKE
YWSNERRDKCLVKTIEFLSYTETMGIILTALSLFGASLTVATMIVFIHYRETPIVKANNSELSSLL
LSSLFFCFLCPLTFIGEPTHWSCMLRHTAFGLTFALCISCVLGKTIVVVTAFRATLPGNKLSGK
FGPVQQRAIVFLCTAIQIVICALWLLIKPPFPDKALRYDHKKIILECNTGSDAGFYAALGYVGLL
STICLILAFLGRKLPDNFNEAKFISFSMLIFCAVWVTFIPAYISSPGKYTVAVEIFAILSSAFGLLL
CIFAPKCFIILIKPERNTRKHVMGKS
>Dr_9_1_F
MLLFLYIFLHFTLIRTKAGNGLCQMMGDPKYPLFSKDGDITIGALFAILSKETLPSFQFTQKPQ
LLSCSSVNLRDFRMTQIMIFAIEEINRSKSLLPNVSIGYRIYDTCGSRMSAMSATMALMNGPE
FTADKICNGESPIHAIIGETESSATIILSKTTGPFKIPVISHSASCECLSNRKIYPSFFRTIASDYH
QGRALAFIVKHFGWSWVGTVNSDNDYGNNVMAIFLNTAQKEGICVEYSVKFYRTEPEKLKK
VVETIKKSTAKVIVAFVSLVEMGLLIDQLSIQNITGFQVIGGEGWITTKSLINSKSFHVLGGSLGF
AFRKIIIEGFADYVIKTFWERDFPCLTNEGNYSQYALTCSSYQDLFTLKHYNEDIPEQRYASNV
YKAVYAVAHSLHNLLKCKENEGCEKSLTMQPQQVVEALKNVNFTLKFGDRVWFDSTGGAVA
HYEVVNWQQDSDGSFQFKKVGYFDASLPPDQSFMLNTKNIIWSGGQLERPRSVCSESCPP
GTRKASQKGRPVCCYDCIPCAEGEISNETDSVNCKQCQGEYWSNVEKNKCVLKAVEFLSFT

DFMGIVLVFFSLFGVGLTVLVAILFYSKKDTPIVKANNSELSFLLLLSLTLCFLCSLTFIGRPTEW
SCMLRHTAFGITFVLCISCVLGKTIVVLMAFKATLPGSNVMKWFGPVQQRLSVLAFTLIQVLIC
VLWLTMSSPFPYKNMKYYKEKIILECSLGSVIGFWAVLGYIGLLAALCFILAFLARTLPDNFNE
AKFITFSMLIFCAVWVTFIPAYVSSPGKYTVAVEIFAILASTFGLLFCIFVPKCHIIIFKPEQNTKQ
HMMGKN
>Dr_9_2_F
MLFFLHTLPLLYQLNTNTPCQTMGDPKDPLFSKDGDVTIGGVFAIHRKETLPSFEFAQKPLPL
SCSSVNLRDFRLAQIMIFAIEEINKSKSLLPNIYIGYRIYDTCGSRLSTMSATMAVLSGQEFRPR
DRCNDQPPLHAILGESESSATVILSRTTGPFKVPVISPSATCECLSNRKDYPSFFRTIASDYHQ
SRALAYIVKHFGWTWVGAVSSDNDYGNNGMAIFQKIAQEEGICVEYSVKFYRTESDKLKKVV
NEIKKGTAKVIVAFVSFVEMGLLIDQLSIQNITGFQLIGVESWITTKNYFTPNSFSSLRGSLGFA
VRKINIEGFIDYVIKAFWDTAFPCTKTEGNSSQYSISCTRSEDLLELKNYTEDVLEQRYASNVY
KAVYAVAHSLHSLLGCENQGLCDKNKPIRPQQMVEALQKVNFTIQMGDQVWFDSTGGTVA
HYEVVNWQQDFDGSFQFKTVGYYDASLPPQQRLMLNTKNIIWAGGQQKKPNSVCSESCP
PGTRKAAQKGRPVCCYDCIPCAEGEISNETDSINCKQCLEEYWSNAEKNKCVLKAVEFLSFT
EIMGIILVFFSLFGVVLTTLVTVLFYRKKDTPIVKANNSELSFLLLFSLSLCFLCSLTFIGRPTEWS
CMLRHTVFGITFVLCISCVLGKTIVVLMAFKATLPGSNVMKWFGPVQQRLSVLAFTLIQIIICVV
WITLSPPFPYKNMEYYKEKIIIECNLGSTLGFWAVLGYISLLSVLCFVLAFLARKLPDNFNEAK
FITFSMLIFCAVWITFIPAYVSSPGKLTVAVEIFAILSSSFGLLFCIFAPKCYIILLKPEQNTKQHM
MGKT
>Dr_9_3_F
MLLFLYTLLLFHHLHTKAGNTLCRIMGDPQYPLLSTDGDITIGALFPVHSTETLPSFEFTKQPQ
LLSCSSVYLRDFRLAQILIFAIEEINRSVQLLPNISIGYRIYDTCGSRQSTMSAIMGLMNGQEF
GAGERCNGRSPIHAIIGESESSATVILSRTTGPFKIPVISHSASCECLSNKKDYPSFFRTMASD
YHQSRALAYLVKDLGWSWVGAVSSDNEYGNYGMSIFLKIAQEVGICVEYYAKFYRTEAENLH
KAVNMIKKGTAKVIVAFVSLPEMGILVDQLSIENITGLQMIGVKSWITVKSFITPSSFHVLKGSL
GIAVKKISVEGFAEYAINEFWKKGFPCLKSEVNSSRYALSCSRYEDLFVLKSISEDVPEQRYAS
YVYKAVYAVAHSLHSLFNCKEHEGCEKDLTIQPQQVVEALKKVKFTIKTGDHVWFDSTGAAV
AQYDVVNLQLDSYGSTLFKSVGYYDASLPPEQQFVLNTTNIIWAGGEQKVRSVCSESCPPG
TRKATQKGRPVCCYDCIQCADGEISNETDSNNCKQCPEEYWPNTEKSKCVLKIVEFLSFTEL
MGIVLVIFSLFGVTLTVLVTFLFYSKKDTPIVKANNSELSFLLLFSLTLCFLCSLTFIGRPTEWSC
MLRHTTFGITFVLCISCILGKTIVVLMAFRATIPGNNMIKWFGPVQQRLSVLAFTLIQILICVVWL
TISPPFPSKNIKYYKEKIILECSLGSTIGFWIVLGYIGLLAVLCFILAFLARTLPDNFNEAKFITFSI
LIFCAVWITFIPAYVSSPGKLTVAVEIFAILASSFGLLFCIFAPKCYIILFKPEQNTKQHIMGKT
>Dr_9_4_F

MLLFIYILLLFHQLHTKAGNTQCRIMGDSTYPLISKNGDITIGALFPIHSTETLTSLKFTRKPQPI
SCSSVNLRDFRLAEIMIFAIEEINKSESLLPNVTIGYQVYDTCGSRLSTMSAIMGLMNSQGFD
AEDGCKGQTPIQAIIGDSESSATVILTRTTGPFKIPVISHSASCECLSNRNDYPSFFRTTASDYH
QSRALVHIVKHLGWTWLGAVSSDNDYGNYGMSIFQKIAQEEGICMEYSVKFYRTEQEKLQK
VVETMKKGTAKVIVAFVSFLEMGLLIDQLSVQNITGLQMIGVKSWITSENYITPKSFHVLGGSL
GFAMRKMYIEGFSDFALKTFWEKHFQCSQTKPNASNYASSCSRYNDLLMLKNYKEDVTEH
RYSSNVYEAVYAVAYSLHSLLKCKERVKCEKGMTIQPQQVVEALKKVNFSVKFGDRVWFDST
GSTVAQYEVVNWQRISDESFQFKTVGYYDASLPPNQRFLLNTENIIWAGGMLEKPRSVCSE
SCPPGTRKASQKGRPACCYDCIPCAEGEISTDTDSNNCKQCPGEYWSNDKKNKCVLKAVE
FLSFTEVMGVLLLFVSLFGVGLTTLVAILFYNKKDTPIVRANNSELSFLLLFSLILCFLCSITFIGR
PTEWSCMLRHTAFGITFVLCISCVLGKTIVVLMAFKATHPGSNVMKWFGPVQQRLSVLVFTFI
QVIICVLWLTISPPFPYRNMKYYKEKIILECSLGSIVGFWAVLGYIGLLAALCFILAFLARKLPDN
FNEAKFITFSMLIFCAVWITFIPAYFSSPGKLTVAVEIFAILASSIGLLFCIFAPKCYIILCKPEQNTK
QHIMGKN
>Dr_9_5_F
MLILLSIFLLFNEPFTKAEDTICQKMGDPKYALFSKVGDVTIGGIFSIRSVEILPSLEFTQKPQLL
SCSSVNLRDFRMAQIMIFAIEEINRSESLLPNVSIGYKIYDSCGSRLSSISATMSLMNDQEFPK
GNICNGQYPIHAIIGETESSATVILSRTTGPFKIPVISHSATCECLSNRRDYPSFFRTIASDYHQ
SRALAYIVKYFGWSWVGAVNSDNDYGNNGMAIFLKTAQEEGICVEYSVKFYRTEPEKLQKVV
ETMKRSTTKIIIAFLSRVEMVNLLEELSIQNISGFQVIGVEAWTTAKSLITPNSFHILGGSLGFAV
RKIDTEGFLDYVIKSFWDSAFPCLIVNSSQLKVNCSSYKDLLVLKNDNEDVPEQRYASNVYKA
VYAVAHSLHSLLNCKEKKGCQKNLTIQPYQMVEALKKVNFTIKFGDRVWFDSTGATIAQYEV
VNWQQDSDGLIQFKPVGYYDASLPPDKRFVVKTENIIWAGGKLKKPKSVCSESCPPGTRKA
AQKGRPVCCYDCIPCAEGEISNETDSVNCKQCPLEYWSNTEKNKCVFKSVEFLSFTEVMGV
VLVFFSLFGVGLTMLVAILFYNKKDTPIVKANNSELSFLLLFSLTLCFLCSLTFIGRPTEWSCML
RHTTFGITFVLCISCVLGKTIVVLMAFKATLPGSNIMKWFGPVQQRLSVLAFTLIQVIICVLWLII
SPPFPYKNMKYYKEKIILECSLGATICFSAVLFYIGLLAILCFILAFLARKLPDNFNEAKFITFSM
LIFCAVWITFIPAYVSSPGKFTVAVEIFAILASSFGLLFCIFVPKCYIILFKNEQNTKQHIMGKI
>Dr_10_1_F
MFGFGFLLLCSIKAKEVESNCNMIGKPENPLLSQDGDIIIGGAFSIHNKINLIIPSFTEKPHHLM
CTSLNLRELHFAQTMIFAIEEINSKRSLLPNISIGYQIFDSCGSTLASMRSSMALINGQELTAEH
TCSGKPAVKAIIGESESSTTIVLSRAAGPFNIPVISHFATCACLSSRKQFPSFFRTIPSDYYQSR
ALAQLVKHFGWTWIGAVRSDNDYGNNGMATFVEVAEKEGVCIEYSEAISRTNSKDKIAKVVE
VIKKGTAKVLMAFLAQGEMDVLLEELIRQNVVGLQWVGSESWITSSYLATERTLNILGGAIGF
TIIKSKIPGLKEFLLKVGPSQNLSNALLGEFWEMVFGCCLSPTVCPNSEHATFCDGSENLTNV

SNAFTDVSELRISNNVYKAVYAIAYALHNTITCKNSNGGNENITCGDVDLLVSSQVLHSLQNV
NFTMDSGETVYFDKNGDPMAKYELVNWQKNGAGETKFITVGQYDASLSSEQQFVINSFDII
WAGDSPTKPISVCTESCQPGFRQAVIKGRPVCCFECLQCPAGEISNTTDSAECIKCPLEYWS
NKNHSICVLKKVEFLSFEENMGILLTAFSLTGVTLTIAVAIVFYKFIDTPLVKASNTELSFLLLFSL
SLCFLCSLTFIGRPTEGSCMLRHSSFGVTFALCMSCVLTRTIAVVMAFKTTVPGSGLPHCSLP
LQRISVFCCTVFQVMICILWLALARPMPYKNSMYSLDKVILECDLGSAIGFWAVLGYIGLLSVL
CFFLAFLARKLPDNFNEAKFITFSMVIFCAVWITFIPAYISSPGKLTVAVEIFAILASTFGLLFCIFT
PKCYIIIFKPEQNTRKHIMGKT
>Dr_7_1_F
MLLLATILTTIACTLSAAEPECEAYMTDELLYFSKEGNVSIGGVFSFHQNPVGVNPTLRTNPGN
IRCNGLDPGELQYAITMIFAIEEINNRTDLLPGFILGYRIYDSCPSIPLSVGASLTLMNGQMETK
KSCASPSAVQAVIGETTSTSTIDIARTIGPFKIPVVSHHSATCACLSNRQQYPSFFRTIPSDYYQ
SRALVKLVTYFGWKWVGAVRSMGDYGNNGMATFLEAAEKEGICVEYSVSIYRTNSREKILEV
TDIIKKSTSKVIVAFADGNDLDMLIKELYYQNVTGYQWVGSEGWITYRFLATAINYAVVGGAIGF
AVPNAYIPGLKEFITGSQPSLRPGNTGLVELWESVFDCTLNSQTHNASKICNGQESLANINTR
FTDVSDASLLNNVYNAVYAVAHAVEELLTCEKGKGPFHKKTCAEKGKIQPWQVLYYLTQVNF
TTKNGENVHFDKHGDPVARYTLVNWQMSYEGIITFESIGLYDASKPEGQEIQMRDDIEAIWA
GNQKKVPLSVCSETCLPGTRQAFVKGKPICCFDCIDCADGEFSNTTNAVTCIPCPLEYKSNG
NRTQCVLKNIEFLTFNEVMGNILVTFSMCGGCLTITVGLIFFYHRHTPIVRANNSELSFLLLFSL
TLCFLCSLTFIGQPTEWSCMLRHTAFGITFVLCISCVLGKTLVVLMAFRATLPGSNVMKWFGP
PQQRLSVFVFTFIQLLICMLWLTMSPPFPNKTTNNYKDKIILECDSGSAVGFWAVLSYIGFLAI
LCFILAFLARKLPDNFNEAKFITFSMLIFCAVWITFIPAYISSPGKFTVAVEIFAILASSYGMLFCIF
IPKCYIILLKPDLNSKKKIMGKV
>Dr_11_1_F
MQLWIVGLIGEIIRLCSTEQSCGLQGRVISESLYKEGDVIIGGLFPVYNEAPVPNHAFTQIQNR
SRCQGVDLRSYRWLKTMLFTVEEINRDPFLLPNIILGYLVADTCLAESTTLSAALAIMTGQEET
VSDTECIVAPRVPVIVGDARSSASIGVADTLGVFDIPMVSYFASCACLGDNHRFHTFLRTVPS
DAFQAKAMARLLHLLDWTWVGVVAGDDEYGKSGVQLLLKELEHVGVCVDYLEFIPKSHSQ
RRIRRTVETIQSSTAHVVVTFAIAPDIEVLFKEVVVQNVTNRQWIATEAWSTSVQFLDPASIPLLA
GTLGFALHRADIEGLGAYLSQLNPAKQSNEPFVKDVWEEIFGCSLAQDWQPSFKRPKCTGS
ENVEIHGGIYTDVSQLRVTYNVYKAVYAIAYAIHNMIACQPGRGPFENGQCPDVNQIKPRQLL
HYLNAVNFTTPVGELVYFEDNGEPSASYDIMNWHVDESGAVNFVQVGQFDAANGPGQELNI
NIKKVVWGGGWSDQVPVSVCSVSCLPGTRKSVQKGKAICCFDCLPCAAGEISNLTDSTKCI
RCPEKFWSNVERTKCVPKIVEFLSLQDTMGIVLTVLSVTGATLTTTVLATFFHHRDTPLVRANN
SELSFMLLVSLTLCFLCALVFIGRPATWNCMLRHTLFGVSFVICIACILSKTVVVLVAFQATLPG

SNLMQYFGPIQQRAGILVCTMVQVVICLLWLLLAPPLPTERAGGEFGARVILQCTVGSVVGFA
LVLGYIGLLAVVCFLLAFFARKLPDSFNEAKFITFSMLIFCTVWIVFVPAYVSSPGKYTVAVEIFAI
LASSYGLLLCIFTPKCYIILLKPEKNTKKNMMAK
>Dr_1_1_F
TPVLRAIHLVFFLFYPIQSNSEANCKLWKELDLTVVHKEGDVILAGMFPIHSKGIDQELNFRNQ
PDQRKCWGFNMRVFRWSQAMIFFIEEINRNPTLLPNITLGYRLYDTCGLIALSLRTALSVVSQ
PMKRSSSEMCSSPSIPIIIGDSGSTLSMAISRLLNLFHIPLVSYFASCACLSNKHQFPYFFRTIP
SDVNQANALARLVKHFGWTWVGTVGADDAYGRTGIDLFTAAVTQLGVCVSYRIIIPKLPTQQ
QLQDIVRTIRDSSAHVLVAFAIEEDIKPVVDEIVLQNVTGKQWVASEAWVTSTLISTKENFPSLS
GTIGFAIRRAEIPGLKHFLDSIQPLADPYNVFAREFWETQFQCTLNTSLPTSSTMDPVNYSHS
CTGMERMQDTQSIFNDVSQLRVTYNMYKAVYTVAHALHNLLLCQREHRSALTQQCPDIHNL
QPWQVIEVLRKVNYTNMFGDLIYFDENGDPVGSYDIVNWQKGGDDVPVQYITVGRFDSSLP
KGQQLVLNQNKIVWHGGTNKVPVSVCSASCPPGYRKVRLEGQPVCCYDCMLCAEGSISNT
TDQAECLLCPEDFWSNKHRNYCVPKEIEFLSYLEAFGMVLAAIAILGAVAAISVGVVFFRHRD
TPLVRANNSELSFLLLISLTLCFICALTFLGQPSHWACPLRRISFGLTFALCLSCLLSKTLVVLIA
FKSTLPGNNTARWFRPPQQRLGVFICSLLQGGVCIAWLTTASPYPVKNTWLYRDRIILECHLG
SVVYFCCVLGYIGCLAAFCFILAFLARKLPDNFNEAKFITFSMLIFCAVWITFIPAYVSSPGKFTV
AVEIFAILASSYGVLLCIFAPKCYIIIFMPEKNSKKYLMTQK
>Dr_12_3_YP
VQVVICCTWLCVAPPIPHMLMTHETARIILLCDVGSSFAFSLVLGYIGLLAAVCFLLAFFARKLP
DNFNEAKFITFSMLIFCAVWIAFVPAYISSPGKYTVAVEIFAILASSYGLLLCIFTPKCYIILLKPEK
NTKRYVMSKS
>Dr_9_1_F
MSVFFCTLLIFFQLYAKAEKPICIMMGDPKYPLLSKEGDISIGAVFPVHSIETLPLFKFTQKPQL
LSCSSVSIRDFRMAQIMAFAIEEINRNESLLPNVSLGYQIYDTCGSGLSLMGANMALMNVQE
FASRGSCNGQSPVHAIIGETESSNTVILSRTTGPFKIPVISPSASCECLSNRKEYPYFFRTIASD
YHQSRALAYIIKYFGWSWVGAVNSDNDYGNHGMAIFLSTAEKEGVCVEYSVKFQRTEPEKL
KKVVDTIKKGTSKVIVAFLTEFEMKNLLEYLIIQNVTGLQVIGVEAWITANSMIKPNSFHVLGGS
LGFAVKKLNIEGFEDYVTKAFWETAFPCSQTSQKENSQYKLICNIYRDLLVLKNDNKDVPEQR
YASNVYKAVYAVAHSLHNILKCRENGACETDMKIQPQQVVEALRKVNFTIKMGDCVWFDST
GAVVAQYEVVNWQPDYNGSIQFKPVGYYDASLPPNQRFVINTENIIWAGGQLKKPRSVCSE
NCPPGTRKAVQKGRPVCCYDCIPCAEGEISNETDSNNCKQCPGEYWSNAEKNKCVLKAVE
FLSFKELMGIVLVFFSLLGAGLTTLVAILFYSKKDTPIVKANNSELSFLLLFSLMLCFLCSLTFIG
RPTEWSCMLRHTAFGITFVLCISCVLGKTIVVLMAFKATLPGSDVMKWFGPIQQRLSVLVITIV
QVLICVLWLAVSPPLPYKNTKYFKEKIILECSLGSTIGFSAALGYIGLLAILCFILAFLARTLPDKF

NEAKFITFSMIIFCAVWITFIPSYLSSPGKLTVAVEIFAILASSFGLLFCIFLPKCYIIMCKPEQNTK
LHIIGKL
>Dr_12_1_F
MAFLRLYLFLTVLLFSFFISSQLPQICRLLRRTDDLPVLVSEGDIMIGALFPLHDTILESPPSFTT
EPHPTQCSGFNFRTFRWMQTLIFAIEEINRDKQLLGNLTLGYKIYDSCSTHFHALRTALTLMN
GNEEIAGISECRGGVPVVIGDGGSTLSIVVAQFLGVFHVPQISYFSSCACLSNKLDFPAFLRTI
PSDFFQVDALVQLVQHFGWSWIGTFAGDDAYGRGGAQIFNEKVTKLGACIAFYEIIPKNHQQ
TEMSRIVERILESRSHVILVFALEQDARALFLEVLHHNLTGIQWLASEAWITAAILSTPEFHSVLQ
GSLGYAIRRADIPGLQPFLLRLHPSKYPQDPFVEQFWEEMFKCSLGIINRSSSIRPPCDGSEV
LVNINNIYSDVSQLRISYNVYKAVYAIAHALDLMLHCEPGKGPFAGGLCPNFSSMHPWQLLD
YLKHVHFTNDFGEDTKFDINGDPVAMYDLINWQLSGKQEMQYVTVGKYDETMKPKLVIEEK
NIIWSGNQKQVPLSVCSTSCPPGTRQATRPNFPICCFDCIVCAAGEISNHTDALECVKCSPEF
WSNTRRDTCIPKVVEFLSYNDTMGITLLSVALLGSCFTLAVTLIFALKRHTPLVRANNSEISFLI
LSSLLLCFLCALAFIGRPTTWSCGLRHTAFGIAFSLCLSCVLGKTLVVLMAFKASLPGSNVMR
WFGPLQQRGIIFMCTAVQVVICCTWLCVAPPIPHMLMTHETARIILLCDVGSSFAFSLVLGYIG
LLAAVCFLLAFFARKLPDNFNEAKFITFSMLIFCAVWIAFVPAYISSPGKYTVAVEIFAILASSYG
LLLCIFTPKCYIILLKPEKNTKRYVMSKS
>Dr_13_1_F
MAQIYATLLTLCLLLYIRVQCQSNNKYKCIYQGDDDTYSFYQGGDLVLGGIFPLHSSTIPQFFS
FTTKPKLIQYKFFTPRALRWMQTMIFAVREINQRQDLLPNLSLGYHIRDSGDDIPVSVKRSLLL
VNGQPEKGSGQSCEDTRKQPSPVIVGEASSGVSMAVLRTLGTFQIPLVSYFASCSCLSNKR
EFPAFMRTMPSDLFQIKALVKLVYYFKWTWLGVIGVDTDYARFAIQLFLKESEKYNICPAYVHI
YPVALTQNTVEELVKILKSSSARVILNFSVDSYLYGVLKECRRQNVTNLQWIASEAWATSKVL
WDDFGDLLKGTLGFAIRRADIPHLGSYLRSVSSSVAQTSPFFTEFWEETFHCRLNGSLNTHV
HEEASYNWPACNGNETLDDVYTLYSDVSQLRVSYNVYKAVYLIAHALHDMSTCDPGKGPFK
NGTCGSLYQILPWQLLYYMKRTNFTTLGEEVRFDKNGDPIASYDLMNWQRESDGSLQLVRV
GIYDASFKDDKDLVIDESVIMWHRGDKAPESLCSKSCLPGSRKARQKGKPVCCFDCISCAE
GEISNQTDSIDCLTCSKDTWPNQSQDQCIPKTLEFLSFQEPLGIILWVFSALGACAALAVLCV
FVMYRTTPVIRGNNIELSFLLLLFLCACFLIGLTFLGKPTDWLCQIRYPAFGISFTLCISCILAKT
VVVLMAFRATIPGNNVMKWFGPVKQRSSVILCTCVQALICIIWLTTKPPLASYNSKFLSATIIVE
CSVGSEVGFWCVLGYIGFLSSLCFFLAFLARKLPDNFNEAKFITFSMLIFFAVWITFIPVYVSSS
GKYMVAVHVFAILASAFGLLMCIFAPKCYVVLLKPERNDKKSMMKK
>Dr_10_2_YP
KSELLLLMVLVTHGICVPNLAQVCRLLGQPALPLLSEERNINIGAIFSLHKSALLKSHNFTSKP
KQTTCGGLNLREFKFVQTLIFAIEEINNSSLLPGASLGYKIYDSCGSVAQAIFSGMALMNGYE

ETLSDTSCSTPPAVHAIVGESNSSPTIGLASVVGPFSLPLVSHFATCSCLSNRKRFRTFFRTIP
SDYYQSRALAQLVKHFGWTWVGTVRSRSDYGNNGIATFEEAAKQEGICIEYSEAVFKTDPEV
QFLKTIEVIKKATTKVVVAFMAFGDFVLLLKVMAQQNITGIQWIGSESWITSRNLAETKEYHFL
SGAVGFAIANSKPVGLREFLMNVHPDKELNNELLKEFWETVFLCSFRNSSSGGCTGSEKLS
ELQNEYTDFSELRIENKVYTAVYAVAHSLHNVLKGFKFFTNSSKKQLPTPQKVLEYLRDVNFT
VNTNENIFFDASGDPVARYDLVNWQPTKDGSLQFKLVGIYDSSLPSEQRLQINQESMLWAG
NSGQLPVSVCSESCPPGTRKAVQKGRPICCYDCIPCAEGEISNYTDSSDCFPCGLEYWSNE
SKDRCVLKVIEFLSYTEI
>Dr_13_2_YP
TLLMVWFLMYLSVQSQYNSKVRCVFQGDDDTNTFYQGGDVVLGGLFPLHFSPISSLSSYKT
KPTPTTYKFFTPRALRWMQTMIFAVREINQRQDLLPNLSLGYHIRDSGDDIPVSVKRSLLLVN
GQPDKDSGQSCEDTRKQPSPVVVGEASSGISMAVLRSLGCFKIPLVSYFASCSCLSNKKEF
PAFMRTMPSDLFQIKALAKLVHYFQWTWVGVIGVDTDYARFAIQLFLKESQQLNICLAYVQLY
PVALNQDSAEELVRMLKSSSATVIISFSVDSYLYGILKECRRQNVTHLQWIASEAWATSKSLW
EEFGDLLKGTLGFAIHRADIPHLGSYLKEIRPQTSHFLSEFWEETFRCRLNGSLNTHAHGEQA
QNWPACNGSESLDDVYTTYSDVSQLRVSYNVYKAVYLIAHALHDMSTCVPGKGPFQNGTC
GSLFQIQPWQLLYYMKQTNFTTLGEEIRFDENGDPIASYDLMNWQRGSDSSLQLVRVGFYD
ASLEDDKDLVIDESLIMWHRAEKAPESLCSKSCLPGSRKARQKGKPVCCFDCISCAEGEISN
HTDSIECLTCSEDTWPNQSQDQCIPKTLEFLSFQEPLGIILWVFSAFGACAALAVLCVFVMYR
KTPVVRGNNIELSFLLLLFLCACFLIGLTFLGKPTDWLCQIRYPAFG
>Dr_2_1_F
NTMIHIFLCIFHLSSAAMTSRCIPRGNFDLPVFMTSGDFTIGGIFPLHYRVELPPTDYMKKPLT
AMCRGFDPMAFHWALTMGLAVEEINNRKDLLPEHTLAYRIFDSCATPVMAQKAVLAALNGQ
DVVQSFMCSGASPLLGLIGESGSSQSIVVSRTLEPFRIPMISYFSTCSCLSDRKQFPTFFRVV
PSDDYQVKAIAQLLKRFDWTWIGVVTEDHDYGRFALQGLKREIENTKICLAYHEMIPKDYTQE
RVLKILKVMKESTAKVVVVFSGEGEFYPFLTEFVAQNITGIQWIASEAWVTASMLAETYSFLDG
TIGFAIRQGHVPGLQDYIRTVTPERYPSIPQVQELWEALYGCSPSTSTLSSHLPSCTGKEILRK
EYSAYMNTSSPRVTYNVYKAVYAFAHSLHNLIECRNGHGPFENLSCANLNNVFPWQLQHYL
QEISFSISGEEVNFDIKGDAIPSYDLINWQRSASGDMQFIKVGLYDGAQHSGKELVIEKQAIAW
SNQQTKAR
PVSVCSNGCTPGYRKAVRHGQSLCCFDCVPCDIGKISNQTDSVDCLSCPEDYWSNVNRTE
CIQKVIEFLSHDAMGMTLTVIAVAGAFLTITVLGIFLYNKGTPIVRVNNSELSFFILVSLTFCFLCA
LIFIGEPTSWSCMLRHTAFSITFSLCISCILGKTLVVLAAFTATRPGNNIMKWLGPTQQRIIIFCC
TLVQVLICTVWLVVSPPFPYRNTKYQQSKIILDCSVGSDLAFWCVLGYIGLLACVCFFLAFLAR
KLPGNFNEAKYITFSMIIFCTVWLAFVPAYVSSPGKFTTAVEIFAILASSFGLLFCLFAPKCYIILV

KPEKNTKQHLIGKV

>Dr_2_3_YP

RAMIYIFMSIFHLSSASMALICSPHDFDLPVFMTSGDFTIGGIFPLHYRVKLPPTDYMKKPLTA
QCRGFDPRAFRWALSMKLAVEEINNRKDLLNNFTLAYKIFDSCSTPVTAQKAVLAVMNGQEV
VQSSMCSGAGPLIGLIGESGSSQSIVLSRTVQAFQIPMISYFSTCSCLSDRKQFPTFFRVVPS
DDYQVKAIAQLLKRFDWTWIGVVTEDHDYGRFALQGLKREIENTNICLAYHEMIPKDYTQERV
LKILKVMKESTAKVVVVFSVEGEFYPVLREFVAQNITGIQWIASEAWVTASMLAETYSFLDGTI
GFAIRQGHVPGLQDYIRTVTPEKYPSIPQVQELWEALYGCSPSTSTLSSHLPSCTGKETLRKE
YSAYMNTSSPRVTYNVYKAVYAFAHSLHNLIECRNGHGPFENFSCANLNNVFPWQLKHYLE
DVSFSISGQNVNFDNKGDSVPYYDLINWQRSASGDMQFVKVGLYDGAQHSGKELVIEEQAI
TWSNQQTKAR

PVSVCSYRCAPGFRTAACRGQPLCCFDCVPCESGKISNQTG

>Dr_2_2_YP

TMTILLLWLLVYHLSVLNPAQASKCLLQNEFEPGLMANGDFVIGGIFPLHYNQEMPDLNFTY
KPGPVKCNGFDTRAFRWAITMKLAVEEINKRVDLLPNYTLGYKIFDSCAYPLTGQRSAVAVLN
GPNELESPLCADAAPLLAVIGESGSAQSIVVSRILQPFGIPMISYFSSCACLSDRREFPTFFRVI
PSDAYQVKAIAKLLRHFNWTWIGVIRGDHEYGRFALQGLLKELEGTGICVAYQKMIPLLYDRQ
KALEIIHVMSHSTARVVVVFSAEGELTPFLRDYMEQNVTGIQWIASEAWVTSSVFAGSEFDPF
LGGTIGFGIRQGQIPRLKEYLTTVNPERYPTNPLVYELWGALYGCSPSWSNLSSHLPSCTGK
ETVRLQYSAYLNTSSPRISYNVYKAAYAIAYSLHNLIYCTPGKGPFSNSTCANAPHIYPWQLQ
QYLQEVSFTISGEKVNFDMKGDSIPSYDLINWQRGSAGNIEFINVGMFDGALESGQELVIKEE
AIMWPGHQTEA

VLVSVCSNSCAPGFRKAVRRGQPLCCFDCVPCDSGKISNETDSLDCIACSEDYWSNADGTV
CIPKVVEFLSHDAMGLTLTVIAVAGACLTLAVFSVFLYYKNTPVVRINNSELSFFILLSLTLCFLC
ALIFIGEPTSWSCMLRHTAFSITFSLCISCILGKTLVVLAAFTATRPGNNLMKWLGPTQQRIIIFC
CTLIQVLICTVWLVASPPFPYRNTKYQQSKIILDCSVGSDLAFWCVLGY

>Dr_4_1_F

MHVNLLLILTLLCIRRLFPAVCGVHLGTCILQGDPQPPSLFSKGDFVIGGTFTIHYYLRTEKRTY
TVRPQPLMCSGSMDFRELRFARVLQFAIQEINNSSNLLPGITLGYHIYDSCASVPMVIKVAVQ
LANGLQLAFNDTDSCAQSSEVLALVGESGSTAAITTSRLFGPFGIPQVSHYATCACLSDKRQ
HPTFFRTIPSDHHQAAALARMVKRFGWTWIGAVRSDSDYGNNGMASFLKAAEVEGICVEYS
EAYYRTQTRNKLQRVADVIRRSTARVIVAFMAAGDMRFLLEELSQQPPPPMQWIGSEAWVT
DPQMLRFNLSIGAVGFAIPRSVIPGFRKFLLDLSSEQALKIPVLKEFWESSFGCSLKQHTGYF
SGMPACDGTEDLGTLKNPYTDTSQLRISNMVYKATYAIAHALHGIVCNGKLCDKNIKVEPRK
VSDQLKQVNFSKNNYSVSFDANGDPVAVYELVNWQLQGDGSIDFVTVGKYDASQPKGQEF

SLNRAIIWYDGTEKVPVSVCSESCPPGTRKAVKKGRPVCCYDCINCADGEINNETDSLDCHK
CQPDYWPNAEKIKCLPKPVEFLSWDEILGNTLAAFSIAGSLVALSMALVFYKNRVSPIVRANN
SELSFLLLFSLTLCFLCSLTFIGQPTEWSCMLRHTAFGITFVLCISCVLGKTIVVLMAFKATLPG
SNVMKWFGPPQQRLSVFGFTLVQIIICVLWLTISPPFPYKNMQHYKDKIILECSLGSAVGFWA
VLGYIGLLAFLCFVLAFLARKLPDNFNEAKFITFSMLIFCAVWITFVPAYVSSPGKFTVAVEIFAIL
ASSFSLILLIFAPKCFVIVFRPEENTKRHLLGKV
>Dr_4_2_F
MNAHLLFTVKMLCIARLCIAVCGVDLGTCILQGDAQPPALSEDGDFIVGGAFTIHYYVRTEKY
TYTRRPQPLECSGSMDFRELRFARALQFAIQEINNSSDLLPGIFLGYHIYDSCGSVPMAIKVA
LQLANGLDPKYNDTDSCAKSAAVLALVGDSASTPAISISRIFGPFEIPQVSHYATCACLSDKRQ
HPTFFRTIPSDHHQAAALARMVKRFGWTWIGAVRSDSDYGNNGMASFLKAAEKEGICVEYS
EAYYRTYPLSKLKRVADVIRRSTARVIVAFVAAGDMRFLLEELSKEPLPPMQWIGSEAWVSDP
QMLRFNLSIGTMGVAVPRSLIPGFRKFLLDLSPYKVLKFPLLTEFWESSFSCSLQQQTDPST
GMPACDGTEDLGRLKNPYTDTSQLRVSNMVYKATYAIAHALHGIVCNETQCSKNIKVKPRQ
VFDQLKQVNFSKNNYSVSFDANGDPVAVYELVNWQLQGDGSIDFVTVGKYDASQPEGQEF
SLNKAIIWYDGSEKVPVSVCSENCPPGTRKAVKKGRPVCCYDCIPCGEGEISNKTDSPDCD
KCPPDYWPSTEKDKCLPKPVEFLSWDEILGIILAVLSVSGSLLALSMALVFYKNRASPIVRAN
NSELSFLLLFSLTLCFLCSLTFIGQPTEWSCMLRHTAFGITFVLCISCVLGKTIVVLMAFKATLP
GSNVMKWFGPPQQRLSVFGFTLVQIIICVLWLTISPPFPYKNMQHYKDKIILECSLGSAVGFW
VVLGYIGLLASLCFVLAFLARKLPDNFNEAKFITFSMLIFSAVWITFIPAYVSSPGKFTVAVEIFAI
LASSFSLILCIFAPKFFIILFRPEENTKKHLMGKV
>Dr_4_3_F
LLIVTLQCIITRLYVVYGNRLGSCILQGDPQPPVLFDEGDFIIGGAFSIHYYLRTEKHTYTMRPQ
SLECSGSMDFRELRFARVLQFAIQEINNSSDLLPGITLGYRIYDSCGSVPMAVKLSFQLANGL
DLIFNDNDSCSKSAAVAALVGESGSTPSISISRLYGPFGIPQVYHYATCACLSDKRQHPTFFRT
IPSDHHQAAALARMVKRFGWTWIGAVRSDSDYGNNGMASFLKAAEEEGICVEYSEAYYRTQ
PRSKLMRVADVIRRSTARVIVAFMASGDMRLLLEELSQQPLPPMQWIGSEAWFTDPEMLRF
NMCIGGVGFAIPRSVIPGFRTFLLDLSPQRAMKFPLLTEFWESSFSCSLKQQTGPSTGMPAC
DGTEDLGRLKNPYTDTSQLRISNMVYKATYAIAHALHGIICNEKKCNKNIKIEPREVLDKLKQV
KFSKNNYSVSFDAYGDPVAVYELVNWQLQKDGSIDFVTVGKYDSSQPKGKEFSLSRAIIWYD
GTEEVPVSVCSESCPPGTRKAVQKGRPVCCYDCINCADGEISNDTGAL
DCLDCHECLSDYWPNNEKNKCLRKPVEFLSWDEILGIILAAFSVAGSLVALSMALVFYKNRA
SPIVRANNSELSFLLLFSLTLCFLCSLTFIGQPTEWSCMLRHTAFGITFVLCISCVLGKTIVVLM
AFKATLPGSNVMKWFGPSQQRLSVFGFTLVQIIICVLWLTISPPFPYKNMQHYKDKIILECSLG
SAVGFWAVLGYIGLLAFLCFVLAFLARKLPDNFNEAKFITFSMLIFCAVWITFVPAYVSSPGKFT

VAVEIFAILASSFGLILCIFAPKFFIIIFRPERNTKKHIMGKV

>Dr_14_1_F

MPVIELVSLGLALLLNFSVVLCTSLDKSCWTLGDFNSPVLEQDGDIVIGGLFPMHNIAPETDY
NFSDLPHYQDCSRFDFRAFRWVQTMVFAIEEINSNSSLLPGVALGYRILDSCDHVHTSLRSA
LFLLNGTFGQTQTDAVGTANCLSTAPVPAVIGLASSSPTRAVAQTLGPFGIPLISYFATCTCLTD
KKEYPSFLRTVPSDRFQVQGLVQLVSHFGWRWVGTVGTDDDYSHYGIQAFTEQLERLGSCI
AFHQTIPKSPSQAQIRSILNSLESSTAQVIIAFATEGELLELLVEVARRNLTRLQWVASEAWVTA
KLLTIPELHPVLIGTVGFSFRGTAIPGLAEFLFRVRPSSRPESAFTNMFWEELFGCRLGYEDSN
DSVLPLCTGSENLLETESSYTDVSRVRISYNVYKAVYAIAHALHQLLQCGSKEQGSDHSCNT
ESKFSPLQLLRYLKKVHFTNQFNEKVYFDTNGEPVPLYDIINWQKNARGTINFQLVGTYDGSA
PHGQQLKIEEGLIRWTGGQTKVPVSLCSPPCPPGTRQATRPGQPVCCFDCLPCAEGEISNIS
GATECLKCPEYYWPNKERVSCVAGIEEFLSYHDVMGIILISLSLFGVAVATVVMVIFFIFRSTPIV
KANNSEMSFLLLLSLKLCFLCSLVFVGRPSPLTCRARQAAFGISFVLCISCILVKTIVVLLAFRS
TVPGSISLKVFGPPQQRVFIFCCTTGQVILCACWLALAPPYPYKNTSYQDGRIILECKDILPLG
FYLVLGYIGLLSCMCFVLAFLGRKLPDTFNEAKLITFSMLIFCAVWISFIPAYNSSPGKYTVAVEI
FAILASTFGLLFSIFIPKCYVILLRPDLNTKKGMTGKL

>Dr_16_1_F

MDTWITLYLCFYLFFKSISASSISKSGSCQLQGHFKLNGMYQDGDLMIGGLFAFHLITVFPEL
NFKREPEQTHCERFYMASFQQAQTMVYAINEINNNPNLLPNITLGYHLYDNCVKLGVAFRSA
TALVSGTEESFNVLNCTGPPPIIGIVGDPGSTHCIAISSVLGLFRIPMVSYYATCSCLSDRSKYP
SFFRTIPSDAFQVRAMVLILKHFGWTWVGLLYSDDDYGINAAQSFQKEVQLFGGCVSFSEIL
PLDNNHMDIQRIVQVIQASTAIVVVVFSTEAYLLSLMDEVVLQNVTGRQWIASEAWATSSVFH
TKRLLPFLGGTLGIAIRRGEIQGLRDFLLSLHPDSNLRNNMVKIFWENMFECSFDTVGRKGE
TMCTGQEDIRTTNTPYSDVSELRASYNVYKAVYALAHALHDLIQCEEGKGPFRCDGITNLKP
WQLVHYLQKVNFTTGFGDHVSFDENGDALAIYDVMNWHPSSDGSIVVRTVGVVDEGASTG
KVLTLEEDEIYWNTAKNKPARSVCSESCPTGTRRTRRKGLPVCCFDCLPCADGAISTIPDSIE
CVVCPDEFWSSPKKNQCVPKDVEFLTYGDPLGISLTTASLLGSCICSAVVVIFAHHRHTPVVR
ANNSELSFLLLVSLKLCFLCVLLFIGQPQLWTCRLRHAVFGISFVLCISSILVKTMVVIAVFKSS
RPEGKSAMKWFGSHQQRCTVLVLTALQVVICAVWLTNASPKPYKNNQYTSSKIVYECTIGSV
VGFAMLLGYIGILAAVSFLLAFLARNLPDHFNEAKFITFSMLIFCAVWIAFVPAYVSSPGKYAVA
VEIFAILASSFGLLAAIFAPKCYIIILHPERNTKKAIMGRA

>Dr_16_2_F

MWITLNICLYMYFNHISGVFTSESCQLQGCFKLNGMYQDGEFIIGGLFEVQNLKVFPELSFTN
EPELPQCEEFYMASFQQALTMVFAINEINSNSKLLPNITLGYQIYDNCLRLGVAFRAAMSLVS
GTEESSNLSCTGPPPVIGIVGDPGSTQSITISSVLGLYRVPMVSYYATCSCLSDRKKYPSFFRT

IPSDAFQVRAMVQILRHFGWTWVGLLYSNDDYGIYAAQSFQQEMQILGGCVAFSEMLPYDN
NGRDIQRIAGVIQKSTAKVVVAFSTDLASLMDELLLQNVTGKQWIASEAWTTTPVLQTPQYLP
LLGGTLGIAIRRGQIQGLYEFLKNLRPDKNPKNSTIRIFWETMFGCKFEVGGKKVDEQQERG
RNKCSGQEDLNNSETAYTDVSELRASYNVYKAVYALAHALHDLVKCEEGKGPFTGNSCADIS
NLKPWQLVHYIQNVKFTTGFGDHVSFDENGDALAIYDVMNWHPRSDGSIIVHVVGVYEEAIL
GKVLTLDEDALFWNFETKKAIPPDSVCSESCPPGTRRARKKGLPVCCFDCLPCGDGEISNTT
DSPECFICPEDFWSSPENDRCVPKEMEFLSYDDPLGISLTTASLLGTCFCVLVLVIFAYHHNTP
VVRANNSELSFLLLVSLKFCFLCVLLFIGRPRLWTCQLRHAVFGISFVLCISSILVKTMVVIAVFK
TSRPEGKTSIKWFGSAQQRGTVIALTSAQVAICTVWLSSASPTPHKNNQYIHSKIVYECAIGS
VAGFSLLLGYIGLLAAVSFLLAFMARKLPDNFNEAKFITFSMLIFCAVWIAFVPAYASSPGKYAV
AVEIFAILASSFGLLAAIFAPKCYIIILHPERNTKKAIMGRS
>Dr_16_3_F
MCITLNICLHLSFIFISVNSDSCQIQGHFKLNRMYKDGDYIIGGLFEVQHLKVFPELSFRMEPE
QPKCEEFYMSSFQQAQTMVFAIDEINKNPNLLPNITLGYHLYDNCLKLVVAFRAATTLISGTEE
TFSNFNCTGPPPVIAIVGDPGSTHSIAVSSVLGLFHVPLISYYATCSCLSNRKKYPSFFRTIPSD
AFQVRAMVQILKYFGWTWVGVLYSDDDYGIYAAQSFQQEMQRFKGCVAFSEIVPYDNHRDI
QRIVAVIKASTARVVVAFSTDLLPLMEELLQQNVTGRQWIASEAWSTSPVLHLPRFVPLVRGT
LGIAIHRGKIKGLHEFLLHIQPDNDPTNNMVRIFWENMFGCSFEKGNGYGEKMCTAQEDLNI
TVNEYNDVSELRASYNVYKAVYALAHALHDLMQCEEGRGPFSGNSCADITKLKPWQMVPY
LQKVNFTTGFGDHVSFDENGDALAIYDVMNWHPSSDGLIVVRTVGVVDEGASAGRVLTLDE
DAIYWNFETKKPPRSVCSESCPPGTRRAMRKGLPVCCFDCLPCADGEISNMSDATECTSC
PNEFWSSPKKDQCVPKEVEFLSYEDPLGISLTTASLLGTCFCALVLAVFAHHHNTPVVRANN
SELSFLLLLSLKLCFLCVLLFIGRPQLWTCQLRHAMFGISFVLCVSSILVKTMVVIAVFKSSRPE
GKSAVKWFGAVQQRGTVLVLTAVQIVICVVWLSTASPTPHKNILYVRSKIVFECSIGSMAGFAIL
LGYIGILAAVSFLLAFLARNLPDNFNEAKFITFSMLIFSAVWIAFIPAYVSSPGKYSVAVEIFAILA
SSFGLLVAIFVPKCYIIILHPERNTKKAIMGRT
>Dr_16_4_F
MWAVLLFCLVLSCYYICVTLMASTGICQQQGHFTINGMHQDGDFVIGGLFDVQTYLKVYPEI
SFRTQPKLPNCELFYMESFQQALTMVFAISEINHNPNLLPNITLGYQIYDTCLRLRVAFQAATA
LISGTEETISDFNCKGPPPVIGLIGDPGSTHSIAISSVLGLFRMPMISYYATCSCLSDKKKYPSF
FRTIPSDTFQVRAIVQTLRHFGWTWVGLIYSNNDYGIYAAQSFHQEMQLFGHCVAFSEILPQ
DNNPRVIDHIMGVIQASTARVVVVFSASSLLIPLMNKVVLHNLTSRQWIASEAWVTAAVFRTPY
YQPFLKGTLGIAIRRGEIQGLHSFLLRLHPNSDQRNNIVRIFWETMFGCSFETGDKETFGQQ
MKKVCTGLEDLSTANTPYTDVSGLRATYNVYKAVYALAHALHDLMQCKEKRGPFSGNSCAD
ITDLKPWQLVHYLKKVNFTTSFGDSVSFDNNGDALAIYDVLNWQPSSEESIKLHNIGVVNEVA

TEMVLTLNNDEIYWNFEAQKPPQSVCSESCLPGTRRAMRKGLPVCCFDCLICGDGEISNTT
DAIKCTVCPDEFWSNLNKDQCVPKEIDFLSYEDPLGISLTTTSLLGTCFCALVMIIFTFHRNTPI
VRANNSELSFLLLLSLKLCFLCVLLFIGQPQLWTCQLRHAVFGISFVLCISSILVKTMVVIAVFK
SSRPEGSGAMKWFGTAQQRCTVLVLTALQIVICAVWLSTSSPTPYKNNQSIRSKIVYECAIGS
LAGFSLLLGYIGLLAAISFLLAFLARNLPDNFNEAKFITFSMLIFCAVWVAFVPAYMSSSGKYAV
AMEIFAILASSFGVLVAIFAPKCYIIILHPERNTKKAIMGRE
>Dr_16_5_F
MWAVLLFCLVLSCCYICVTLIASTGTCQQQGHFTINGMHQDGDFVIGGLFDVQTYLKVYPEIS
FRTQPKLPNCELFYMTSFQQALTMVFAISEINHNPNLLPNITLGYQIYDTCLRLRVAFQAATALI
SGTEETISDFNCKGPPPVIGLIGDPGSTHSIAISSVLGLFRMPMISYYATCSCLSDKKKYPSFF
RTIPSDTFQVRAIVQTLRHFGWTWVGLIYSNNDYGIYAAQSFHQEMQLFGHCVAFSEILPQD
NNPRVIDHIMGVIQASTARVVVVFSASSLLIPLMNKVVLQNLTSRQWIASEAWVTAAVFRTPYF
QPFLKGTLGIAIRRGEIQGLHSFLLRLHPNSDQRNNIVRIFWETMFGCSFETGDKETFGQQM
KKVCTGLEDLSTTNTPYTDVSGLRATYNVYKAVYALAHALHDLMQCKEKRGPFSGNSCADIT
DLKPWQLVHYLKKVNFTTSFGDSVSFDNNGDALAIYDVLNWQPSSEGSIKLHNIGVVNEVAT
GMVLTLNNDEIYWNFEAQKPPQSVCSESCLPGTSRAMRKGFPVCCFDCLICGDGEISNTTD
AIKCTVCPDEFWSNLNKDRCVPKEIDFLSYEDPLGISLTTTSLLGTCFCALVMIIFTFHRNTPIV
RANNSELSFLLLLSLKLCFLCVLLFIGQPQLWTCQLRHAVFGISFVLCISSILVKTMVVIAVFKS
SRPEGSGAMKWFGTAQQRCTVLVLTALQIVICAVWLSTSSPTPYKNNQSIRSKIVYECAIGSV
AGFSLLLGYIGLLAAISFLLAFLARNLPDNFNEAKFITFSMLIFCAVWVAFVPAYMSSSGKYAVA
MEIFAILASSFGVLVAIFAPKCYIIILHPERNTKKAIMGRE
>Dr_16_6_F
MWITLLINIHLILKCISAAVVLRAGACQLQGRFRLNGMYQDGDVILGGLFEAHFFTLFPELTFR
TEPAPPYCEIFNMESFQYAQTMAFAINEINRNPSLLPNISLGYHLYDNCVMLGMALRAAMSLV
SGIEESFLNLNCTGPPPIIGVVGDPSSTPSIAISSVLGLFRVPIVSHYATCSCLSDRKKYPSFFR
TIPSDAFQVRAMIQLISHFGWTWIGLLYSDDDYGTYAAQSFHQEMQLFGFCIAFSEPLRYDS
NPRDIQRLMEVIQASTSTVVVVFSPSTLVIPLMNEVVLQNMTGRQWIASESWATSPVFYTPRF
LPFLGGTLGIAIRRGEIEGLREFLLQLRPKNDPRNNMLKIFWENMFGCSFETGPHVKNVCTG
QEDLSTTNTPYTDVSELRAANNVYKAVYALAHALHDLMKCEEGKGPFSKNSCAEISNLKPW
QLVHYLQKVNFSTRFGDHVSFNKNGDALAIYDVMNWQPGSDRSIRIHTVGVVSEELEKGLM
LTLDEDAIYWNFETKKPPQSVCSESCPRGSRRATRKGHPVCCFDCLPCRDGEISNTTDATK
CTVCPDDFWSNLYKDQCIPKEVEFLSYNDPLGISLTTASLLGTCFCAFVMVIFVHHHNTPIVRA
NNSELSFLLLFSLKLCFLCVLLFIGRPQLWTCQLRHAVFGISFVLCISSILVKTMVVIAVFKSSR
PEGKMAMKWFGAAQQRCTILALTAIQVVICVVWLSTASPTPHKNNLYIRSIIVYECTIGSVTGF
SMLLGYIGLLAAVSFLIAFLARNLPDNFNEAKFITFSMLIFCAVWITFVPAYVSSPGKYSVAVEIF

AILASSFGLLVSIFAPKCYIILLHPERNNKKAIMGRE

>Dr_16_20_YP

QPPQSVCSESCLPGTRRVMRKGFPVCCFDCLTCGDGEISNTTGENVIE

QNCIIEI

NNLIIDRCVPKEIDFLSYEEL

SLQRTCFCALVMIIVTFHRNTPIVHANNSELSFLLLLSLKLCFLCVLLLIGQPQLWTGQLRHAV

FGISFVLCISSILVKTMVVIL

VFNSSRP

GKGAMKWFGTAQQRCTVLVLTALQIVICAVWLSTSSPTPCKNN*IVRSKIVYEC

VGSVAGFYLLLGYIGMLAAISFLLAFLARNLPDNFNEAKFNIFSMLIFCAVWNAFVSAYMSSS

GKYAVAMEIFAFLASSFGVLVAIFAPKCYMIILHPERNTKKFIMGRE

>Dr_16_7_F

MWITLLINIYLILKCISAAVVLRAGACQLQGRFRLNGMYQDGDVILGGMFEAHFFTLYPELTFR

TEPAPPYCEIFSMESFQNVQTMAFAINEINMNPNLLPNIILGYHIHDNCVTFGMALRAAMSLV

SGIEESFINLNCTGPPPIIGIVGHPSSTPSIAISSVLGLFRVPIVSHYATCSCLSDRKKYPSFFRTI

PSDAFQVRAMIQLISHFGWTWVGLLYSNDDYGTYAAQSFHQEMQLFGICIAFSEPLRYDSNP

RDIQRIMAVIQASTSRVVVVFSPSTLVIPLINEVVLQNMTGRQWIASESWATSPVFYTPHFLPFL

GGTLGIAIRRGEIEGLREFLLQLRPQNDPRNNMPKIFWENMFGCSFETGAQAKNVCTGQED

LSTTNTSYTDVSELRAANNVYKAVYALAHALHDLMKCEEGKGPFSGNSCADITNLKPWQLV

HYLQKVNFSTRFGDHVSFNKNGDALAIYDVMNWQPGSDRSIRIHTVGVVREEPEKGLMLTL

DEDAIYWNFETKKPPQSVCSESCPRGSRQATRKGHPVCCFDCLPCRDGEISNTTDPTKCTV

CPDDFWSNLYKDQCIPKEVEFLSYYDPLGISLTTASLLGTCFCAFVMVIFVHHHNTPIVRANN

SELSFLLLFSLKLCFLCVLLFIGRPQLWTCQLRHAVFGISFVLCISSILVKTMVVIAVFKSSRPEG

KKAMKWFGAAQQRCTILVLTAIQVVICAVWLSTASPTPHKNNLYIRSIIVYECTIGSVTGFSMLL

GYIGLLAAVSFLIAFLARNLPDNFNEAKFITFSMLIFCAVWITFVPAYVSSPGKYSVAVEIFAILAS

SFGLLVAIFAPKCYIILLHPERNNKKTIMGRE

>Dr_16_8_F

MWAILLFCLFLSCNFICVTLMVSSGTCQKQGHFTLNGMHQDGDFVIGGLFEIQSYVKVYPEI

SFRTQPKLPNCDLFYMTSFQQALTMVFAISEINNNPNLLPNITLGYQIYDNCLRLGVAFRAATA

LISGTEETISDLNCKGPPPVIGVIGDPGSTHSIAISSVLGLFRLPMISYYATCSCLSDKKKYPSF

FRTIPSDTFQVRAMVQTMRHFGWTWVGLIYSNNDYGIYAAQSFHKEMQLFGHCVAFSEILP

QDNNPRVIDHIIGVIQASTARVVVVFSASSLLIPLMNKVVLQNLTGRQWIASEAWVTSTVFRTP

YYQPFLKGTLGIAIRRGEIQGLHSFLLRLHPNSDQRNNIVRIFWETMFGCSFETEDKETFGQQ

IKKVCTGQEDLSITNTPYTDVSGLRAPYNVYKAVYALAHALHDLMQCKEKRGPLSEKNCADI

TDLKPWQLLSYLKKVNFTTGFGDPVSFDNNGDALAIYDVLNWQPSSEGSIKLHSIGVVNDEV

ATGMVLRLNKDEIYWNFEAQKAPRSVCSESCPPGTRKAMRKGFPVCCFDCLTCGDGEISNT
TDAIKCTVCPDEFWSNFNKDQCVPKEIEFLSYEDPLGISLTTASLLGTCFCALVMIIFAFHHNT
PIVRANNSELSFLLLLSLKLCFLCVLLFIGQPQLWTCQLRHAVFGISFVLCISSILVKTMVVIAVF
NSSRPEGKGAMQWFGAAQQRCTVLVLTVLQVVICAVWLSTSSPTPHKNNQYVRSKIVYECA
IGSVAGFSLLLGYIGLLAAISFLLAFLARNLPDNFNEAKFITFSMLIFCAVWIAFVPAYVSSPGKY
AVAVEIFAILASSFGVLVAIFAPKCYIIILHPERNTKKAIMGRE
>Dr_16_9_F
MWAILLFCLFLSCNYICVTLMVSSGTCQQQGHFTLNGMHQDGDFVIGGLFEIQRYLKVYPEI
SFRTQPNLPNCELFYMTSFQQALTMVFAISEINSNPNLLPNITLGYQIYDNCLRLGVAFRAATA
LISGTEETISDLNCKGPPPVIGLIGDPGSTHSIAISSVLGLFRLPMISYYATCSCLSDKKKYPSF
FRTIPSDTFQVRAMVQTLRHFGWTWVGLIYSNNDYGIYAAQSFHQEMQLFGHCVAFSEILPQ
DNNPRVIDHIMGVIQASTARVVVVFSASSVLIPLMNEVVLQNLTGRQWIASEAWVTSAVFRTP
YFQPFLKGTLGIAIRRGEIQGLHSFLLRLHPKSDQRNNIVRIFWETMFGCSFETGDEVTFVQQ
MKKVCTGQEDLSITNTPYTDVSGLRAPYNVYKAVYALAHALHDLMQCKEKRGPLSENSCAD
ITDLKPWQLVPYLKKVNFTTGFGDPVSFDNNGDALAIYDVLNWQPSSEESIKLHTIGVVNDEV
ATGMVLRLNEDEKFFFFFCHFQAPRSVCSESCPPGTRKAMRKGFPVCCFDCLTCGDGEISN
TTDAIKCTVCPDEFWSNFNKDQCVPKEIEFLSYEDPLGISLTTASLLGTCFCALVMIIFTFHRNT
PIVRANNSELSFLLLLSLKLCFLCVLLFIGQPQLWTCQLRHAVFGISFVLCISSILVKTMVVIAVF
NSSRPEGTGAMKWFGAAQQRCTVLVLTALQVVICAVWLSTSPPTPHKNSQYVRSKIVYECAI
GSVAGFSLLLGYIGLLAAISFLLAFLARNLPDNFNEAKFITFSMLIFCAVWIAFVPAYVSSPGKY
AVAVEIFAIVASSFGVLVAIFAPKCYIIILHPERNTKKAIMGRE
>Dr_16_10_F
MWVILHIYFFISCNYMSVALMASSGTCQLQGHFTLNGMYQNGDFLIGGLFEIQYLKAFPGLS
FRTEPKLPHCENFYMTSFQQAITMVFAINEINNNPNLLPNITLGYQMYDNCLRLGVAFRAATA
LISGTEETLSDLNCKGPPPVIGVIGDPGSTHSIAISSVLGLFRMPMISYYATCSCLSNRKKYPS
FFRTIPSDAFQVRAMIQILRHFEWTWVGLLYSDDDYGINAALSFHHDVQQFGGCVAFSEILPN
DNNQMAVQHIVRVIQNSTAKVVVVFSTSSYLLPVIDEMLLKNITGRQWIASEAWSTSPVLLNP
RLRYVLGGTLGIAIRRGEIEGLDNFLLRLRPDNFSQNSMMRIFWENMFECNFDTIGGLRTKL
CSGQEDLRSKFTPYTDVSELRASYNVYKAVYALAHALHDLMHCEEGRGPFSENRCVDISNL
KPWQLVHYLQRVKFTTGFGDHVSFDKNGDALAIYDVLNWQLSSEESVTVRRIGVVDEGVTT
GKVFTLDENAIYWNFETNKPPRSVCSESCPPGTRQATRKGLPVCCFDCLPCGDGEISNTTD
TTECIACPNDFWSNPEKDQCIPKEVEFLSYEDPLGISLTTAAMLGTFICALVMTIFAHYRNTPV
VRSNNSELSFLLLLSLKLSFLCVLLFIGQPQLWTCQLRHAVFGISFVLCVSSILVKTMVVIAVFK
SSRPEGKNAMKWFGAAQQRGTVLILTALQVLICAVWLSTASPTPHKNSRYIRSIIVYECAIGSV
AGFSLLLGYIGLLAAVSFLLAFLARNLPDSFNEAKFITFSMLIFCAVWIAFVPAYVSSPGKYAVA

VEIFAILASSFGLLLAIFAPKCYIIILHPERNTKKAIMGKG
>Dr_16_11_F
MWIIAKICLYLSFSCISVASIFRSGPCQLQGQFRLNGMFQEGDLILGGLFEVHFLTVFPEQSFR
TEPEPPYCEQFDMASFQQAQTMVFAIDEINRNPNLLPNITLGYYLYDNCVKLSVAFRAAMALV
SGTEESFSSLNCTGPPPVIAIVGDPGSTHSIAISSILGLFRVPMVSYFATCSCLSDRKTYPSFF
RTIPSDAFQVRAMVQILKRFGWTWVGLLYSDDDYGIYAAQSFQKEMQLFGACVGFSEMLPR
DNNHEDIQSIVEVIQTSTARVVVVFSTEAYLLPLMDEVALKNVTGRQWIASEAWATSPVFHTQ
HLLPFLGGTLGIAIRRGEIQGLKEFLYYLHPDSNTRNNIVRIFWENIFGCSFEIGGRERKVCTG
QEDLKSTNTAYTDVSGLRASYNAYKAVYALAHALHDLMQCEDGRGPFSGNKCADQINLQP
WQVVHYLQKVNFTTGFGDHVSFDENGDALAIYDVMNWQPSSDGAISVSTVGVVNEGASMK
MVLTLKENSIFWNFKNRKPPQSVCSESCPPGTRQVRRKGLPVCCFDCLPCADGEISNTTDAI
ECKICPDELWPNPNKDQCVPKEEDFLSFEDPLGISLTTASLLGTCFCALVMVIFSHHRNTPVV
RANNSELSFLLLLSLKLCFLCVLLFIGRPKLWTCQLRHAAFGISFVLCVSSILVKTMVVIAVFKS
SRPESKSAMKWFGVAQQRGTVMALTTLQIVICTVWLSMASPKPYKNSLYISSKVVYECDIGS
VVGFSLLLGYIGLLAAVSFLFAFLARNLPDNFNEAKFITFSMLIFCAVWITFVPAYVSSPGKYAV
AVEIFAILASSFGLLVAIFAPKCYMILFHPERNTKKSIMGRA
>Dr_16_12_F
SPLIICLYLSYNFKSAASILMGSCQLQGHFGLNGMYQAGDVILGGLFEVHLLAVFPELSFRSA
PEPPYCEQFDMASFQQAQTMVFAIDEINKNPKLLPNITLGYHLYDNCVMLGMAFRAAISLVS
GTEELFSNLNCTGSPPVIGIVGDSNSTPSIAISSVLGLFRVPIVSYFATCSCLSNRKQYPSFFRT
IPSDAFQVRAMVNILKHFGWTWVGLIYSDDDYGNYAAQSFLQDIQIYGGCVAFTEILPLNNNR
KYIEHVVGVIQASTARVVVVFSTSTYVLPLMDEVVLQNVTNRQWIASEAWATSPMFHTQRLLP
FLGGTLGIAIRRGEIQGLHEFLLHLRPKNDQHNNMVRIFWEKMFGCRFNPGGKGDKQCSG
QEDLSSIDTAYTDVKELRASYNVYKTVYALAHALHDLTECDEERGPFSENRCADITNLQPWQ
LVHYLQKVNFTTGFGDHVSFDKNGDVLAIYDVMNWHPSSDGSISVRTVGVVNEGAASGKVL
TLDEDAIYWNFKIKKAPQSVCSESCPPGTRQATKKGLPVCCFDCLPCGDGEISNVT
SDAIECTVCPDEFWSNKDKDQCVPKEVEFLSYEDPLGISLTTACLLGTCFCALVMIIFCQHRN
TPIVRANNSELSFLLLLSLKLCFLCVLLFIGRPQLWTCQLRHAVFGISFVLCVSSILVKTMVVIAV
FKSSRPEGKGSMKWFGTTQQRCTVLILTALQVVICIVWLSNSSPAPHKNSQHISSKIVYECAI
GSLAGFSLLLGYIGLLAAVSFLLAFLARNLPDNFNEAKFITFSMLIFCAVWIAFVPAYVTSPGKY
AVAVEIFAILASSFGLLGAIFAPKCYIILLHPEKNTKKAIMGRQ
>Dr_16_13_F
MWIILNIFLCLSKWISADSVLRLDTCQLQGHFMLNGVYKHGDVILGGLFDVHLLTVFPQLSFR
VQPKPPYCEKQVMENFKSSQTMAFTIDEINNNPNLLPNITLGYHLYDNCVRLAMAFRAAMS
LASGTEESFSNLNCTGPPPVIGIVGDPSSTPSIAISSILGLFHVPIVSLYHYATCSCLSDKKKYP

SFFRTIPSDAFQVQAMVQVLRHFGWTWVGLLYSDDDYGTYAAQSFQKEVQLFGGCIAFSTIL
PQNNPREIQNIMGVIRASTARVIVAISTSSYLLPLMDEVVLQNLTGRQWIASEAWATTPAFRTP
RFLTILRGTIGIAIRRGEIQGLLEYLLRLRPSSDPRNNLVRIFWENMFGCSFASQTVGEQVKKL
CTGQEDLSITNTAYTDISGLRGPYNVYKAVYALAHALHGLMQCEEGRGPFSGNSCADITNLK
PWQLIHYLQKVNFITGFGDRVSFDKNGDALPIYDVMNWQPSSDGSIRVQTVGVVNKGVTSG
MVLNLVEDAIYWNFETKKPPQSMCSESCPLGTRKARRKGLPVCCYDCLPCGDGEISNRTD
ATECLVCPYEFWSNKEKDYCVPKQVEFLSYEDPLGISLTTASLLGICFCALVMVIFSHHHNTPI
VRANNSELSFLLLVSLKLCFLCVLLFIGQPQLWTCQLRYAVFGISFVLSVCSILVKTMVVIAVFK
SSRPEGKDAMKWFGLLQQRCTILVLTTIQVVICTVWISNASPTPHKNHQYIRSKIVFECAIGSV
AGFSLLLGYIGLLAAISFLLAFLARNLPDNFNEAKFITFSMLIFCAVWIAFVPAYVSSPGKYAVAV
EIFAILASSFGLLMAIFTPKCYIILLHPERNTKKAIMGR
>Dr_16_14_F
MWTTLYIFLLFRCFSSESALRSGSCQLQGRFKLEGMYQDGDFILGGLFHVHFFTVFPELSFQ
TEPEPPYCEKFNIEIFQQAQTMAFAVDEINRNPNLLPNITLGYHLYDNCVRLGIAFRAAISLAS
GTEESFSNLNCTGPPPVIGIVGDPSSTPSIAISNILGLFRVPIVSHYATCSCLSDRKKYPSFFRTI
PSDAFQVRAMIQILRYFGWTWVGLIYSDDDYGIYAAQSFQQEMLLFGYCVAFSAILPHDNNH
RDIQRITAIIQASTARVVVVFSTSSFLIPLMEEVVVQNMTGRQWIASEAWTTSPVYHTPRFLPIL
GGTLGIAIRRGEIEGLHDFLLRLIPSNDKKNSIIRIFWENIFGCSFEKWGTETFGEQVKNICTGQ
EDLSTTDTPYTDVSGLRAAYNVYKAVYALAHGLHDLMQCEEGRGPFNGNSCAETTNLKPW
QLVHYLQNVNFTTGFGDQVSFDKNGDALPIYDVLNWHPSTDGSIKLHTVGLVNKGAAMEMV
LTLDDDAIYWNFETRKPPQSVCSESCPPGTRQARRKGLPVCCFDCLPCGEGEISNATGAIEC
TVCPDEFWSNPEKDQCVPKEVEFLSYEDPLGISLTTASLLGTCFCALVMIIFALHRNTPIVRAN
NSELSFLLLLSLKMCFLCVLLFIGRPQLWTCQLRHAVFGISFVLCISSILVKTMVVIAVFKASRP
EGKGAMKWFGATQQRCTVLVLTALQVVICVVWLLTASPTPHKNNQYIRSKIVYECAIGSVAGF
SLLLGYIGLLAAISFLLAFLARNLPDNFNEAKFITFSMLIFCAVWIAFVPAYVSSPGKYAVAVEIF
AILASSFGLLVAIFAPKCYIILLHPERNTRNAIMGRE
>Dr_16_15_F
ITLYLSLCLCFKHIYADSIHRLRSCQLQGRFKLNGMYQDGDFILGGLFHVHFFTVFPELSFRM
EPQQPYCEKFNMEGLQHAQTMAFAINEINKNPTLLPNITLGYHLYDNCVMLRMAFRAAMSL
ASGTEDSYSNLNCTGPPPVIGIVGDASSTPSIAISSVLGLFRVPIVSHYATCSCLSDRKKYPSF
FRTIPSDAFQVRAMVQILKHFKWTWVGLLYSGDDYGVYAAQMFHKEMQLSGHCVAFSEILP
NDHNPKDIQRIIRVIQGSTARVVVVFALSSFLIPLMDEVVLQNMTGCQWIASEAWATSLEYHTP
RFLPFLGGTLGIAIRRGEIEGLHEFLLRIRPSNDTSQNIVRIFWENLFGCRFETGGLKTGREQE
KMECTGQEDLSTTNTPYTDVSGLRASYNVYKAVYALAHGLHDLMQCEEGRGPFIGNSCADI
TSLKPWQLVHYLQNVNFTTGFGDHVTFDKNGDALAIYDVLNWQPSSDGSITVHKIGVVYEGA

TTGMMLTLDADAIYWNFETKKVTNLPPLSVCSESCPPGTRQATRKGLPLCCFDCLPCGDGE
FSNTRDAVECMMCPDEFWSSPDKDQCVPKEVEFLSYED
LGISLTTASLLGTCFCALVMIIFALHHNTPIVRANNSELSFLLLLSLKLCFLCVLLFIGRPQLWTC
QLRHAVFGISFVLCVSSILVKTMVVIAVFKSSRPEGKGAMKWFGASQQRCTVLVLTALQVVIC
AVWLSTASPTPHKNNHYFRSIIVYECAIGSVAGFSLLLGYIGLLAAISFLLAFLARNLPDNFNEA
KFITFSMLIFCAVWIAFVPAYVSSPGKYAVAVEIFAILASSFALLLAIFAPKCYIILLHPERNTKNAI
MGRE
>Dr_16_16_F
MWFTLYIYICLSLNCISATLILKPSMCLLQRSFKLNGMYQDGDFILGGLFEVHFFTVFPDLTFT
TNPKSPYCEIFNMEGFQHAQTMAFAIEEINKNPKLLPNITLGYHLYDNCVMLGMAFRAAISLT
SGTEESFLNLNCSGPSPVIGIVGDPSSTPSIAISSVLGLFRVPIVSHYATCSCLSNRKKYPSFF
RTIPSDAFQVRAIVQVLKHFKWTWVGLLYSDDDYGVYAAQSFQQEMRQFGLCAAFSEFLPH
DNNPRTIQHIMGVIQGSTAKAVVVFAPSSFLIPLMNEVVLQNMTGRQWIASEAWATSLESHIP
SFQPFLRGTIGIAIRRGEIQRLHDFLLRIRPSNDPKNYMLRIFWENMFGCSFGKGDTEGEQVI
KVCTGQEDLSTTNTPYTDVSGLRAAYNVYKAVYALAHALHDLMECEEGKGPFSKNSCADKT
NLKPWQVVHYLQNVNFTTGFGDYVSFDKNGDALAIYDVLNWQPSSDESIRIYTVGVVKEGTE
TGMVLTLDEDAIFWNFETRKPPRSVCSESCPPGTRRATRKGLPVCCFDCLPCGDGEISNTT
NAVECFLCPDEFWSNQYNNHCVPKEVEFLSYEDPLGISLTTASLLGTCFCALVIFVFALHRNT
PIVRANNSELSFLLLLSLKLCFLCVLLFIGRPQLWTCQLRHVVFGISFVLCMSSILVKTMVVIAV
FKSSRPEGKGAIKWFGAVQQRSTVVVLTVLQVLICAVWLSTASPTPYKNNQYIRSKIVYECAI
GSVVGFSMLLSYIGFLAAASFLLAFLARNLPDNFNEAKFITFSMLIFSAVWIAFVPAYVSSPGK
YTVAVEIFAILASTFGLLIAIFAPKCYIILLHPERNTKNVIMGRE
>Dr_9_13_FN
MSIHVLSWLTLSVPVLLLWPVCGSGTEVTPLETCVYLITPQYSDELGTYQDGDVIIGGLINLHN
LAATPDLSFTRKPGLAQCLEFQERTYRWFQAIVFTVEEINQHPSLLPGVKLGYHIMDSCSRYP
HSLTAAMSMISGGNKTCGPTKPAKLLIGDSSSTQSILLSTTLVPLKIPMISYLAGCPCLSDRQK
YPNFFRTIPSDFYQARTMVQIAKRFGWTWIGAVIADSDNGHGTLQALEEEIKGTGICLAFYHT
LYRERLQKDVAFAARTVQASSARVILVLAWYTDVEAFLXXXSILHTEHIIQSRFYRSYECGAL
FTVVKWIISWQIHNDSLLTDNNNNNNNYYYYYYYYYKALXXXXDL
QLLQHVKDVQYTTQLGEEFYXLEGGIPPVYDLVNWQIAPDGSLQYAFIGHVDGNQLSINDSAI
TWPGDSGKVPISVCTSECPPGTRKAIKKGLPVCCFDCLPCTEGEISNSTGSIKCYRCPKEFW
SNSYKNECVARETEFLSVKETMGITLMSVAVSGAVMTTTVAVIFLYHRNTPIVKANNSELSFLL
LLSLKLCFLCALVFVGQPSLWSCRIQQAAFGISFVLCISCILVKTFVVLIAFHSTRPESSALIKWF
GLGKQRGIVLVFTCVQVVICAIWLCVSPPLPYQNFGMHRSKVILECTIGSVVGFTCVLGYIGFL
ATVCFLLAFFARKLPDNFNEAKYITFSMLIFCAVWVAFVPAYVSSPGKYSVVVEVFAILASSFGL

LFCIFVPKCYIILLKPENNTKKFLMGKE
>Dr_V2R2_F
MDLTGLSYEGRFLLVLCMISYLFTPTDAEGSCKLKAKFNLRGYKEVEKTTVVIGGMFPVHRSL
VSTDSNTTDPPESVDCQGFNFRAFRWAQTMLFALKEINSRTDLLPKTELGYVIYDSCFTISKA
VEGTLTFLTGQDEAVPNYRCGNGPPLSALVGAGGSDLSIATARILGLYYFPQVSYESSCSVLE
SRFQYPTFLRTIPSDENQSVAMAKLVLRFGWTWVGTIAAEDDYGKYGIKRFKEVVEEAGVCIS
FSETLPKISNPEAIQRIVQTVHDSTAKIIVVFSSDVDLSPLVEALLQSNVTNRTWIASEAWVTSA
AISRQPNVLSLLGGTIGFAVKRAEIPGLKKHLLSISPFNDSLTEEFWGIVFNCTTNYTLILKGMR
RCTGEEMLGTVDNTYSDVSQLRITYNVYKAVYAVAHALHNLEQCKTGSGPFENDTCADITNF
EPWQLMYYLIHLRFTVPHTGEELFFTNGEVEGFYELLNWQSDSNGGITYTHIGYYNSTAAPE
DKLVINNNSIIWNNNVLKAPRSVCSERCQPGTRMGIRQGEPVCCFDCIPCADGEISNTTDAR
GCIQCDGDYWSNANHDECVPKTIEFLDFSEPLGITLIAIAAFGALATIVVAIIFLMHLNTPLVNVN
DPLLTFSLLLGLVITFLCSIVFLGKPQMWSCMTSQMALAVGFALILSSLMGKSALLMLRARAV
KAVKAAAKAAKAAAAASEQSPDTAVFAPAIPQKNDVDPIHPRHQRAIMILCTLIQVVGCTAWLI
LMPPHPVKNTGVQNIKIILECDPGNIIFICSIFGYDILLALVTFAFAFVARKLEDHFNEGKSVTFG
MLVFFIVWSSFVPAYLSTRGKFMVAVQIFAILASSFGLLACVFIPKCYVLLVKPERNKEEMMIPR
>Dr_9_7_F
MLLYLCTLLLFFKLKVSVGNASCKIMGEPKYPLLFKDGDVTIGALFPVHSIETAPSFEFTQKPQ
LLSCSSVNLRDFRLAQILIFAIEEINRSESLLPKLSLGYKIYDTCSSKLSSMSATMALMNSFEFA
GRDKCNGQTSVHAIIGETESSATVILTRTTGPFKIPVISHAASCECLSSRKEYPSFFRTISSDYH
QGRALAYIVKHLGWSWVGAVNSDNDYGNYGMAIFLNTAHKEGICVEYSEKFYRTEPEKLKK
VVDTIKKSTAKVIVAFISFLEMRLLVEQLSAENITGLQIVGVEGWTTSKSLITPKTLNVLRGSLGF
AMRKIYIEGFAEYVLKPFWDTAFPCIPNQRNDSWVILNCSRYQDLLVLKNYIEDVPEHRFSINV
YNAVYAVAHALHSLFKCKEQEGCEKDLLTQPQQVVDALKKVNFTVKMGDRVWFDSTGATLA
QYEVVNWQQNSDGSVNFKKVGYYDASLPPDQRFVLNIKEILWAGGNLKANPRSVCSESCP
PGTRKAAQKGRPICCYDCIPCADGEISNDTDSNNCKQCQGEYWSNADKNKCVQKSVEFLS
FTEVMGIVLVFFSLFGAGLTVLVAILFYSKKDTPIVKANNSELSFLLLFSLTLCFLCSLTFIGQPT
QWSCMLRHTAFGITFVLCISCVLGKTLLVLMAFKATLPGSNIMKWFGPVQQRLSVLAFTFIQV
LICVLWLSISPPFPHKNMKYYEEKIILECSLGSTIGFWAILGYIGLLAALCFILAFLARTLPDNFN
EAKFITFSMLIFCAVWITFIPAYVSSPGKFTVAVEIFAILSSSISLLLCIFAPKCYIILLKPEQNTKQY
MLGKT
>Dr_8_1_YP
EPLVALLHMVIAIMTFSKANETTCTLQGEPVLPELWKDGDIIVGGVFSFHSSWEVRQLTYTFVP
PPLKCISLNFRDFQYAQSMLFAIEEINNSSTLLPGVSLGYKIYDTCGSVAVGVRAAMALANGH
EKISVEGPCTKHAEVQAILGDTTSSACMAITKSIGPFKLPLISHYATCECLSDKVKYPSFLRTIA

SDHYQSRALAELVRHFGWTWVGALRTDDDYGNSGMATFTKVAEQMGICLEYSLPFFRTYTE
DKVMRIIEQIKSSTSRVIVGFLAHWDLEVLLHKFVEYNITGYQWVGTEAWISDSVIASMDTHHI
LQGAVGLAIPKTKVTGLQEFILNITPLKSSGGAIFSEFWEALFQCKYSNKDTSVSINACTGKEE
LSQVENLFTDMSLMPIFSNVYKGVYAVAHALHELLGCKDKCALKKQPDPVTFLKHIRKVHFK
TKDGEEVYFDENGDPVAKYDIINWQQSTKQHYEFVTVGFYDASFMGMDRLAVNMSSIFWAI
NSTKVPVSVCSESCPHGTRKAVKKGKPICCYDCISCTEGEISNTTG
>Dr_9_8_F
MLLFLYSVLSLYHLNTKAENTLCQMIGNPKYPLLSKDGDITIGALFVIHSEVSLPSFAFTQKPN
LLSCSSLNIKDFRLAQTMIFAIEEINKNTNLLPNISVGYQIYDTCGSRFHSMSATMALMNGPK
NSEGYTCNEQSSVHAIVGETESSNTIILSRTTGPFKIPVVSKKYSTTCECLSNRKEFPSFFRTIA
SDYHQSRALAYIVKHFGWTWVGAVNSDNDYGNNGMATFQKTAKEVQICVEYSVKFIRTETE
KIRNVVNVIKKGTAKVIVAFLTGFEMKSLLEQLSSQNITGLQMIGVEAWITSKTLITSKSFHVLG
GSLGFAVRKIQIEGFADYVMKAFWDTAFQSFSNADLNYSQYQDLLLVKNYNEDVLEQRFVSY
VYKAVYALAHSLHSLLRCTEQGGCGKALTIQPHQLVEALRKVNFTVKMGDQVWFDSTGGVIA
QYDVVNWQQNSDGSVQFQSVGYYDASLSPDQRFMLNTEKIVWAGGQLEKPRSVCSESCP
PGTRKASQKGRPVCCHDCIPCADGEISNETDSNNCKQCPGEYWSNANKNKCVTKAVEFLS
FTEVMGIILVFFSLFGAGLTVLVAILFYSKKDTPIVKANNSELSFLLLFSLTLCFLCSIPFIGRPTQ
WSCMLRHTAFGITFVLCISCILGKTIVVLMAFKATLPGSNIMKWFGPVQQRLSVLAFTLIQGLI
CVIWLTKSPPFPYKNMKYFQEQIILECSVGSTIGFWAVLGYIGLLAVLCFILAFLARTLPDNFNE
AKFITFSMLIFCAVWITFIPAYVSSPGKLTVAVEIFAILASSFGLLFCIFAPKCYIILLQPEQNTKQQ
MMAKT
>Dr_9_9_F
MILYFYTILFLRCFQAKTENAFCQIIGEAKYPLLSKDGDVTIGGIFAIHSKETLPSFEFQQKPQP
LLCSSVNLRDFRLAQIMIFAIEEINKSENLLPNVSIGYRIFDTCGSRLSSMSATMGLMNELKFA
AGETCNGQSPIHAIIGETESSATVILSRTTGPFKIPVISHSASCECLSNRNDYPSFFRTISSDYH
QGRALAYIVKYLGWSWVGAVNSDNDYGNYGMAIFLETAQKEGICVEYSVKFYRTETEKLKRV
VDTIKKSTAKVIVAFVSFIEMGLLIEQLSIQNITGFQIIGVEGWITSKNYITTNSFHSMGGSLGLA
LRKIHLEGFLDYVTKSFWSTAFPCSQTEGILPTVGCSKYKDLLPLKNYTEDVPEHRYSSHVYK
AIYAVAHSLHSLLKCKEGEDCEKGHATQPQQVVEALKKVNFTVKFGDRVWFDRTGATVAHYE
VVNWQQDTDGSFQFKQVGYYDASLPPDQRFVLNIESIIWPGGNLEKPRSVCSESCSPGTRK
AAQKGRPVCCYDCVPCADGEISNETDSVNCKQCPREYWSNGEKNKCVLKAIEFLSFTEIMG
IVLVCFSLFGVGLTAVVAILFWSKMDTPIVKANNSELSFLLLFSLTLCFLCSLTFIGRPTEWSCM
LRHTAFGITFVLCISCVLGKTIVVLMVFKATLPGSNVMKWFGPTQQRLSVLAFTFIQVLICVLW
LTISPPFPNKNMTYYKEKIIIECSLGSTISFWAVLGYIGLLAVLCFILAFLARKLPDNFNEAKFITF
SMLIFCAVWITFIPAYVSSPGKFTVAVEIFAILASSFGLLLCIFAPKCYIIICKPEQNTKQHVMGKT

>Dr_9_10_F
SGMFLFYTILLFHLLHTKVENSLCRIMGDSNYPLFSKNGDVSIGGIFAIHRKETLPSFEFMQKP
QPLSCSSVNLRDFRLAQTMIFAVEEINRSKSLLPNVSIGYKIYDTCGSRLSTMTAIMGLMNGQ
DFSTEDRCNGQSRLHAIIGESESSATIVLSRTTGPFRIPVISHSSSCECLSNKKDYPSFFRTISS
DYHMSRALVYIVKHLDWSWVGAVNSDNDYGNNGMAIFLKAAHEEGICVEYSVKFYRTEPEK
LKKVVDTINKGTAKVIVAFVSFVEMGLLIDQLSIQNITGIQMIGVEPWITANTYITSNSFRAMGG
SLGFATKYIYIEGFAEYVMTPFWNTAFPCSESDRNHSHYELICSRYEDLLALKNDNKDVNEHR
YSSNVYKAVYAVAHSLHGLLNCKEQEGCEKGLTIQPQQVVKALKNINFTIKSGDSVWFDNTG
SVVALYEVVNWKKT
DGSFQFKSVGFYDAMMPPYKNLRLNTKNIVWAGGQLEKPRSVCSESCAPGTRKAAQKGR
PVCCYDCIPCAEGEISNETDSINCKQCPGEYWSNTERNRCVIKAVEFLSFSEVMGIVLVIFSLF
GAGLTVLVAVLFYSKKDTPIVKANNSELSFLLLFSLTLCFLCSLTFIGQPTKWSCMLRHTAFGIT
FVLCISCVLGKTIVVLMAFKATLPGSNVMKWFGPVQQRLSVFAFTLIQVLICVLWLTISPPFPY
KNMKYYKEKIILECNLGNTIGFWAVLGYIGLLAALCFLLAFLARKLPDNFNEAKFITFSMLIFCA
VWITFIPAYVSSPGKFTVAVEIFAILSSSFGLLVSIFAPKCFIILLKPEQNTKQHVMGKT
>Dr_9_6_F
MLLFLYIFLHFTLIRTKAGNGLCQMMGDPKYPLFSKDGDITIGALFAILSKETLPSFQFTQKPQ
LLSCSSVNLRDFRMTQIMIFAIEEINRSKSLLPNVSIGYRIYDTCGSRMSAMSATMALMNGPE
FTADKICNGESPIHAIIGETESSATIILSKTTGPFKIPVISHSASCECLSNRKIYPSFFRTIASDYH
QGRALAFIVKHFGWSWVGTVNSDNDYGNNVMAIFLNTAQKEGICVEYSVKFYRTEPEKLKK
VVETIKKSTAKVIVAFVSLVEMGLLIDQLSIQNITGFQVIGGEGWITTKSLINSKSFHVLGGSLGF
AFRKIIIEGFADYVIKTFWERDFPCLTNEGNYSQYALTCSSYQDLFTLKHYNEDIPEQRYASNV
YKAVYAVAHSLHNLLKCKENEGCEKSLTMQPQQVVEALKNVNFTLKFGDRVWFDSTGGAVA
HYEVVNWQQDSDGSFQFKKVGYFDASLPPDQSFMLNTKNIIWSGGQLERPRSVCSESCPP
GTRKASQKGRPVCCYDCIPCAEGEISNETDSVNCKQCQGEYWSNVEKNKCVLKAVEFLSFT
DFMGIVLVFFSLFGVGLTVLVAILFYSKKDTPIVKANNSELSFLLLLSLTLCFLCSLTFIGRPTEW
SCMLRHTAFGITFVLCISCVLGKTIVVLMAFKATLPGSNVMKWFGPVQQRLSVLAFTLIQVLIC
VLWLTMSSPFPYKNMKYYKEKIILECSLGSVIGFWAVLGYIGLLAALCFILAFLARTLPDNFNE
AKFITFSMLIFCAVWVTFIPAYVSSPGKYTVAVEIFAILASTFGLLFCIFVPKCHIIIFKPEQNTKQ
HMMGKN
>Dr_5_2_YN
FNVFSILVSICPYYIYFMSAEVKTDKYQHPQDVTGXXXXAL
HFSCIDSLIYS
IFFFFFCSLSLGEFQNAQTMIYAIEEINKRADIISGQSLGYRIYDSCGSTDMALKASLSLVNGEN
ASQLYCQRPLTVQAIIAETYSTPTIAISATVGLLHLPVVSHYATCACLSDKKKHPSFFRTIPSDY

YQSRALAKLVQYFGWTWVGALCSDNDYGHNGMNTFIKAATEFGVCVEFSESFFRTHPREEI
LRIVNIVKKSSSKVIVAFVSYADMEVLLLELAKQNITGLQWIGSESWISDMNIATGKWQYILRG
SMGFAIPKSEIQGLKEFLTKVNPSSNIYLYKELWESVFQCRLSPEQSSESKSMCKGNESLNH
VQNQYTDVTELQIPNNVYKAVLAIAHALNSSVGCLKWDKNQRECLEKINNTWQVLQALREV
SFFTETGEKVFFDKNGDPAARYDLLNWQQGEEGATKFVKVGFYDASLQPEFQLSFNNITIM
WAKVPVSVCSESCPMGTRKAVKKGKPICCYDCIQCAEGEISNKTDSVMCLKCPPEFWSNK
WRDTCVPKLVEFLSFEDVMGIVLIIFSLLGVSFTLGIAIIFFVHKDTPIVKANNSELSFLLLFSLTL
CFLCSLTFIGRPTEWSCMLRHTAFGITFVLCISCVLGKTIVVLMAFKATLPGSNVMKWFGPLQ
QKLSVITFTLLQVVICVLWLTLSPPFPYMNMNYYQERIILECNLGSAFGFWAVLGYIGLLAILCF
ILAFLARKLPDNFNEAKFITFSMLIFCAVWIL
>Dr_16_19_F
MWISLNIFLYLSLNCIFAASVVNPGTCQLQGHFKLNGMYQDGDFILGGLFEVHFFTLFPKLSF
KSEPEPPYCEKFDMESFQQAQTMVFAIDEINKNPNLLPNITLGYHLYDNCVMLGMAFRAAIS
LASGTEEYFSNLNCTGPPPVIGVVGDPGSTPSIAISSVLGLFRVPIVSHYATCSCLSDRKKYPS
FFRTIPSDAFQVRAMVRILSHFGWTWVGLIYSDDDYGIYASVSFQQEMQQFGSCISFSEILPH
DNNPKDIQRIMEVIKASTASVVVVFSTSSYLMPLIDEVVLQNLTGRQWIASEAWATSPVFHTPR
YLPFLGGTLGIAIRRGEILGLQEFLLHIHASNNPKDNMLKIFWENMFRCSFETRSKDTIGEQE
KSICTGHEDLSTTKTPYTDVSGLRASYNVYKAVYALAHALHDLMECEEGRGPFDGNSCGNIT
NLKPWQLVHYLQKVNFKTGFGDHVSFDENGDALAIYDVLNWHPSSDGSIRLHTVGVVNEGA
GTGKVLSLDESALYWNFQTKNPPRSVCSASCPPGTRQAMRKGLPICCFDCLPCGDGEISN
ATDSTECVTCPDEFWSNFDKNQCVPKEVEFLSYEDPLGISLTTASLLGTCFCALVMIIFALHLN
TPIVRANNSELSFLLLLSLKLCFLCVLLFIGQPQLWTCQLRHVVFGISFVLCISSILVKTMVVIAV
FKSSRPEGKGTMKWFGAAQQRCTVLVLTALQVVICAVWLSNASPTPIKNNQYIRSKIVYECAI
GSVAGFSLLLGYIGLLAAISFLLAFLARNLPDNFNEAKFITFSMLIFCAVWIAFVPAYVSSPGKY
AVAVEIFAILASSFGLLVAIFAPKCYIILLHPERNTKKAIIRRE
>Dr_9_14_YP
MSVFFCTLLIFFQLYAKAEKPICIMMGDPKYPLLSKEGDISIGAVFPVHSIETLPLFKFTQKPQL
LSCSSVSIRDFRMAQIMAFAIEEINRNESLLPNVSLGYQIYDTCGSGLSLMGANMALMNVQE
FASRGSCNGQSPVHAIIGETESSNTVILSRTTGPFKIPVISPSASCECLSNRKEYPYFFRTIASD
YHQSRALAYIIKYFGWSWVGAVNSDNDYGNHGMAIFLSTAEKEGVCVEYSVKFQRTEPEKL
KKVVDTIKKGTSKVIVAFLTEFEMKNLLEYLIIQNVTGLQVIGVEAWITANSMIKPNSFHVLGGS
LGFAVKKLNIEGFEDYVTKAFWETAFPCSQTSQKENSQYKLICNIYRDLLVLKNDNKDVPEQR
YASNVYKAVYAVAHSLHNILKCRENGACETDMKIQPQQVVEALRKVNFTIKMGDCVWFDST
GAVVAQYEVVNWQPDYNGSIQFKPVGYYDASLPPNQRFVINTENIIWAGGQLKV
>Dr_9_11_F

MVSFIWTLLLVVPLQAKAENSLCRMMGQNINPLISKEGEVTIGALFPIHSIEILPSFEFTVKPQL
LSCSSVNLRDFRMAQTMTFAIDEINKNQSLLPNVSIGYRIYDTCGSRLSSMSATMALMNGKE
FSAEDKCNGQSAIHAIIGETESSATVILSRTTGPFKIPVISHSATCECLSNRKNHPSFFRTIASD
YHQSRALAYIVKHFGWSWVGAVNSDNDYGNNGMAIFLNTAQEEGICVEYSEKFYRTDPEKL
RKVVDTIKNSTAKVIVAFLTSLEMENLLQELTKANITGLQIIGVEAWITANSLLTPNSFRVLGGSL
GFAVPKVNIQGFSNYVIKDFWETAFPCSETEINVSQYSSLSCNSYDDLLLLKNYNEDVPEQRY
ASNVYKAVYAVAHALHSLLKCKVNEGCKKDLKIQPQQVVDTLKEINFTINMGDRVWFDSTGA
TIAQYEVINWQQGSDGSIQFKTVGYFDASLPHDQRFVLNTESIIWTGGQLEANPKSVCSENC
PAGTRKAVQKGRPVCCYDCIPCADGEISNDTDSVNCKQCPGEYWSNAEKNRCVLKTVEFL
SFTEVMGIVLVFFSLFGVGLTVLVAILFYSKKETPIVKANNSELSFLLLFSLTLCFLCSLTFIGRPT
EWSCMLRHTTFGITFVLCISCVLGKTVVVLMAFRATHPGKDIMKWFGPVQQRLSVIALTLIQV
LICVLWLTISPPYPYKNMKYFKEKTILECNLGSTIGFSAVLGYIGLLAVLCFFLAFLARTLPDKF
NEAKFITFSMLIFCAVWITFIPSYVSSPGKLTVAVEIFAILASSFGLLFCIFAPKCYVILLKPEQNT
KQHIMGKI
>Dr_9_12_F
MSVFLYTVLIFIFYTKAENPLCQMMGNPKFPLLSKDGDVNIGAIFSVHSTEILTSFTYTQKPQL
LSCSSVSLRDFRMVQTMIYAIEEINRSLGLLPNITVGYQIYDACGSRLSAMSATMALMNGPEF
TWRDRCTGQSPIHAIIGETESSATVILSRTTGPFKIPVISPSATCECLSNRKEYPSFFRTIASDY
HQSRALAYIVKHFGWSWVGAVNTDNDYGNNGMTTFLNTAQEEGICVEYSVKFYRTEPEKLQ
KVVETIKKGTAKVIVAFLTSSEMYNLLEQLSIQNITGLQMIGVEGWITAKSLITPNSFHVLGGSL
GFAVRKTAIEGFADYVIKSFWETAFPCTMTIGNSSQYSLSCGIYQDLLLLKNYNEDVPEQRYS
TNVYKAVYAVAHSLHSLLKCKEDGCKKGLAIQPQQVVGALKKINFTLKLGDIVSFDSTGATVA
QYEVVNWQKDANESIKFKPIGYYDASLPPHQRFVLHTENIIWAGGQLDRPRSVCSESCPPG
SRKAAQKGRPVCCYDCIPCAEGEISNQTDSNNCKQCPGEYWSNAEKNKCVLKDVEFLSFT
EIMGIVLVIFSLFGAVLTALMAILFYRKKDTPIIKANNSELSFLLLFSLILCFLCSLTFIGRPTEWSC
MLRHTAFGITFVLCISCVLGKTIVVLMAFKAALPGNNIMKWFGPVQQRLSVFALTLIQVLICVL
WLTMSPPFPHKNLKYYQEKIVLECNLGSNIGFWAVLGYIGLLAVLCFILAFLARKLPDNFNEA
KFITFSMLIFCAVWITFIPAYVSPPGKFTVAVEIFAILASSFGLLFCIFVPKCYIILCKPEKNTKQHL
MGKV
