# Supplementary material for: Evolution and origin of vomeronasal-type odorant receptor gene repertoire in fishes
Source: BMC Evol Biol. 2006 Oct 3;6:76. doi: 10.1186/1471-2148-6-76 (PMC1601972; doi:10.1186/1471-2148-6-76)
Supplement: Additional File 8 — Deduced amino acid sequences of medaka V2Rs. [file 1471-2148-6-76-S8.pdf]

>MF_12_1_F
MLRRARLLFFGFFCSVVHNTLQGQGTQCQKNPSATSLSVLQKRGDIILGGLFSLHDMVVEP
RLNFSSPSPPTKCTRFNFRTFRWMQTMIFAIEEINRVGALLPNFTLGYKIYDSCSTPHQALRA
ALELMGGESRSGLEGETLSQDTCHGAVPVVIGDGGSTQSLVVARFMGVFHVPQVSYFSSCA
CLSNKKEFPSFLRTMPSDFFQVGALAQLVKHFGWTWVGIVAGDDAYGRGGTKIFADEVKIKL
GACVAFNEIIPKNPSQSEISPIVDSIRSSGVHVILVFAVEQDVAAFFDEVVRSGLTGMQWLASE
AWSTAAILSNPRKYHHILQGTLGFAIRGVNIPGFRDFLLRLNPSSPNASEDPFLIPFWEEVFQC
SLDTQADDQKADGKPSCTGTEDLKNIQNTYTNVDQLRISYNVYKAVYAVAYALKAMRSCVR
GEGPFPQMTCPDADNVQPWQLLHFIKLVQFQNAFADEVKFDENGDVAAMYDLVNWQLKPN
GEMDFVTVGKFDEATHVSGKKLQIQEEDILWNGNHTKVPLSVCSSICPPGTRKAIRPNFPICC
HDCVACTAGKISNQTDTIECVRCLSEFWSNAERTACVPKQVEFLSFSDTMGITLMTISLTGSL
STSVVVLVFFLNRNTPIVKANNSHLSFLLLFSLTLCFLCPLTFIGQPSKWSCMLRHTAFGISSVL
CISCILGKTIVVLFAFKATLPGSKIIKWFGTAQQKSIITFSTLVQCGKTDLFCVQVIICAVWLVVAP
PRPHQMMPRESAVVILLCDEGSLVAFACVLGYIGLLACSCLLLAFLARKLPDNFNEAKLITFS
MLIFCSVWLAFIPAYISSPGKYATVTEVFAILASSYGLLGCIFAPKCYIILLRPGKNTRQQIMLRI
>MF_9_1_F
MKMAPLLTGLFFMGLLLACDTAGEDASDCHVLESPELPLLSKDGDVMIGGAFSIHSKIAQPS
LSFTEKPTPFTCSSINLREFRFAQTMIFAIEEINKSKHLLPNVSIGYRIYDNCGSTLSSMRAVMA
LMNGDELLIEKNCSGQSAVHAIIGESESSSTIVLSRTTGPYQIPVISHSATCECLSSRKEYPSFF
RTIASDLHQSRALAQLVKHFGWSWVGAVNSDSDYGNNGMAIFLSAAQEEGVCVEYIEKFDR
AEPEKLLKVVEVIRKSTARVIVAFLAHVEMNNLLEQLNVHNITGRQFIGVEAWITADSLVTPTSF
SVLGGSLGFAVQKTNISGLNTFLVEDFWETDFQCEENHIDVASKSCKENQDLMKLRDYNDD
VEELRYSSNIYKAVYAVAHSLHSIMKCSDSQGCDKTSEVKPWQVIEALKQVNFIIKNGEQVWF
DSTGAVLARYEVVNWQQRSTGSVHFEPVGYYDASLPPGQKFVLNTEAIMWPGGSTKLPVS
VCSESCRPGTYKVLERGKPVCCFHCLPCPDGEISNSTDSNDCNQCPEEFWSNQIRDACVP
KNVEFLSYTEDMSKILMFFTLFGVVLTVTVAVLFLINRDTPLVKANNSELSFLLLFSLSLCFLCS
LTFIGRPTEWSCMLRHTAFGITFVLCISCILGKTIVVLMAFKATLPSSNVMKWFGPVQQRLTVF
SLTIIQVIICILWLTINPPFPFKNMKIFREKIILECSLGSSLGFWAVLGYIGILALLCFVLAFLARKL
PDNFNEAKFITFSMVIFSAVWITFIPAYVSSPGKLTVAVEIFAILASSYGILFCIFAPKCYILALKPE
LNTKKSMLGKS
>MF_10_3_YP
MYVYIFVMSLLVVMEILAEDDSAVCEMLGSPEFPLLSKSGDIIIGGAFSVHSQIVASSFSLSDA
PDPLICTRLNFREFRFAQTMIFAIEEINKSSSLLPNISVGYKIFDSCGSTLPSTRAAMGLINGQE
RSFGKTCSGHSTVHVIVGTSESSSTLVLSQIAGIFQIPVISHFSTCACLSNRKEHPSFFRTIPSD
YYQSRALAQLVKYFGWTWVGAVRSDNDYGNNGMATFIEAATKEGICVEYSEAIARTASKEQV

ERVVRVISRGTARVLIAFLSYDEMDALLEAALSQNLTGLQWVGSESWITAHHLATMKYSGILY
GSIGFAIKKAKIAGLQDFLLQVHPSQDPQNHILKEFWEETFGCSFQSSLQDTKQCSGSERLR
DIKNPFTDVSELRISNNVYKAVYAVAHAMHSMLKCGENSETVNASCTLKSILQPKEVVKHIQD
VNFSLKSGEKVYFDERGDPAATYQLVNWQRNPAGDIVFVVVGSYDASLQNGKEFTMNGLNI
TWVTESFERPVSVCSESCPPGFRQAIIKGKPICCFSCITCAAGEISNCT
>MF_10_4_Y
MCFWISVMSLMVVIMGILAEEETANCELLGSPELPLLSKSGDIIIGGAFSVHSEMAASSLSLS
DAPDPLICTRVNFREFRFAQTMIFAIEEINNSSSLLPNISIGYKIFDNCGSTLPSTRAAMGLING
QERNFGETCSGHSSVHAIVGTSESSSTIVLSQIAGVFQIPVISHFSTCACLSNRKEHPSFFRTI
PSDYYQSRALAQLVKYFGWTWVGAVRSDNDYGNNGMATFIEAATKEGICVEYSEAIARTASK
EQVERVVRVISRGTARVLIAFLAQSEIDILLEEALSQNLTGLQW
GSESWITAXXXXIF
KYVLFFSPPPHLLIAIISKVYLFGNLKNLKQKQKLS*WQFNAKLMPLSPYQSFFYFFI
YLTIILAQISRSEKIL
FFLLSQRPVSVCSESCPPGFRQAMIKGKPICCFTCINCA
GEISNSTNSAECSPCPLEYWSNEDHNKCVPKVLEFLSYEETMGTLLTAFSLFGAGLTLVVSFV
FFWFRHTPLVKASNSELSFLLLFSLTLCFLCSLTFIGRPTDWSCMLRHVAFGIAFALCISCILTK
TITVVIAFKARTPVNTVPQCLASHQRMGVVGGLFCRC
>MF_10_1_F
MNNGICVMVLFMLTMGVIAEDETALCELLGSPVLPLLSKKGDITIGGAFSVYTQTPKSSPSLT
ETPEPLICSRISFREFQFAQTMMFAIEEINNSSSLLPNVSVGYKIFDSCGSTLPSTRAAMGLIN
GQERTFGKTCTGQSTVHAIVGASKSSSTIVLSQIAGIFQIPVISHFSTCACLSNRKVHPSFFRTI
PSDYYQSRALAQLVKYFGWTWVGAVRSDNDYGNNGMATFIEAATKEGICVEYSEAISGSSS
YEQVERVVRVIKTGTAKVLVAFLDSDGMDILLEEALRQNLTGLQWVGSESWITAHHLATMKYS
GILYGSIGFAIKKAKVAGLQDFLLQVHPSQDPQNHILKEFWEETFGCSFQSSLQGTKQCSGS
ERLRDIKNPFTDVSELRISNNVYKAVYAVAHAMHSMLKCGENSETVNASCTLKSILQPKEVVK
HIQDVNFSLKSGEKVYFDERGDPAATYQLVNWQRNPAGDIVFVVVGSYDASLQSGKEFTMN
GLNITWVTESFERPVSVCSESCPPGFRQAMIKGKPICCFSCITCAAGEISNSTNSAKCSPCPL
GYWSNEDHNKCVPKVIEFLSYEETMGTLLTAFSLFGAGLTLVLLFVFFWFRHTPLVKASNSEL
SFLLLFSLTLCFLCSLTFIGRPTDWSCMLRHVAFGIAFALCISCILTKTITVVIAFKARTPGNRVP
QCLASHQRMGVVGGTFLQVLVCVAWLAQAPPFPHKNTIYALERIILECNLGSSIGFWVVLGYI
GLLAVLCLILAFLARKLPDNFNEAKFITFSMLIFSAVWVTFIPAYVSSPGKFTVAVEIFAILASSFG
LLFCIFAPKCYILLLKPERNTKSHIMGRD
>MF_10_2_F
MHDCICVMVLFMLTMGVITEDETALCELLGSPVLPLLSKKGDITIGGAFSVYTQTPKSSPSLTE

TPEPLICTRISFREFRFAQTMMFAIEEINNSSSLLPNVSVGYKIFDSCGSTLSSTRAAMGLING
QERTFGKTCSGQSTVHAIVGASKSSSTIVLSQIAGTFQIPVISHFSTCACLSNRKVHPSFFRTI
PSDYYQSRALAQLVKYFGWTWVGAVRSDNDYGNNGMATFIEAAPQEGICVEYSEAISGTSL
YEQVERVVRVIKTGTAKVLVAFLGYDEMDILLEEALRQNLTGLQWVGSESWITSSNLALNRYS
GILTGSIGFAIKKGKIAGLQDFLLQVHPSQDPQNHILKEFWEETFGCSFQSSLHGTKQCSGSE
RLRDIKNPFTDVSELRISNNVYKAVYAVAHAMHSMLKCGENSETVNASCTLKSILQPKEIVKHI
QDVNFSLQSGEKVSFDERGDPAATYQLVNWQRNSAGEPVFVAVGSYDASLPNGKQFTIEEM
NITWVGASRQRPISVCSESCLPGFRQAVIKGKPICCFSCIACAAGEISNSSNSADCSPCPLEY
WSNEDHNKCVPKVIEFLSYEETMGTLLTAFSLFGAGLTLVVSFVFFWFRHTPLVKASNSELSF
LLLLSLTLCFLCSLTFIGRPTDWSCMLRHVAFGIAFALCISCILTKTITVVIAFKARTPVNTVPQC
LASHQRMGVVGGTFLQVLVCVVWLALAPPFPHKNTMYAAERIILECNLGSSIGFWVVLGYIG
LLAVLCLILAFLARKLPDNFNEAKFITFSMLIFSAVWVTFIPAYVSSPGKFTVAVEIFAILASSFGL
LFCIFAPKCYILLLKPERNTKNHMMGRD
>MF_7_1_F
RGTMLLIADLLLFFLVAVREGKPTCQTYGTKELSSFFKEGDITIGGIFSFHQSPVTASSTLELNP
GSIQCKGLDQGELQYAYTMMFAIEKINNSSELLPDVTLGYRIFDSCPSIPLSIRASLSLMNMY
KGEKSSCDKPSSVHAVIGDTTSTSTIGIARTLGPFHIPVISHSATCACLSNRREYPSFFRTIPND
IFQSKALAKLVKHFGWSWVGAIRTNNDYGNGGMATFLDSAKKEGVCVEYSVAIYRTDPRKH
FLEVVNIIKKSTSKVIVAFADGTDLDILFKELHAQSVTGLQWVGSEGWITYRQIATPMNYAVVQ
GAVGFAALSSPIPGLQEFLRNSKPSITEGNQGLVELWEMVFNCTLNYKTEAHAQDYVAPCN
GIETLRNINLPFTDVSDTSLLNNVYKATYAIAHALNLLINCENGQGPFDNKSCADRKNIQPWQ
VLYYLSRIRFTADTGENVFFDDLGDPVARYALVNWQIDETGQTRFQTIGYYDASRPEGQQFDI
NPGVSAVWAGENHEVPKSICSESCLPGTFRTFIKGKPICCFDCIPCAEGEFSNSTNAVNCHK
CHPEYKSSEERTSCDLKAIEFLTFTELMGILLVAFSIFGACLAMIVAVIFYHHRKTPMVKANNSE
LSFLLLFSLSLCFLCPLTFISRPTKWSCMLRHSTFGIIFVLCMSCVLGKTIVVLMAFNATVPGS
NVIKWFGPVQQRVSVLTFTIIQVLICIMWLTINPPYPHKNVKYYKDKVILECSHGSSLGFWAVL
GYIGVLSLLCFGLAFMARKLPNNFNEAKFITFSMLIFCAVWLTFIPAYVSSPGKFTVAVEIFAILA
SSYGMLCCIFFPKCYIILFKPEKNTKKHLMGKE
>MF_8_1_FN
MCSQKWPEQGCAFLQLLLLVSFSQSAETECTQRGDLDNPQLSKEGDILLGGIFSFHSSWIN
RENTYKQNPLPLQCTSLNFREFQFAQTMLFAIEEINNSTELLPGITLGYKVYDTCGSIARSVKV
ALNLASGSDTKTVMSKASCIKPAQVQAIMGETSSSPSSAIATVIGPFSIPLISHFSTCACLSDK
TKYPSFLRTIPSDYFQSRALAQLVKHFGWMWVGAIRTNDDYGNNGMVTFTETAEQLGICLEY
SVPFFRTDPPDKIQKIIDLIKASTSKVIVAFLSHMDMDVLIKELSNHNISGYQWVGSESWIFDS
QIAAVDQTHILDGAIGLSIPKAHVSGLKEFMLDVTPLNSSSNDLFVEFWEAMFSCEFKQSKSA

VEIHGECTGREDVAGVKNSFTDMSLMPIFNNVYKGVYAVAHALHMILKCNETCDNGVQLQP
LEILKYIQKIRFKTKEGDEVYFNENGDPAAKYEIINWQPRENGIVDFVKVGLYDASLSADKQLN
LQDESLIWAKNSLEVPVSVCSEKCPPGTRKVLQKGKPVCCYDCIRCPDGEISNSTV
LXXXXLLTAASLFGTCLTAIVAFIFFRHRKTPLVRANNSELSFLLLFSLILCFLCSLTFIGRPSEWT
CQLRHTAFAITFVLCISCVLGKTIVVLMAFKARLPGGNMMKWFGPTQQRLSVLGLTLIQVIICIL
WLTISPPFPFKNVQEFRDKIILECALGSELGFWAGLGYIGLLAVLCFISAFLARKLPDNFNEAK
FITFSMLIFCAVWITFIPAYVSSPGKFGVAVEIFAILASSFGLLICIFIPKCYVILLKPDKNTKKYMM
GKG
>MF_8_2_FP
QVPVSVCSEKCQPGTRKVFQKGKPVCCHDCATCAEGEISNNTDSITCVRCHPEFWSNERR
DACVRKQEVFLSYEEIMGALLTAASLFGTCLTAIVAFIFFRHRKTPLVRANNSELSFLLLFSLILC
FLCSLTFIGRPSEWTCQLRHTAFAITFVLCISCVLGKTIVVLMAFKARLPGGNMMKWFGPAQ
QRLSVLGLTLIQVIICILWLTISPPFPFKNVQEFRDKIILECALGSELGFWAGLGYIGLLAVLCFIS
AFLARKLPDNFNEAKFITFSMLIFCAVWITFIPAYVSSPGKFSVAVEIFAILASSFGLLICIFIPKCY
VILLKPEKNTRKYMMGKG
>MF_3_1_F
MQRLTLLHLYGLCMSRVSGCDTFAHFNMPSLSKPGDIMIGGIFPIFNKEISNTVMFEREPLGT
VCSGFDLRAFRWTQVMIFAIEEINKDPAFLPNVSLGYRILDSCASPTNALRTALTLAGGLQGM
GSPCPPAISALIAESGSSQSVVVAGALGPFQLPIISYFSTCACLSNRAKFPTFFRTIPSDYFQA
KALAALVKHFGWKWIGAIQSDNDYGRNGMQAFTEEVQKIGVCVAFVGTVLRTYSMSKILDVV
EMIKQSSVKVILAFVPEGDFYPLMKEVSRQNITGIQWIASEAWITAARPSTPEMFPAFGGTLG
FVVQKMAIPDLQPFLRKINPYTDPNAAFVKDFWEAMFGCRPVSTGIYRDSSEINKICQGNETL
MNSQDVFFKVTELRVSYNVYKAVYAIAHALHELVFCQSVDRKSARPCMNVSEIHPKEVTEYV
KRVNFSNRFGDNVFFDEDGDPPASYDIINWQLDNGKVQHVTVGQFISTQSGNYKLQIDEEKII
WMTGQMVPKSVCSDVCPTGTWKASIRGKATCCFDCIPCADGTISNSTGAADCEPCPQEYW
SNERKDECIPKKKEFLTYSEPMGIILTVLSLLGASLSLITMVVFIHFKETPVVKASNSELSCFLLF
SLFLCFICPLTFIGRPTLWTCMLRHTAFGITFALCISCVLGKTTVVVTAFKARHPGNKAARKFG
PVQQRIIVCSCTVIQVVICILWLSLSPPFPEKVFRYSNKKIILECNTGSEAAFYVVLGYIGVLAVIC
LVFAFLARKLPDNFNEAKCITFSMLIFCAVWITFIPAYVSSPGKFTVAVEIFAILSSTFGLLICIFAP
KCYIILIKPEKNTKSHVLGPI
>MF_13_1_F
MALLAANVLAFIGLFTGRFCTAGTLDSCVFLGEREELSLYEDGDVVIGGLFPLHFSPVSSPLTY
KTKPPPNSYNYFSSRALRWMHTMSFAIKEINKRNDFLSNITLGFHIRDSCDDIPVSLKATLQL
VNGQPESNLSTEGVTRNSSMEITNTLGCAAVNRRVSSVIIGDAGSGVSMALLRSLGSFHIPLV
SYFASCSCLSNQKEFPTFMRTMPSDAFQIRALIQLVRYFGWTWVGVIGVESDYARFAIQLFLQ

ESMHYGVCAAYTNFYPVELNQHALDELLDVIQMKTSKVIVNFSGESEMQDILREVQRRNITG
LQWIASEAWATAKSLWEKSGKLLQGTLGFAIQRAAVIPGLHQHLTSLSPLSIHKTAFLDEFWE
ETFKCRLNDSINTHYHGEDTYHDRKMCKGTEKFDDVYSPYFDVTQLRVSYNVYKAVYLVAH
SLQDMSDCKDGKGPFQNGSCANPKNFKPWELLHYMKRTNFSVLGKEVNFDQNGDPIAYY
DLMNWQQMSDGSLSLVKVGFYDASLPSG
NLVINDSMIQWPVGKKNFALQALQSVCSESCPPGFRVARRKGEPICCYDCIPCAEGEISNAT
DSLECSPCSEDTWPNAARDLCIPKSVEFLSYYDALGILLCTTSVLGACMSFFVVIVFFKYKDT
PIVRANNMELSFLLLVFLAICFLVGLLFIGEPSDWLCLIKFPAFGISFALCISCLLAKTSVVLMAF
RARLPGSNVMKWFGPKQQRASVLLGTAVQVFVCLSWLLTAPPHANYNTNYHSSTIIIECVTG
SEVGFWCVLGYIGFLACMCLLMAFLARKLPDNFNEAKFITFSMLIFFAVWITFIPVYMSTVGKF
TVAVHVFAILASTLGLLLCIFAPKCYILLLRPDKNCKKKS
>MF_2_1_F
MFALTPLSLFYSVNILTLTHFQCFSVTSPAPSTVRPCTLQNRFQPAFTANGDYVIGGIFPLHYN
QEMPDLNCTYRPPPVRCNGFDPRAFRWALTMKLAVEEINQSTQLLPNYTLGYKIFDSCAYPL
TGQRAAVAVLNGISEEESPTCSDASLLLAVIGESGSAQSIVVSRILQPFRIPMISYFSSCACFTD
RRSYPTFFRVIPNDDYQVKAIAQLLVRFNWTWVGVVRGDHEYGRFALQGLLRELKGTKVCVA
YQEMIPLLYNRQKALEIMQVMKSSLAKVVVVFSAEGEMTPFLRDYMIQNITGIQWVASEAWV
TASVFTGSEYYPFLGGTIGFGIRRGHISRLSDYLMTVSPENYPNNPLVKELWEALYGCSPHAP
SASQLPLCTGQEILLVQHSAYMNTSSPRVAYNVYKAVYAVAHSLHNLLLCKPGMGPFENNS
CAQSNNVLPWQLQHYIEEVKFNIAGEEVNFDLKGDSVPYYDIINWQKGTSGNIEFVNVGLFD
GTKAAGEELVIYEDKLTWAGHQREASCSHLSVCSASCPPGSRKAVRRGEPICCFDCIPCDS
GKISNETNSVECIFCPEDFWSNEDRTICIPKKVEFLAYDSLGIALTVISVVGACLTIGVFLVFFHH
RNTAVVRVNNSELSFFILFALTFCFLCSLVFIGEPTRWSCMLRHTAFSITFSLCISCILAKTLVVL
AAFTATRPGDNIMKWLGPKHQRVIIFSCTMVQVIICAAWLIDASPYPSRNTKYERSKIILECSV
GSSLAFWCVLGYIGLQACLCFVLAFLARKLPGNFNEAKFITFSILIFCAVWLAFIPAYISSPGIYA
DAVESFAILASSFGLLFCLFAPKCYIILLKPEKNTKQHLMGKE
>MF_14_1_YP
RETAETRQEVIHFRVAGVGVDSDASQCVTLGDSHPVVLQSEGDVIVGGLFPLHYEASEPEHT
YHSKPPTTPCTGFDQRAFRWLMTMVFAVEEINHSSVLLPGVTLGYRITDSCDNVHNSLRALF
PLIRNVTTYSKEQEFRTTWSETNACLSGSPVSAVIGLASSSPTRAVAQALGPFSIPLVSYFATC
TCLTDKRMYPSFLRTVPSDIFQVRGLVQLVTFLGWRWVGTIGTTDDYSQYGIQSFSAQFQKQ
GGCVAFHLTIPKSPTLAEIQEMADRVQRSAARVVVVFATEGQLLDLYFELVQRNVTHLQWVAS
EAWVTAALLTLPHFHSLLEGTLGFSFPGVNIPSLENFLLNIHPSAEPGMEFINMFWEDQFGCK
LHFEEDGSIKNVGDVNLRETPFCTGFENLSLTGSSYTDVSRVRISYNVYKAVYAIAHALHALLN
CDSAEQNEKSCDKHKQFTSEQLLSHLKLVNFTNKFGEKVCFDSNGEPVPLYDIINWQKDKH

GKMRFVTVGSYDGSVPLGRQLQLNQSNIIWTEGHTEVPVSLCSEPCPPGNRQARRKGEPQ
CCFDCFPCADGEISNETGSAECIKCPESYWSDEKKVKCVAGIEEFLSFKDSMGIILVALTLLGV
VLTAVIIVIFHRFRTTPIVKANNSEISFLLLLSLKLCFLCSLVFIGQPSVWTCRIRQTAFGISFVICL
SCLLVKTIVVLLAFQTKLPGSRALKLFGPSQQRIMIVFTTAPQVCLCAGWLLWAPPFPFRNSA
YQASTRKVRP
>MF_4_6_YP
TVVFFSFVQVLIFIIWLVLRPPFPMKNFSTYKERIILECALGSAIGFWAVLGYIGLLAVFCFVLAVL
ARKLPDNFNEAKLITFSMLIFCAVWLNYVAAYVSSPGKFTVAVEIFAILASSFGLIMCIFAPKCFII
L
QPEKNTQKYLMNKK
>MF_4_1_F
MEISAFFTILLLPLCFLEMNSVFLLSGYLTGLKQRTEDDVANIDASSVKCKLRGTTRLPAFSMD
GDFVIGGVFSIHYTMYTEIHIHATMPDPPKCIGSINARELRFSRTMIFAIEEINNSTELLPGIRLG
YQIHDSCAAVPVAMQAAFQLSNSGDKVFYKDSNCSQSGMMAAVVAESGSTPSISISRIIGPF
NIPQVSHFATCACLSDKQQYPNFFRTVPSDAFQSVALAKLVKHFGWTWIGAICSDSDYGNN
GMASFLQAAQKEGICVEFSESFHRTQPHKIQKVADFIRRSTAKVIVAFTSTGEMKVLLEELAA
KPSPSRQWIGSESWITDTNLQKFTFCEGAIGFAIPKSDIPGLRDFLLDLSLSEVAASPVLTEFW
EETFRCRLRKDVNTEQRVCNGSEDLQKVPNAFIPSSPLRITTMVYKAVYAIAHAIHSVVCVKE
NVTAQCDKHLMLESKQVLDALKKVNLTQNGYPVSFDANGDPVAVYELVNWQKGESGNIELV
TVGFYDASLPKYKEFYINKNLTWVDGIQQVGMSVCTDSCPPGTHKVLQKGKPICCYDCIPCP
EGEISNTTDSSDCFPCPIEFWPNPEKDSCHPKPVEFLSIDETLTIILAVVSVGGACLSILTAAVFF
HHRTTPIVRANNSELSFLLLFSLTLCFLCSLTFIGAPTEWSCMLRHSAFGITFVLCISCVLGKTI
VVLMAFKATLPGSNVIKWFGPLQQRLTVVFFTFVQVLICIVWLVLRPPFPMKNFSTYKERIILEC
ALGSAIGFWAVLGYIGLLAVFCFVLAVLARKLPDNFNEAKLITFSMLIFCAVWLSFIPAYVSSPG
KFTVAVEIFAILASSFGLIMCIFAPKCFIILCQPEKNTKKYLMNKN
>MF_4_2_F
MEISAFFTILLLSLCLFEMNSVFLLSGSLTGLKQRTGFDVANIDASSVKCKLRGTTSLPAFWM
DGDFVIGGVFSIHYTMYTEIHIHATMPEPPKCIGSINARELRFSRTMIFAIEEINNSTELLPGIRL
GYQIHDSCAAVPMAMQAAFQLSNGGGKVFYKDSNCSQSGMMAAVVAESGSTPSISISRIIG
PFNIPQVSHFATCACLSDKQQYPNFFRTVPSDAFQSVALAKLVKHFGWTWIGAICSDSDYGN
NGMASFLQAAHKEGICVEFSESFHRTQPHKIQKVVDFIRRSTAKVVVAFAGFGEMKFLLEELA
AKPTPSLQWVGSEAWITDTNLQRFTFCEGAIGFAIPKSVIPGLRDFLLDLSLSEVAASPVLTEF
WEETFKCKLRKDVNTEQRACNGSEDLQKVPNAFIPSSPLRITTMVYKAVYAIAHAIHSVVCVK
KNETAECDKRLMLESKQVLKALKKVNFTQNGYHVSFEENGDPVAVYELVNWQKSERGVIELV
TVGFYDASMPINRKFHFDKNLTWMDGRKQVSMSVCTDSCPPGTHKVLQKGKPSCCFDCIP

CPEGEISNTTDSSDCFPCPIEFWPNPEKDSCHPKPVEYLSIDETLAIILAAVSVGGACLAILTAA
VFFHHRTTPIVRANNSELSFLLLFSLTLCFLCSLTFIGAPTEWSCMLRHSAFGITFVLCISCVLG
KTIVVLMAFKATLPGSNVMKWFGLLQQRLTVVFLTFVQVLICIVWLVLRPPFPMKNFITYKERII
LECALGSAIGFWAVLGYIGLLAVFCFVLAVMARKLPDNFNEAKLITFSMLIFCAVWLSFIPAYVS
SPGKFTVAVEIFAILASSFGLIMCIFAPKCFVILFQPEKNTKKYLMNKN
>MF_4_3_FN
MEISAFLNILVLPLCLFEMNSVFFLSGSLIGLKQRTGVDVASADASYVRCNPWGTRHSPAFS
MDGDFVIGGVLSIHYNMYTEMYNYATMPEPLKCTGSINARALRFSRTMMFAIEEINNSTELLP
GIRLGYQIHDSCAAVPVAVQAAFQLSNREDKIFYKDSNCSQSGMMAAVVAESGSTPSISISRII
GSFNIPQVIVHIATCACLSDKQQYPNFFRTIPSDAFQSVALAKLVKHFGWTWIGAICSDSNYG
NNGMASFLQAAHKEGICVEFSESFHRTQPHKIQKVADFIRRSTAKVIVAFTATGEMKVLLEELA
AKPSPSRQWIGSESWITDTNLQKFTFCEGAIGFAIPKSDIPGLRDFLLDLSLSEVAASPVLTEF
WEETFKCRLKKDVNTEQRVCNGSEDLQKVPNAFIPSSPLRITTLVYKAVYAIAHAIHSVVCVKK
NETAECDKHLMLESKQVLDALKKVNFTKNGYPVSFDANGDPVAVYELVNWQKGESGKIELV
TVGYYDASLPINKEFHINGNLTWMDGRKQVNMSVCTDSCPPGTHKVLQKGKPSCXXXX*PL
ELTCALCVFLDSSDCFPCPIEFWPNPEKDSCHPKPVEYLSIDETLAIILAVVSVGGACLAILTAA
VFFHHRTTPIVRANNSELSFLLLFSLTLCFLCSLTFIGAPTEWSCMLRHSAFGITFVLCISCVLG
KTIVVLMAFKATLPGSNVMKWFGPLQQRLTVVFFTFVQVFICIVWLVLRPPFPMKNFSTYKEKI
ILECALGSAIGFWAVLGYIGLLAVFCFVLAVLARKLPDNFNEAKLITFSMLIFCAVWLSFIPAYVS
SPGKFTVAVEIFAILASSFGLIMCIFAPKCFIILCQPEKNTKNYLMNKK
>MF_4_7_YP
TFVLCISCVLGKTIVVLMAFKATLPGSNVMKWFGPLQQRLTVVFFTFVQVLICIVWLVLRPPFP
MKNFSTYKEKIILECALGSAIGFWAVLGYISLLAVFCFVLAVLARKLPDNFNEAKLITFSMLIFC
AVWLSFIPAYVSSPGKFTVAVEIFAILASSFGLIMCIFAPKCFIILCQPEKNTKNYLMNKK
>MF_4_8_FP
GDVNTEQRVCNGSEDLQKVPNAFIPSSPLRITTLVYKAVYAIAHAIHSVVCVKKNETAECDKHL
MLESKQVLDALKKVNFTKNGYPVSFDANGDPVAVYELVNWQKGESGNIELVTVGFYDASLPI
NKEFHIDRNLTWMDGQQPVSMSVCTDSCPPGTHKVLQKGKPICCFDCIPCPEGEISNTSDS
SDCFPCPIEFWPNPEKDSCHPKPVEYLSIDETLAIILAVVSVGGACLAILTAAVFFHHRTTPIVR
ANNSELSFLLLFSLTLCFLCSLTFIGAPTEWSCMLRHSAFGITFVLCISCVLGKTIVVLMAFKAT
LPGSNVMKWFGPPQQRLTVVFVTFVQVLICTVWLVLRPPFPMKNFSTYKEKIILECDLGSAIG
FWAVLGYIGLLAVFCFVLAVLARKLPDNFNEAKLITFSMLIFCAVWLSFIPAYVSSPGKFTVAVEI
FAILASSFGLIMCIFAPKCFIILCQPEKNTKKYLMNKN
>MF_4_4_F
MATSPVFTILVLGLSLFERNTVFVLSESMTNDPPVRCIPLATASSPAFSIDGDFIIGGVFSIYYTM

HTEIYNYTILPGPPWCTGSINSRELRFSRAMIFAIEEINNSRELLPGIKLGYQIHDKCAAVPVAM
YAAFQLISGKNTVFYKDKNCSQSGMVAAVIGGSGSTSSIGISRITGPFNIPQVSHFSSCACLS
DKQQYPNFFRTIPSDAFQAVALAKLVKHFGWTWIGAVCSDSDYGNNGMASFLQAAQKEGIC
VEFSESFYRTNPYNEIQRVADVIRRATAKVIVAFTASGEMKVLLEELARKPSPSRQWIGSESWI
TDPNLLKFDFCEGGIGFGIPKSVIPGLRDFLLDLSPSEVAASPLLTEFWENAFKCRLRKNVGA
EERVCDGSEDITKLQSPYTETSQLRVTNMVYKAVYAIAHAIHSVVCPKKNLTTKCDKHLMLES
KQVLDALKKVNFTKNGYPVSFDANGDPVAVYELVNWQKGESGNIELVTVGYYDASLPINKEF
HINGNLTWMDGRKQVNMSVCTDSCPPGTHKVLQKGKPICCYDCIPCPEGEISNTSDSSDCF
PCPIEFWPNPEKDSCHPKPVEYLSIDETLAIILAAVSVGGACLAILTAAVFFHHRTTPIVRANNS
ELSFLLLFSLTLCFLCSLTFIGAPTEWSCMLRHSAFGITFVLCISCVLGKTIVVLMAFKATLPXX
NVMKWFGPLQQRLTVVFFTFVQVLICMVWLVLRPPFPMKNFATYKERIILECALGSAIGFWAV
LGYISLLAVFCFVLAVLARKLPDNFNEAKLITFSMLIFCAVWLSFIPAYVSSPGKFTVAVEIFAILA
SSFGLIMCIFAPKCFIILCQPEKNTKKYLMNKN
>MF_4_5_F
MEISAFCTLLLLAVALLEINSVCVLCGLLTSFKQRAGLSESGVTISTDSSSVKCYLQGTAGSPV
FSMEGDFVLGGVFSIHFNMDAKIHPYTAMPEPPRCTGGIDARELRFSRAMIFAIEEINNSTELL
PGIRLGYQIHDSCGVVPVAMHKAFQLSNGGDSIFYTDNNCSQSGMMAAVVAESGSTPSISM
SRIIGSFNIPQVSHYATCACLSDKQQYPNFFRTIPSDAFQAVALAKLVKHFGWTWIGAVCSDS
DYGNNGMASFLQAAQKEGICVEFSESFYRTNPYSKIQRVADVIRRATAKVIVAFTASGEMKVL
LEELARKPSPSRQWIGSESWITDPNMMKFDFCEGGIGFGIPKSVIPGLRDFLLDLSPSEVAAS
PLLTEFWENAFKCRLRKDVGAEERVCDGSEDMTKLQSPYTETSQLRVTNMVYKAVYAIAHAI
HSVVCPEKNSTTKCDKHLMLESKQVLDALKKVNFTKNGYPVSFDTNGDPVAVYELVNWQK
GESGNIELVTVGYYDASLPINKEFHINGNLTWMDGRKQVNMSVCTDSCPPGTHKVLQKGKP
SCCYDCIPCPEGEISNTSDSSDCFPCPIEFWPNPEKDSCHPKPVEFLSIDETLAIILAVVSVGG
ACLAILTAAVFFHHRTTPIVRANNSELSFLLLFSLTLCFLCSLTFIGAPTEWSCMLRHSAFGITFV
LCISCVLGKTIVVLMAFKATLPGSNVIKWFGPLQQRLTVVFFTFVQVLICMVWLVLRPPFPMK
NFSTYKEKIILECALGSAIGFWAVLGYIGLLAVFCFVLAVLARKLPDNFNEAKLITFSMLIFCAV
WLSFIPAYVSSPGKFTVAVEIFAILASSFGLIMCIFAPKCFIILCQPEKNTKNYLMNKK
>MF_16_1_F
MGTFLFPSFCAFVVCLSCCFSSDSSSIHSSCKLGRQFHLNALHMPGDVILGGLFEVHFTSVF
PELIFTSEPTKISCQGFDPSGFRHAMTMAFAIYEVNENPNLLPNLTLGYSLYDNCATLVIGFSA
AMSLFNGQDEEFMPEENCSGRPPVLGIVGDSFSTFTIATSDVISLFRLPIVSYYATCLCLSDRK
RFPSFFRTIPSDAFQVHAMLQILKRFGWTWVGLLVSDDDYGHYVAQSFQSELHYSIGGCLAY
LETLPWGDNPVELRRIVGLMKKSTARVVIVFAHQIHMIQLMEEVVNQNVTGLQWMASEAWT
SANVLQTPRFMPYLGGTLGIAIRRGEIPGLRDFLLRIRPHDKIDDENSMVKQFWEHTFQCRF

APPPVEWMEAGGALCSGEEDLQTVETTFLDVSNLRPEYNIYKAVYALVYALDQMLRCEPGR
GPFSEHSCASLQQLEPWQLMHYLEKVNFTTSFGDEVSFDKNGDALPIYDVMNWVWLPGG
KTEVQNVGVVKKTPMKAEELMINEDKIFWNFDTVKLFDFVPRSVCSESCPPGTRIVRRKGQP
LCCFDCVPCSEGKITNQTDSIECLSCPEDFWSSKQNDHCVPKEIEFLSYDDPLGICLTTASLL
GTFICSIVFAIFIYHRSTPVVRANNSELSYLILVSLKLCFLCSLLFIGQPQLWTCQLRHAVFGISF
VLCVSCILVKTMVVLAVFRSSKPGGGSKLKWFGSVQQRGTVLALTCVQAVVCTVWIISASPTP
HANTRYHNDKIIYECVVGSTAGFAVLLGYISLLAIISFLLAFLARNLPDNFNEAKFITFSMLIFCA
VWVAFVPAYVNSPGKYADAVEVFAILASSFGLLVALFGPKCYIILLKPEKNTRKAIMGRD
>MF_16_2_F
MTIYLPTYFISTVYFITLYPEFSSLLTCNLRKNFTLNEIHKPGDVVLGGLFEVHYTSVLPERAFTS
EPQQPLCKGFDTLGFRHAMTMAFAIDEINKKINLLPNVTLGYRIYDNCGALVVGFSGALSLAS
GTDEQFKLLENCSGTPPVLGIVGDHYSTFSIAIANVLGLYKMPLVSYFATCSCLSDRQRYPTF
FRTIPSDAFQVRAMVKILKHFGWTWVGLLVSDDDYGLHVAQSFQSDLARSGDGCLAYLKVL
PWNSDQSELTQIVHLIKASTARVVIVFAHEIHMIQLMEEVVRQNVTGRQWIASETWTTAVVLQV
PHLMPYLSGTLGIAIRRGEIPGLRDFLSQIKPVKNSSGNDLVRQFWEHTFKCKFDGAGSVET
EKPLCTGMENIDAVQTEFLDLSNLRPEYNVYKAVYSLAYALHDMLKCQSESGPFSCATLSTL
EPWQLAHYLQKVNFTTPFGDQVSFDKNGDALPIYDVMNWLWLPDGKTKVQNVGEVKSAG
SGGEELYIDVDKIFWNTESKQPPQSVCSESCPPGTRVARRKGEPECCFSCVSCSEGKISNTT
NSLECITCPEDFWSNSRRDLCVPKKTEFLSYHEPLGICLTTTSLLGTFICTVVLAIFAFHRRSPI
VRANNSELSFLLLVALKLCFLCSLLFIGQPRLWTCQLRHAVFGISFVLCISCILVKTMVVLAVFK
ASEPGGGGNLKWFGPKQQRGTVFSLTCIQAAICAAWLVSSSPAPRKNTQYHNDKIVYECEV
GSTIGFAVILGYIGMLAILSFFLAFLARNLPNNFNEAKLITFSMLIFCAVWMAFVPAYINSPGRY
ADAVEVFAILASSFGLLVPLFGPKCYIILLKPEKNTKKAIMGR
>MF_15_1_F
MSWGYRLVTLWTSHAPSLLLVGLLGRQLGLQAGFQVVKTTNCSRWGTHSDNGLFEDGDIII
GGLFSLRYTPPSVDYSFKQQPDNKPCTGVQNLPLQYIYAMLYALEEINRSLTLLPGVKLGYQI
HDSCALPAWELKAVLSMVGGNTFTCDTEAGNGMDIGQGDKPIPLIIGGSNSVTAQILSRVLG
PLSVPVVSYFLLCPCLSDRQQYPNFFRTMASDAYQARAVAQLAIHFNWTWIGAVIADNDYGH
MAVKVFQESIQENSVCLAFVETLQRETIVSDAKRAALTIQASTAKVILIFTWYTDVIELFHQLSKL
NVTDRQFLASEAWSTSGDLLQDTASHKVARGVLGVAIRSTALPGFDSYIRSLSPLLRPSDEFL
REFWQKEFGCDPNTNGPTQKASLPPCKGTETLEEVQNFFTDTSQLRVTYNVYLAVYAAAHA
LHSLLSCPERANSPGNVSSCSFPKEIKPFELMQHLSQVNFTTPQDELFYFKGGDITAKYDLV
NWQSTPQGGLRLALIGRVDGFDLHLNDTAIEWSTGTSQVPVSVCSESCAPGTRKASRKGEP
ICCFDCILCADGEISNQTGSLQCDHCPAEFWSNTERTVCVPRQLDFLSFNETLGVTLTAVAVS
GATVTTAVFVVFLHHRQTPVVRANNSELSFLLLLSLKLCFLCSLLFIGRPSYWACRFQQAAFGI

SFVLCVSCLQVKTIVVLAAFRSARPGAEALMKWFGPGQQRGSVCFFTSIQVVICIIWLTLSPPI
PQPDLEIPGLKVTLKCAMASVVGFSVVLGYIGLLGCTSLLLAFLARKLPDNFNEAKLITFSMLI
FCAVWVAFVPAYVSSPGKYAVAVEVFAILASSFGLLFCIFAPKCFIILLRPEKNTKKHLMAR
>MF_16_3_YP
PSVCAFVVCLSCCFSSDSSSIHSSCKLGRQFQLNALHMPGDVTLGGLFEVHFTSVFPELIFT
SEPTKISCQGFDPLGFRHAMTMAFAIHEINKNPNLLPNLTLXNAAMSLFNGQDEEFMLQENC
SGRPPVLGIVGDPFSTFTIAASDVISLFRLPIVSYYATCLCLSDRKRFPSFFRTIPSDAFQVHAM
LQILKHFSWTWVGLLFSDDDYGHHVAQSFQSELHHSVGGCLAYLEMLPWGDNPVELKRIV
GLMKKSTARVVIVFAHQIHMIHLLEEVVNQNVTGLQWMASEAWTSANVFETPRFMPYLGGT
LGIAIRRGEISGLRDFLLRIRPHDKIDDENSMVKQFWEHTFQCRFAPPPVEWMEAGGALCSG
EEDLQTVETTFLDVSNLRPEYNIYKAVYALVYALDQMLRCEPGRGPFSEHSCASLQQLEPWQ
LMHYLEKVNFTTSFGDEVSFDKNGDALPIYDVMNWVWLPGKIEVQNVGFFKKTTLNDEELVI
DEDKIFWNFDKVPRSVCSESCPPGTRMVRRKGQPLCCFDCVPCSEGKITNQTDSIECLSCP
EDFWSSKQNDLCVPKKIEFLSYNDPLGICLTTASLLGTFICSIVFAIFIYHRSTPVVRANNSELS
FLILVSLKLCFLCSLLFIGQPQLWTCQLRHAVFGISFVLCVSCILVKTMVVLAVFSSSKPGGGS
KLKWFGSVQQRGTVLALTCVQAVVCTVWIISASPTPHANTRYHNDKIIYECVVGSTAGFAVLX
XXQMRL
>MF_16_4_YP
GTVLALTCVQAVVCTVWIISASPTPHANTRYHNDKIIYECVVGSTAGFAVLLGYISLLAIISFLLA
FLARNLPDNFNEAKFITFSMLIFCAVWVAFVPAYVNSPGKYADAVEVFAILASSFGLLVALFGP
KCYIILLKPEKNTRKAIMGRD
>MF_10_5_FP
DSADCSPCPLEYWSNEDHNKCVPKVIEFLSYEETMGTLLTAFSLFGAGLTLVVSFVFFWFRH
TPLVKASNSELSFLLLLSLTLCFLCSLTFIGRPTDWSCMLRHVAFGIAFALCISCILTKTITVVIAF
KARTPVNTVPQCLASHQRMGVVGGTFLQVLVCVVWLALAPPFPHKNTMYAAERIILECNLG
SSIGFWVVLGYIGLLAVLCLILAFLARKLPDNFNEAKFITFSMLIFSAVWVTFIPAYVSSPGKFTV
AVEIFAILASSFGLLFCIFAPKCYILLLKPERNTKNHMMGRD
>MF_8_3_YP
STDSITCVRCHPEFWSNERRDACVRKQEVFLSYEEIMGALLTAASLFGTCLTAIVAFIFFRHRK
TPLVRANNSELSFLLLFSLILCFLCSLTFIGRPSEWTCQLRHTAFAITFVLCISCVLGKTIVVLMA
FKARLPGGNMMKWFGPAQQRLSVLGLTLIQVIICILWLTISPPFPFKNVQEFRDKIILECALGS
ELGFWAGLGYIGLLAVLCFISAFLARKLPDNFNEAKFITFSMLIFCAVWITFIPAYVSSPG
>MF_4_9_FP
PCPIEFWPNPEKDSCHPKPVEFLSIDETLTIILAVVSVGGACLSILTAAVFFHHRTTPIVRANNSE
LSFLLLFSLTLCFLCSLTFIGAPTEWSCMLRHSAFGITFVLCISCVLGKTIVVLMAFKATLPGSN

VIKWFGPLQQRLTVVFFTFVQVLICIVWLVLRPPFPMKNFSTYKEKIILECALGSAIGFWAVLGY
IGLLAVFCFVLAVLARKLPDNFNEAKLITFSMLIFCAVWLSFIPAYVSSPGKFTVAVEIFAILASSF
GLIMCIFAPKCFIILCQPEKNTKK
>MF_4_10_YP
KVPVSVCTDSCPPGTHKVLQKGKPSCCFDCIPCPEGEISNTTDSSDCFPCPIEFWPNPEKD
SCHPKPVEYLSIDETLAIILAAVSVGGACLAILTAAVFFHHRTTPIVRANNSELSFLLLFSLTLCFL
CSLTFIGAPTEWSCMLRHSAFGITFVLCISCVLGKTIVVLMAFKATLPGSNVMKWFGPPQQR
LTVVFLTFVQVLICIVWLVLRPPFPNENFITYK
>MF_4_11_YP
GKPICCYDCIPCPEGEISNTSDSSDCFPCPIEFWPNPEKDSCHPKPVEYLSIDETLAIILAAVS
VGGACLAILTAAVFFHHRTTPIVRANNSELSFLLLFSLTLCFLCSLTFIGAPTEWSCMLRHSAF
GITFVLCISCVLGKTIVVLMAFKATLPGSNVMKWFGPLQQRLTVVFFTFVQVLICMVWLVLRP
PFPMKNFATYKERIILECALGS
>MF_4_12_YP
SFLLLFSLTLCFLCSLTFIGAPTEWSCMLRHSAFGITFVLCISCVLGKTIVVLMAFKATLPGSNV
IKWFGPLQQRLTVVFFTFVQVLICMVWLVLRPPFPMKNFSTYKEKIILECALGSAIGFWAVLGY
IGLLAVFCFVLAVLARKLPDNFNEAKLITFSMLIFCAVWLSFIPAYVSSPGKFTVAVEIFAILASSF
GLIMCIFAPKCFIILCQPEK
TKNYLMNKK
>MF_16_5_YP
NNSELSFLILVSLKLCFLCSLLFIGQPQLWTCQLRHAVFGISFVLCVSCILVKTMVVLAVFSSSK
PGGGSKLKWFGSVQQRGTVLALTCVQAVVCTVWIISASPTPHANTRYHNDKIIYECVVGSTA
GFAVLLGYISLLAIISFLLAFLARNLPDNFNEAKFITFSMLIFCAVWVAFVPAYVNSPGKYADAVE
VFAILASSFGLLVALFGPKCYIILLKPEK
>MF_8_4_YP
PSEWTCQLRHTAFAITFVLCISCVLGKTIVVLMAFKARLPGGNMMKWFGPAQQRLSVLGLTLI
QVIICILWLTISPPFPFKNVQEFRDKIILECALGSELGFWAGLGYIGLLAVLCFISAFLARKLPDN
FNEAKFITFSMLIFCAVWITFIPAYVSSPGKFSVAVEIFAILASSFGLLICIFIPKCYVILLKPEKNTR
KYMMGKG
>MF_10_6_YP
ASHQRMGVVGGTFLQVLVCVVWLALAPPFPHKNTMYAAERIILECNLGSSIGFWVVLGYIGL
LAVLCLILAFLARKLPDNFNEAKFITFSMLIFSAVWVTFIPAYVSSPGKFTVAVEIFAILASSFGLL
FCIFAPKCYILLLKPERNTKNHIMGRD
>MF_4_13_YP
SCMLRHSAFGITFVLCISCVLGKTIVVLMAFKATLPGSNVMKWFGPPQQRLTVVFFTFVQVFI

CIVWLVLRPPFPMKNFSTYKEKIILECALGSAIGFWAVLGYIGLLAVFCFVLAVLARKLPDNFN
EAKLITFSMLIFCAVWLSFIPAYVSSPGKFTVAVEIFAILASSFGLIMCIFAPKCFIILCQPEKNTK
NYLMNKK
>MF_4_14_YP
FKATLPGSNVIKWFGPLQQRLTVVFFTFVQVLICIVWLVLRPPFPMKNFSTYKERIILECALGSA
IGFWAVLGYIGLLAVFCFVLAVLARKLPDNFNEAKLITFSMLIFCAVWLSFIPAYVSSPGKFTVA
VEIFAILASSFGLIMCIFAPKCFIILCQPEKNTKKYLMNKN
