# Supplementary material for: Evolution and origin of vomeronasal-type odorant receptor gene repertoire in fishes
Source: BMC Evol Biol. 2006 Oct 3;6:76. doi: 10.1186/1471-2148-6-76 (PMC1601972; doi:10.1186/1471-2148-6-76)
Supplement: Additional File 9 — Deduced amino acid sequences of fugu V2Rs. [file 1471-2148-6-76-S9.pdf]

>Tr_5_24_F
MVGFIINVHITLFFLILKIGRDTVASTSLPAATAPGDIIIGGIFPIHEDVDKETESFEPHIRPCIRFQ
QSGFVLALAMINAIEDMNKSPPLADANITLGYRILDSCSDVSTALRATNDLMQQGNCNSSGS
SSSCGQPIMAVVGASYSETSIAIARQLTLPMIPQISYSSSAVLLSDKTHFPAFMRTIPNDKYQT
TAMITLLSHYGWNWVGIITTDGSYGLSALDQFVSQASAKGICVAFKSILPQSVSSQDTSSAIT
KTARTIYKNPKVQVIISFAKPSQMKFLFHKLKSMMLKPGETNGEGRMRRVWVASDSWSTSR
YIYGNLTLEDIGYVLGFTFKSGNVSSFREYLEQLGAPEENIKINPFLQEFYMHMNATAVGSGE
DKHVPEALRSLWEHVHADLIFSVEMAVSAITQAVATICRRTDCKTLGSVQPWQVLAALWMQE
FKLRQKSYKFDSSGDINMGYEIVMWTSVTSEISVHHVVAEYHPLYSNITFMEQHNFTTKQLL
DDLKVVSKCSNSCIPGQFKKTSEGQHTCCYECINCTENYYSNSTDMDQCLSCDADTEWSP
KGSSSCISKEQLFFSWNDIFAVVLLAFSALGILLCLLTSALFLYQRDTPVVKAAGGPLSQAILFS
LVVSYISAMLFVGEPSSLQCKARQVLFGISFTLCVSCILVKTLQILLAFQFNPALQNMLRKIYQP
YAIITICVALQTATCICWLVLLSPYAHIIKQPTTLLQYCHEGSYVAFGVMLGYIAILAFVCFICAFK
GRKLPEQYNEAKFITFSMLLYLISWLLFVPIYVTTSGVYLPAVEMVVILISNYGILSCHFFPKCYII
FFKKEQNTRSAFRKN
>Tr_V2R2_F
MDIGPFLLAFCLFPLHVSADSTCKLKAKFNLSGYKNVEKKKVVIGGMFPVHMRVSSSGGNT
SRLPVSSGCEGFNLRTFRWTRTMLFAIDEINKREDLLPDTDLGYVIYDSCFTISKAVEGTLTYL
TGQDEAVPNYRCGNGPPLAALVGAGGSDLSIATARILGLYHFPQVSYSSTCSALKSKFQFPT
FLRTIPNDQHQSTAMAKLVIHFGWTWVGTISADDDYGKYGIKDFKEQVEEAGVCISFSETLPK
VNSPENIQRIIQTLVKSTAKIIVVFSSDVDLSPLITELLRHNITNRTWIASEAWVTSALMLKPGAS
LLLGGTLGFAVKRASIPGLQHYLLDLDPYGDTLSEEFWETVHRGLPDGLCTGLETVAQLKNT
YSDVSQLRITYSVYKAVYAVAHALHNLEHCEQGRGPFNGRDCTNISSFEPWQLMYYLKNVR
YTVPHTGEEIYFDDGDVEGFYDIINWQFNSDGEISYVKVGHFNATAVPEEQMTIMNGSIVWNS
DALEPPRSVCSDNCQPGTRKGIRQGEPVCCFDCIPCADGEISNTTNARECILCGEDDWSNE
AHDACVPKIIEFLAFGEPLGITLIVISAFGALVTIAVGVVFIVNVGTPLVEANDAVLSLSLLFSLVVT
FLCSIVFLGEPQNWSCMTSQVALALGFALCLSCIMKGARRCGHINTGKCS
ISQQQLLNFTS
CSFVLQAVACTVWLILLPPHAIKNTSAQNIKIILECDEGSIVFICCVFAYDILLALIAFIFAFMARKL
EDHFSEGKCMTFGMLVFFIVWISFVPAYLSTRGKFMVAVQIFAILASSFGLLTCIFLPKCYILLIK
PERNKEDMMRP
>Tr_2_1_F
MTLLSDIILFYSFFVVPVLSDPMSSVTSPSSSLCRLQNSFQPGFEANGDFIIGGMFPLHFNQE
MPDLNNTYRPPPVKCNGFDPRAFRWAQTMKLAVEEINQRTDLLPDHVLGYKLFDSCGYPLT
GQRAALSLLNGPSTDGSSTCTGAAPLLAVIGESSSSLSVMLSRILQPFKIPVISYFSSCACLS

DKRKYPNFFRVIPNDDYQVKAIAQLLVHFNWTWVGLLLCERDYGRFAAEGLLRELKSTKVCV
AYQEIIPLVYNQLRVQAIMQVMRTSSAKVVVVFSAEVEMIPLLRDYMKQNITGIQWIASEAWVT
ASVFTGSKYYPYLGGTIGLGIRKGHIPRLSDYLLTVNPQVYPNNPLVKELWETLYGCRSSLSS
SSHVPLCTGQEPLLEQHSAFMNTSSPRVTYNVYKAVYAIAHSLHNLFLCQPGPGPFKNNTC
AQRNNVQPWQLQHSLQEVAFKISGEVVDFDQRGDSIPYYDIINWQRGVDGSIEFVNVGLFD
GSKPTGKELVIQEDRIVWAGHQREASLSICSASCLPGTRKAIRKEVPVCCFDCIPCDTGKISN
ETDSVDCTFCPEDFWPNPNRTVCIPKKVEYLAYDSLGIALMVTSVVGACATIATFAVFFYHRN
TAIVRVNNAELSFFILLSLVLCFLCSLVFIGEPTTWSCMLRHTAFSITFSLCFSCILGKTLVVLAAF
TATRPGHNIMKWLGPKQQRTIIFSCTLVQVVICTAWLIDAPPAPFRNTEYEHSKIILECSVGSSV
GFWCVLGYIGLQACLSFILAFLARKLPGNFNEAKFITFSMLIFCAVWLAFIPAYVSSPGNYSDA
VESFAILASSFGLLFCMFAPKCYIILLKPERNTKQHLMGKE
>Tr_6_1_F
MARTTVVLFIYCLLLMRTMSKTQTCTVTGQTGFMEFLKEGDLIIGGVFSMSSTRVLVDNDYM
AIPSAYCTRWNDRELKFARTAIFTVEEINRDGKLLPGVTLGYRLYNGCGSENLIRAAVEAVTGE
GCSSQVQALLGHSSSGVSEDINLILSPFSIPQVSHLSTCACLSDKKKYPTFFRTVPSDHFQIS
GLVQLLKFFDWRWVGIVYATGSYSDDGTAHFVKEAEKEGICVEYRLCFSIASGEKSTAIVKAL
QESSSRVVLLFMSMFKTKAFLNEMENNNITDKQWLGSESWITQADLASSKRQHILQGAIGF
ALPQMVIPGLGDFLLSLKPSDEPQSDIVKAMWGDFFNCSFSPSSTSAACTGTEDLRTVTND
YTEVTHFRAENNVYKAVYLVAYALQGLLQCENGSNPTTGKPCVNKKEVQPKLVLEHIKYVNF
TTKYGSKVFFDKNENSVAQYDLVNWQMREDGSVDIVTIGQYDTSFKGEELKVADDAKIVWG
GKYKQVPRSVCREPCPPGTRKAINKLKPVCCFDCFECPDGTFSNQTDSLDCFSCPQELWP
NDQKNLCLPKPSEYLSYREITGALLSGFGCLGVFLSLLTTLIFLVHKETPIVKANNSELSFLLLL
SLKLCFLCSLTFIGRPSEWSCMLRHTAFGITFVLCISCVLGKTVVVLMAFRATLPGSKVMKWF
GPKQQRLTVLTFTLIQVVICILWLTTNPPFPMRNMTYYKDKIILECALGSAVGFWAVLSYIGVLA
ILCFILAFLARKLPDTFNEAKLITFSMLIFCAVWITFIPAYISSPGKFTVAVEIFAILASSFGLLLCIF
FPKCYIIIFRPQKNCRKSLLGNP
>Tr_8_2_F
MTSTQNWPEQGWALLQLLLLVSFSQAEDRVCLQMGDPENPQLSKDGDIILGGIFSFHSSWI
NRRDTYMHKPLPLQCISLNFRGFQYAQAMLFAIDEINNSSDLLPGITLGCKLYDSCRSIARGV
RASLALINGGETTFKLSDECTKPAQVQAIMGETSSSPNMAVATVIGPFHVPLISHFATCACLSD
KNKYPSFLRTIPSDHYQSRALAQLVKYFGWTWVGAVRSNDDYGNNGIATFIETAEELGICVEY
SVAVFRTDPPKKIQQIIDIIKASTSRVIVGFLSHLDIDVLILEMSQHNLTGYQWVGSEGWIIDSHT
AAMDVRHILDGAVGLSIPKAHVTGMKEFILDVKQLNSSSKELFREFWEALFDCKFEDSASTTT
ENQRECSGHEDLAGVKNTFTDMSLMPIFYNVYKEVYAVAHTLHDILSCNNTCNKTAQLDPFT
ILQHIKKIRFKTKEGDEVYFNENGDPPAKYEIINWQPTENGNVEFVPVGLYDASLPADRQLTLT

NRTFVWTQNSEQVPVSVCSEKCPPGTRKVLQKGKPVCCYDCLRCAEGEISNSTDSISCVRC
HSDFWSNERRDTCIKKKEEFLSYEEMMGALLTAASLLGTCLTAVVMFIFFRYRRTPIVRANNS
ELSFLLLFSLTLCFLCSLSFIGRPSGWSCMLRHAAFGITFVLCISCVLGKTMVVLMAFRATLP
GSNVMKWFGPAQQRLSVLGFTLIQVFICLLWLTISPPFPFKNIKDFKDRIILECALGSALGFWA
VLGYIGLLAMFCFILAFLARKLPDNFNEAKFITFSMLIFCAVWVTFIPAYVSSPGKFSVAVEIFAIL
SSGFGLLICIFIPKCYIILLKPDRNNKKNIMGKA
>Tr_6_2_F
MQLLRLLLLAVLLGKGTPLCRLRDSAQVPELAQDGDFVIGGIFSFRTGQDYVIDTFQHIPEAR
KCKNFNYREFKFAQTLIFAVEEINRNPDLLPHLKLGYKIYNTCGTMDILRAALALVSGLENEIS
NENCTKIKTIQAILGHSGSRPTIAFAQVVGRFHIPVISHFATCACLSNRKEFPTFFRTIPSDFYQ
SRALAKLVKHFGWTWIGAIAVDNEYGLSGIATFIQAAQEHGVCTEYSESFSSSGPPETLQRIV
EVVKRATSKVIVAFMSHREIKLLAQELYSQNITGLQWVGSDAWITDHSLTDSSGHSILLGSLGF
TVSKAKILGLEEHLRGLHPAQFPTSAFMTEFWEDRFACSLNITGSTGRRPCSGSESLQNDTS
PFTDVTELRFTNNVYKSVYVVAHALDNLMKCEKGEGPFSEGSCADPKHIQPWQVLHYINTV
RFNTSEGETVYFERSGDSPARYELVNIQLTDKGTLEGRTVGIYDASLPEDHQFIMSDEPIVWG
NGEMEAPVSVCTQRCLPGTHKVLQKGKPVCCFDCVPCPPGEISNSTNSIHCIKCPNLFWSN
QERDACIPKSIEFLAHKELLGTLLVLFSLLGVFLTTFMFLIFYCHKETPIVRANNSELSFLLLFSL
TLCFLCSLTFIGRPSGWSCMLRHTAFGITFVLCISCVLGKTMVVLMAFRATLPGSNMLKWFG
PAQQRLCVVAFTFVQVLICILWLTISPPYPFENMDYYNDRIILECALGSAVGFWAVLGYIGLLA
MFCFILAFLARKLPNNFNEAKFITFSMLIFCAVWVTFIPAYVSSPGKFLVAVEIFAILASSYGLLF
CIFLPKCFIILFRPELNTKKHIIGKT
>Tr_4_2_Y
SVIFLCFISLFDLNSAGDLKAPENSLKQQVGPREDTTGAAAPSDICRLQGSARLPAFSKDGDF
VIGGVFSIHRYTVTVNHNYTTMPEPFRCRVIDHHELQLSHAMVFAIEEINNSTELLPGIKLGYQI
HDSCAAVPIAVNVAFQLLNTLDPVFVTGDNCSQSGMVMAVVGESGSTPSISISRVIGSFDIPLV
SHFATCACLSDKQKYPSFFRTIPSDQFQADALAKLIKHFGWTWIGTVCSDSDYGNNGMAAF
LHAAQKEGICVEYSESFYRTHPHSRIKRVADVIRRSGATLSCCGIYHVGDLRILLE
LSRGPSTPRQWIGSDXW
TDSDMLRFSFCAETIGFAIQRSVIPGLRDFLLDLSPSKVASSRVLTEFWEDSFNCRLGKVAVA
GERMCDGSEDIMTLQSPYTDTSELRITNMVYKAVYAIAHAIHNAVCQDTNATTRCGKFTTINP
KQVLAQLKTVNFSQNGYAVSFDANGDPVASYELINWKKSGSGSIEVVPVGYYDASLPEGQE
FRIFRDITWVDGRKQVPVSVCSDSCPQGTRKVLQKGKPICCYDCVQCPEGEISNVTDSPECI
PCLDDFWPNPERNACFPKPVEFLSFNEVLGIILAVFSVGGACLAVITAAVFFHHRTSPIVRANN
SELSFLLLFSLTLCFLCSLTFIGAPSHLSCMLRHTAFGITFVLCI
CILGKICGGVN

>Tr_14_1_F
QHIAKEKNDTHGLEAGDGNDASPSLPRCVKTVDTQRPALHSRGDVMIGGIFPLHYSASVSQ
QKYINKPELITCSGFDHRAFRWMMTMVFAVNEINNNSSLLPGVKLGYRILDGCDHVPTSLQA
LLSLVKESMTWKQVEEMIPACLADSPVAAVIGLASSSPTGAAAHILGSFNIPLVSYFATCTCLS
DKSTYPSFLRTVPSDLFQVRGLVQMVTFMSWLWVGTIGTTDDYSHYGIQAFSHQLRQQGG
CVEFQLTIPKSPTAAELKEMADRLQSSTARVVVVFATEGQLLE
FSWRNMTGIQWVASEAWVTASLLTTPRFHALLEGTLGFSFPGAEIPGLKEFLLNICPSPKPG
MEFVNMFWEDLFGCKLNSDTKSADESPVCTGSEDLRYTQSSYTDVSQVRISYNVYKAVYAI
AHALHSLLNCESPGSNVGTCKKREPFTSKQLLQHLKSVNFTNQFKEKVYFDAKGEPVPLYDI
INWQKDSVSNIRFVKVGSYDGSAPLKQQLQMDQNAIVWTGGKSKVPVSLCAAPCPPGSRQ
ARRPGQPHCCFDCLPCADGQISNQTGSTECQRCPEYYWPDKDKVKCLPGIEEFLSFSETM
GIILVILTLVGVLLTFSLTIIFLHFRSTPIVKANNSEISFLLLLSLKLCFLCSLLFIGQPSLWKCRLRQ
AAFGISFVLCLSCLLVKTIVVLFAFRTNLRAGCCALKLFGPSRQRTLILCTTAPQVCLCAGWLL
AAPSFPVRNPAYQALTGKIVLECKEPWPPGFYLVLGYIGLLAFLCLLLAFVGRKLPNTFNEAKL
ITFSLLIFWAVWISFIPAYVSSPGKFTVAVEVFAILASSFGLLLCIFLPKCFIILLRPERNVKKGMT
GKC
>Tr_4_3_FP
GTRKVLQKGKPICCYDCVQCPEGEISNVTDSSDCEPCSEEFWPNPERNACFLKPVEFLSFN
EVLGIILAVFSVGGACLAVITAAVFFHHRASPIVRANNSELSFLLLFSLTLCFLCSLTFIGAPSQL
SCMLRHTAFGITFVLCISCILGKTVVVLMAFRATLPGSNVMKWFGPPQQRMTVVTFTSIQVLI
CIVWLVVSPPFPVRNLTTYKERIILECALGSSVGFWAVLGYIGLLAAVCLVLAVLARKLPDNFNE
AKMITFSMLIFCAVWITFIPAYVSSPGKFTVAVEIFAILASSFGLILCIFAPKCFIILFKPEKNSKKH
LMNKK
>Tr_4_4_FP
HDCCAAVPIAVHVAFQLLNTLDPVFVTGDNCSQSGMVMAVVAESGSTPSISISRVIGSFDIPLV
SHFATCACLSDKQKYPSFFRTIPSDQFQADALAKLIKHFGWTWIGTVCSDSDYGNNGMAAF
LHAAQKEGICVEYSESFYRTHPHSRIKRVADVIRRSTAIIVVAFTSSGDLRILLEELSREPSPPR
QWIGSESWVTDSEILRFSFCAGTIGFAIQRSVIPGLRDFLLDLSPSKVASSPVLTEFWEDSFNC
RLGKVAVAGERMCDGSEDIMTLQSPYTDTSELRITNMVYKAVYAIAHAIHNAVCQDTNATTR
CSKFTTINPKKVLAQLKTVNFSQNGYAVSFDANGDPVASYELVNWKKSGSGSIEVVPVGYYD
ASLPEGQEFRIFRDITWVDGRKQVPVSVCSDSCPQGTRKVLQKGKPICCYDCVQCPEGEIS
NVTDSPECIPCLDDFWPNPERNACFLKPVEFLSFNEVLGIILAVFSVGGACLAVITAAVFFHHR
TSPIVRANNSELSFLLLFSLTLCFLCSLTFIGAPSHLSCMLRHTAFGITFVLCISCVLGKTVVVL
MAFRATLPGSNVMKWFGPPQQRMTVVTFTSIQVLICIVWLVVSPPFPVRNLTTYKERIILECAL
GSSVGFWAVLGYIGLLAAVCLVLAVLARKLPDNFNEAKMITFSMLIFCAVWITFIPAYVSSPGK

FTVAVEIFAILASSFGLILCIFAPKCFIILFKPEKNSKKHLMNKK

>Tr_16_4_FP

VCCFDCLP

SEGEISNTTDSMECTSCPEDFWSSPQRDHCVPKKTEFLSYHEPLGICLTAASLLGTVISAVVL

GIFIHHRSTPVVRANNSELSFLLLVSLKLCFLCSLLFIGRPRLWTCQLRHAAFGISFVLCVSCIL

VKTMVVLAVFRASKPGGGTTLKWFGAVQQRGTVLGLTSVQAAICTAWLVSYSPEPHKNTQY

YNYKIVYECVVGSPIGFSVLMGYIGLLAVLSFQLAFLARNLPDNFNEAKLITFSMLIFCAVWVA

FVPAYINSPGKYADAVEVFAILASSFGLLVALFGPKCYIILLRPERNTKKAIMDRD

>Tr_16_5_FP

MGTILDLLLLLYFCIHLTFFLSFLFAEAFADLPYCRLRRQFDLNGMHKPGDMILGGLFEVHYTS

VFPDLTFTSEPNQLVCQGFDLAGFRHAMTMAFAIDEINRNISLLPNVTLGYSLYDNCATLVIGF

SAALSMVSGQQEQFLQQEKCLGTPPVLGIVGDSFSTFSIATSDVIGLFKLPIVSYFATCSCLSD

RQRFPSFFRTIPSDAFQVRAVIQILKHFRWSWVGLLVSDDDYGLHVARSFQSDLTRSGGGCL

AYLEILPWGYNPGELTQVVEVMKKSTARVVIVFAHQIHMIQLMEEVVKQNLTGLQWIASEAWT

AAAVLQTTELMPYLGGTLGIAIRRGEIPGLRDFLLGLHPDLHEISNRNSLVRQFWEYTFQCRF

APAPTGWVEGGGLLCTGEEELGRVQTEFLDVSNLRPEYNIYKAVYALAHALDEMLHCVPGR

GPFDEHRCATLESLKPWQLMHYLQQVNFTTAFGDEVSFDENGDALPIYDIMNWLWLPDGRT

XPQRDHCVPKKTEFLSYHEPLGICLTAASLLGTVISAVVLGIFIHHRSTPVVRANNSELSFLLLV

SLKLCFLCSLLFIGRPRLWTCQLRHAAFGISFVLCVSCILVKTMVVLAVFRASKPGGGDILKW

FGAVQQRGTVLGLTSVQAAICTAWLVSYSPEPHKNTQYYNYKIVYECVVGSPIGFSVLMGYIG

LLAVLSFQLAFLARNLPDNFNEAKLITFSMLIFCAVWVAFVPAYINSPGKYADAVEVFAILASSF

GLLVALFGPKCYIILLRPERNTKKAIMDRD

>Tr_16_1_F

MWTASQVSLCSFLCLLSLDSHFYFLECSPLHSCRMQEQFNLSGMHRAGDVVLGGLFEIHFF

SVFPDLSFSSKPQQPTCHGFDVLGFRQAQTMAFAIDEVNRNPHLLPNVTLGYSIYDNCVKLA

IGFRAALSLISGEEERFELTDTCKGSPPVIGIVGDSSSTRSIAISTVLGLYRVPMVSYFATCSCLS

DRQMFPSFFRTIPSDAFQVSAMIQILKHFSWTWAGLLVSDDDYGLHAARSFQSDLAESGGG

CLAYVEVLPWGEDRPELRRIVDVMKASTALVVIVFAHETHMINLMEEVRRQNVTGLQWIASEA

WTAASVLQTPELMPFLAGTLGIAIRRGEISGLREFLQGIHPEERSNNHYGNSLVKQFWEHTF

QCKFAPPPAGQVDTGGRPCTGREHLEDAETEFMDLSNLRPEYNVYKAVYALAYAIDDMLKC

EPGREPFGGKRCGSLKQLEPWQIVYYLEKVNFTTPFGDQVSFDENGDALPIYDIMNWLWLP

DGRAKVQNIGEVKRSPSRDEELTIHEDKIFWNFESKKPPHSVCSESCPPGTRMSRKKGQPV

CCFDCLPCSEGEISNTTDSMECTSCPEDFWSSPQRDHCVPKKTEFLSYHEPLGICLTAASLL

GTVISAVVLGIFIHHRSTPVVRANNSELSYQILVSLKLCFLCSLLFIGRPRLWTCQLRHAAFGIS

FVLCVSCILVKTMVVLAVFRASKPGGGATLKWFGAVQQRMTVIILTSIQAAICLSWILLASPFP

HKNTQYSNERIIYECAVGSTVGFILLLSYIGLLATLSFLIAFYSRKLPDSFNEAKLITFSMLIFCAV
WVAFVPAYISSPGKYADAVEVFAILASSFGLLAALFGPKCYIVLLRPEKNTKNAIMGRV
>Tr_16_2_F
SRVFILCRLILLFSSSSSLYSTSPSCKLWRRFSLNEMHKPGDVVLGGLFEVHYTSVFPELTFTT
EPQQPVCRGFDILGFRHAMTMAFAVEEINRNSNLLPNLTLGYSLFDNCGALVVGFSGALSLA
SSPEMQFLLEEDCEGPPPVVGIVGDHYSTFSIAISSVLGLYKMPIVSYFSTCSCLSDHQRFPS
FFRTIPSDAFQVRAMIQILKHFGWTWVGLLVSDDDYGLHVARSFQSDLVQSGQGCLAYLEVL
PWDGDPSEIRRIVHVIKESTARVLMVFAHEIHMIQLMDEVVGQNVTGRQWVASEALTTAAVLQ
VPHFMPYLRGMLGIAIRRGEIPGLREFLRQVRPDRHSRDTETSMFWEYIFQCKFDPPGLVEA
GEVLCTGQEAIEDADTEFLDLSNLRPEYNVYKAVYALAYALDDALRCEPGRGPFSAHSCAEN
QRLQPWQLVYSLEKVNFTTSFGDQVSFDENGDVLPIYDIMNWLWLPDGRTKVQNVGEVKG
SPFRGEELTLYEDKIFWNFESNKPPQSVCSESCPPGTRMSRKKGQPVCCFDCLPCSEGEIS
NTTDSMECTSCPEDFWSSPQRDHCVPKKTEFLSYHEPLGICLTAASLLGTVISAVVLGIFIHH
RSTPVVRANNSELSFLLLVSLKLCFLCSLLFIGRPRLWTCQLRHAAFGISFVLCVSCILVKTMV
VLAVFRASKPGGGAILKWFGAVQQRGTVLVLTCVQAAICVAWIVSATPEPQKNTQYHNDRIVY
ECAMGSTIGFSALLGYIGLLAILSFLLAFLARNLPDNFNEAKLITFSMLIFSAVWVAFVPAYINSP
GKYADAVEVFAILASSFGLLVALFGPKCYIILFKPNKNTKKAIISRD
>Tr_16_7_FP
TEFLS
HEPLGICLTAASLLGTVISVVVLGIFIHHRSTPVVRANNSELSFLLLVSLKLCFLCSLLFIGRPRL
WTCQLRHAAFGISFVLCVSCILVKTMVVLAVFRASKPGGGATLKWFGAVQQRGTVLGLTSIQ
AAICFAWLLSSSPKPHKNIQYHKDKIVFECVVGSTVGFAVLLSYIGLLAILSFLLAFLARNLPDN
FNEAKLITFSMLIFCAVWVAFVPAYINSPGKYADAVEVFAILTSSFGLLVALFGPKCYIILFRPER
NTKRAIMAR
>Tr_15_1_F
MSQLSRIFTLIVGFGGRELGLGGVLQVVQALTCSQWSTPTEQGLFQDGHVVVGGLFNLHYT
PPDTANNFTQQSHYKACTGLENLPLQYIYAMVFAVEEINHSAALLPGVKLGYHIRDSCALHP
WTTQAALALVAGDSASCELATPADYSAETSEEKGAASVPLIIGGASSNAAKILLGTLSPLSVPL
ISYTASCPCLSDRHRYPTFFRTMASDIYQAQALAQLVLRFNWTWIGAVVANNDYGHVAVKVF
QEQTQGKGVCLAFVETLQRETIVADAVRAARTIQASTARVILVFSWYTDVGHLFRQLQKINVT
DRQFLASEAWSTSEVLLKDPDTSTVASGVVGVAIASQHIPGFDRFLRGLNPSLRPSDKFLQE
FWEEEFGCSPSPPSSETSGDLNASLPPCSGAESLEGVQHPFTDTSHLRVTYNVYLAVYAAA
NALHSLLSCPIHNSPSGTSHCTSPKGIKTTELLQHLSKVNFTTPQGKHLYFRGADIPAMYDLI
NWQSGTDGTLQLVLIGAVAGFDLQLNESEIEWSAKYNQVPVSVCSESCPPGTRKANRKGEP
LCCFDCIPCADGEISNTSGSLQCDRCPPEFWSNDGRTACVPRQLDFLSFNETLGVALTAVAV

SGAVVTTAVFVVFLHYRHTPMVRANNSELSFLLLLSLKLCFLCSLVFIGRPSVWSCRFQQAAF
GISFVLCVSCLQVKTIVVLAAFRSARPGAGALMKWFGPSQQRGSVCIFTCVQARVIICIVWLS
LSPPVPQADLDVPGLQVTLECAMASVVGFSLVLGYIGLLACTCLLLAFLARKLPDNFNEAKLI
TFSMLIFCAVWVAFVPAYISSPGKYSVAVEIFAILASSYGLLFCIFAPKCFIILLRPEKNTKKHLM
MR
>Tr_8_7_FP
DSISCVRCHSEFWSNERRDACIKKKEEFLSYEEMMGALLTVASLLGTCLTAVVMFIFFRYRRT
PIVRANNSELSFLLLFSLTLCFLCSLTFIGRPSGWSCMLRHTAFGITFVLCISCVLGKTMVVLM
AFRATLPGSNVMKWFGPAQQRLSVLGFTLIQVFICLLWLTISPPFPFKNIKDFKDRIILECALGS
AVGFWAVLGYIGLLAMLCFFLAFLARKLPDNFNEAKFITFSMLIFCAVWVTFIPAYVSSPGKFS
VAVEIFAILSSGFGLLICIFIPKCYIILLKPERNTKRNLMGKE
>Tr_4_5_FP
GDNCSQSGMVMAVVGESSSTPSISISCVIGSFDVPLVSHFATCACLSDKQKYPSFFRMIPSD
QFQADALAKLIKHFGWTWIGAVCSDSDYGNNGMAAFLYPSFTPGKRLELLLISFTANTMVIIQ
RKALLLQVSEGGVEELDDGIVHPDARLVGELQQVHEGAYQWAQVGEDESLQRLHQMRRQS
YRPVAVELLGVRCFRHWDDAGRLPQLWHSPQPQVQVEHVTEDPTQLVCAGLQEPGADAVQ
TCRFPGPVLLQGLPHLLKTVNFSQNGYAVSFDANGDPVASYELVNWKKSGSGSIEVVPVGY
YDASLPEGQEFRIFRDITWVDGRKQVPVSVCSDSCPQGTRKVLQKGKPICCYDCVQCPEGE
ISNVTDSPECIPCLDDFWPNPERNACFLKPVEFLSFNEVLGIILAVFSVGGACLAVITAAVFFHH
RASPIVRANNSELSFLLLFSLTLCFLCSLTFIGAPSQLSCMLRHTAFGITFVLCISCVLGKTVVV
LMAFRATLPGSNVMKWFGPPQQRMTVVTFTSIQVLICIVWLVVSPPFPVRNLTTYKERIILECA
LGSSVGFWAVLGYIGLLAAVCLVLAVLARKLPDNFNEAKMITFSMLIFCAVWITFIPAYVSSPG
KFTVAVEIFAILASSFGLILCIFAPKCFIILFKPEKNSKKHLMNKK
>Tr_15_4_FP
SLLLLCVMGARAGLDVAGAMLCSHWGQRSDRNLSADGDVMIGGLFNLYYIPSAVQQEYTQ
LPHYERCSSLDIESLKYMYTMVFAVEEINRDDSLLPGVRLGYGIRDSCFRYPWALDGALSLVT
GDSNSCNVAASSTRSAGGNTGAVEVVPLIIGAASSTTGIMLSSILQSLSVPIISYSASCPCLSD
RAKFPTFFRTIPSDIYQARAMAQLAIRFRWTWLGAVVVNNDYGQLAIQIFQEEIRGKEMCMEF
IETVNRETLTTDARRIALTIQAATARVILIFCWYIDAKEILLELAKRNITGRQFLASEALSTSEELLQ
ELAIAEVANGVLGVAIQSSTIPGFEHFLRSLNPVQRPDDEFLKDFWEMEFKCSPVLQLLHSCL
FVFLFYKGLTMKGVLFPSKGSFFKASLPPCSGAESLVEMDYPFPHTSKLRVAHNVYLAVYAA
AHALHSLLSCPGQDSPPGKSNCSSPNHIRPID
QIFRTSLRWXSGAVVTTAVFVVFLHYRHTPMVRANNSELSFLLLLSLKLCFLCSLVFIGRPSV
WSCRFQQAAFGISFVLCVSCILVKTLVVLAVFRSAQPDCKATMKWFGPSQQRGSVGLFTSIQI
VICGTWLSVSPPKPERDLGFQGSKVTLECTMASVVGFSLVLGYIGLLACTCLLLAFLARKLPD

NFNEAKLITFSMLIFCAVWVAFVPAYVSSPGKYVVAVEIFAILASSYGLLFCIFAPKCFIILLRPEK
NTKKHLMSRN
>Tr_15_3_YP
MSQLSRIFTLIVGFGGRELGLGGVLQVVQALTCSQWSTPTEQGLFQDGHVVVGGLFNLHYT
PPDTANNFTQQSHYKACTGLENLPLQYIYAMVFAVEEINHSAALLPGVKLGYHIRDSCALHP
WTTQAALALVAGDSASCELATPADYSAETSEEKGAASVPLIIGGASSNAAKILLGTLSPLSVPL
ISYTASCPCLSDRHRYPTFFRTMASDIYQAQALAQLVLRFNWTWIGAVVANNDYGHVAVKVF
QEQTQGKGVCLAFVETLQRETIVADAVRAARTIQASTARVILVFSWYTDVGHLFRQLQKINVT
DRQFLASEAWSTSEVLLKDPDTSTVASGVVGVAIASQHIPGFDRFLRGLNPSLRPSDKFLQE
FWEEEFGCSPSPPSSETSGDLNASLPPCSGAESLEGVQHPFTDTSHLRVTYNVYLAVYAAA
NALHSLLSCPIHNSPSGTSHCTSPKGIKTTELLQHLSKVNFTTPQGXHLYFRGADIPAMYDLI
NWQSGTDGTLQLVLIGAVAGFDLQLNESEIEWSAKYNQVPVSVCSESCPPGTRKANRKGEP
LCCFDCIPCADGEISNTSGSLQCDRCPPEFWSNDGRTACVPRQLDFLSFNETLGVALTAVAV
SGAVVTTAVFVVFLHYRHTPMVRANNSELSFLLLLSLKLCFLCSLVFIGRPSVWSCRFQQAAF
GISFVLCVS
>Tr_15_2_F
MSWLLALLVQRPRSPASLLLLCVMGARAGLDVAGAMLCSHWGQRSDRNLSADGDVMIGG
LFNLYYIPSAVQQEYTQLPHYERCSSLDIESLKYMYTMVFAVEEINRDDSLLPGVRLGYGIRD
SCFRYPWALDGALSLVTGDSNSCNVAASAGGNTGAVEVVPLIIGAASSTTGIMLSSILQSLSV
PIISYLASCPCLSDRAKFPTFFRTIPSDIYQARAMAQLAIRFRWTWLGAVVVNNDYGQLAIQIF
QEEIRGKEMCMEFIETVNRETLTTDARRIALTIQAATARVILIFCWYIDAKEILLELAKRNITGRQF
LASEALSTSEELLQELAIAEVANGVLGVAVQSSTIPGFEHFLRSLNPVQRPDDEFLKDFWEME
FKCSPVLQLLHSCLFVFLFYKGLTMKGVLFPSKGSTRKASLPPCSGAESLVEMEHPFTDTSK
LRVAHNVYLAVYAAAHALHSLLSCPGQDSPPGKSNCSSPNHIRPIDVLQHLNRVNFSTPRGE
TFYFQGSDMTARYDLVNWQKTPNGPLKLVLVGRVDGFDLILNESAIQWSTGLNQVPVSVCSE
SCPPGTRKANRKGEPLCCFDCIPCADGEISNTSGSLQCDRCPPEFWSNDGRTACVPRQLD
FLSFNETLGVALTAVAVSGAVVTTAVFVVFLHYRHTPMVRANNSELSFLLLLSLKLCFLCSLVFI
GRPSVWSCRFQQAAFGISFVLCVSCILVKTLVVLAVFRSAQPDCKATMKWFGPSQQRGSVG
LFTSIQIVICGIWLSVSPPKPERDLGFQGSKVTLECAMASVVGFSLVLGYIGLLACTCLLLAFLA
RKLPDNFNEAKLITFSMLIFCAVWVAFVPAYVSSPGKYVVAVEIFAILASSYGLLFCIFAPKCFIIL
LRPEKNTKKHLMSRN
>Tr_4_1_F
MEISVLFLCFISLFDLNSAGDLKAPENSLKQQLGPREDTTGATAPSVKCRLQGSARLPAFSKD
GDFVIGGVFSIHRYTVTVNHNYTTMPEPFRCRVINHRELRLSHAMVFAIEEINNSTELLPGIKL
GYQIHDSCAAVPIAVHVAFQLLNTLDPVFVTGDNCSQSGMVMAVVGESGSTPSISISRVIGSF

DFPLVSHFATCACLSDKQKYPSFFRTIPSDQFQADALAKLIKHFGWTWIGTVCSDSDYGNNG
MAAFLHAAQKEGICVEYSESFYRSHPHSRIKRVADVIRRSTAIIVVAFTSSGDLRILLEELSREP
SPPRQWIGSESWVTDLDLLRFSFCAGTIGFAIQRSVIPGLRDFLLDLSPSKVASSPVLTEFWE
DSFNCRLGKVAVAGERMCDGSEDIMTLQSPYTDTSELRITNMVYKAVYAIAHAIHNAVCQDT
NATTRCSKFTTINPKKVLTQLKTVNFSQNGYAVSFDANGDPVASYELINWKKSGSGSIEVVPV
GYYDASLPEGQEFRIFRDITWVDGRKQVPVSVCSDSCPQGTRKVLQKGKPICCYDCVQCPE
GEISNVTDSPECIPCLDDFWPNPERNACFLKPVEFLSFNEVLGIILAVFSVGGACLAVITAAVFF
HHRASPIVRANNSELSFLLLFSLTLCFLCSLTFIGAPSHLSCMLRHTAFGITFVLCISCILGKTVV
VLMAFRATLPGSNVMKWFGPPQQRMTVVTFTSIQVLICIVWLVVSPPFPVRNLTTYKERIILEC
ALGSSVGFWAVLGYIGLLAAVCLVLAVLARKLPDNFNEAKMITFSMLIFCAVWITFIPAYVSSP
GKFTVAVEIFAILASSFGLILCIFAPKCFIILFKPEKNSKKHLMNKK
>Tr_16_3_YP
VRFDILGFRQAMTMAFAVQEINKNPDLLPNLTLGYHLYDNCGALVVGFSGALALASGQEEAF
ALQGGCAGSPPVLGIVGDSPSTFTIASASVLGLYKIPMVSYFATCSCLTNRQRFPSFFRTIPSD
AFQVCAMIQILKHFGWTWVGLLVSDDDYGLHVARSFQSDLAQSGQGCLAYLEVLPWDGDL
SENRRIVHVIKESTARVLMVFAHESHMINLMEEVVRQKVTGLQWLASEAWTGTTLLQTPDFM
PYLNGTLGIAIRRGEITGLRDFLLRIRPGQSSNNTSYNMVQQFWEYSFQCKFGASGSAEACT
GDENIQQVDAEFLDMSNLRPEYNIYKAVYALAHALDDMLQCEPGRGPFSGGSCADIHKLEP
WQFVHYLQHVNFTTTFGDQVSFDENGDVLPIYDIVNWQWLPNGRTEVQNVGEVKRSPSRG
EELQIHEDKIFWNFESNKPPQSVCSESCPPGTRMSRKKGQPVCCFDCLPCSEGEISNTTDS
MECTSCPEDFWSSPQRDHCVPKKTEFLSYHEPLGICLTAASLLGTVISAVVLGIFIHHRSTPVV
RANNSELSFLLLVSLKLCFLCSLLFIGRPRLWTCQLRHAAFGISFVLCVSCILVKTMVVLAVFR
ASKPGGGAT
>Tr_16_8_FP
XSCTGDENIQQVDAEFLDVSNLRPEYNIYKAVYALAYALDDMLQCEPGRGPFSGGSCADIHK
LEPWQFVHYLQHVNFTTTFGDQVSFDENGDVLPIYDILNWQWLPDGRTQVQNVGEVKRSP
SRGEELQIHEDKIFWNFESNKPPHSVCSESCPPGTRMSRKKGQPVCCFDCLLCSEGEISNT
TDSMECTSCPEDFWSSPQRDHCVPKKTEFLSYHEPLGICLTAASLLGTVISAVVLGIFIHHRST
PVVRANNSELSFLLLVSLKLCFLCSLLFIGRPRLWTCQLRHAAFGISFVLCVSCILVKTMVVLA
VFRASKPGGGAILKWFGAVQQRGTVLGLTSIQAAICFAWLLSSSPKPHKNIQYHKDKIVFECV
VGSTVGFAVLLSYIGLLAILSFLLAFLARNLPDNFNEAKLITFSMLIFCAVWVAFVPAYINSPGKY
ADAVEVFAILTSSFGLLVALFGPKCYIILFRPERNTKRAIMAR
>Tr_8_8_YP
QSLLGLLELLLLASFCQNEELVCRQIGAVERPQLSKDGDILLGGIFSFHSKWKDKWNTYKQK
PPPLQCTSLNFRGFQFAQAMLFAIDEINNSTNLLPGILLGYKIYDACASIARSVRVALALANGN

QVESSLKGPCPKPAQVQAIMGETASSPCMAIATVIGPFHIPLVSKRNGYEYAFGWTWVGAIRT
NDDYGNNGMATFIETAEELGICVEYSVAVFRTDPLDKIRQIIDIIKASTSRVIVAFMANKDMDVL
LSEMSQHNLTGYQWVGSESWISDSHTAAMDVHHILDGAVGLSIPKAHVTGMKEFILDVKQL
NSSSKELFREFWEALFDCKFRNLASTTTENQRECSGHEDLAGVKNSFTYMSLMPIFYNIYKG
VYAVAHALHDILSCNNTCNKTTELDPFMILQRIQRSQFKTKEGDEVYFNENGDPPAKYEIINW
QPTENGFMDFVTVGLYDASLPADRQLTLQTTTFTWAQNSEQVPVSVCSEKCPPGTRKVLQK
GKPVCCYDCLRCADGEISNNTG
>Tr_8_9_YP
QGWALLQLLLLVSFSQAEDRVCLQMGDPENPQLSKDGDIILGGIFSFHSSWKNRRDTYMHK
PLPLQCISLNFRGFQYAQAMLFAIDEINNSSDLLPGITLGCKIYDSCGSIARGVRASLADKNKY
PSFLRTIPSDHYQSRALAQLVKYFGWTWVGAIRTNDDYGNNGMATFIETAEELGICVEYSVAV
FRTDPPEKIQQIIDVIKASTSRVIVGFLSHLDMDVLLIELSQHNLTGYQWVGSEAWIFESHIAAM
DVHHILDGAVGLSIPKAHVTGMKEYILGVKQLNSSSKELFRELWEALFDCKFEDSVSTTTENQ
RECSGHEDLAGVKNTFTDMSLMPILYNIYKGVYAVAHTLHDVLSCNNTCNKTAQLDPFTILQ
HIKRIRFKTKEGDEVYFNENGDPPAKYEIINWQPTENGNVEFVPVGLYDASLPADRQLTLTNR
TLVWAQNSEQVPVSVCSEKCPPGTRKVLQKGKPVCCYDCLRCADGEISNSTG
>Tr_9_3_YP
NSKVPVSVCSESCRPGTRKVPQKGKPLCCYDCISCAEGEVSNSTDANDCKKCPEEFWPNQ
NRDACVPKQVEFLSFTESMGVVLVFFTLLGFFLTLSVTALFVINKNTPLVKANNSELSFLLLFSL
TLCFLCSLTFIGRPSGWSCMLRHAAFGITFVLCISCVLGKTMVVLMAFRATLPGSNVMKWFG
PAQQRLSVLAFTLVQVLICILWLTIGPPFALKNTKHYKEKIILECALGSARHLYLNSKYTAASF
ILQKCVIISLQLSKLHLIMFK
>Tr_9_4_Y
MPQVIAELFFIGLLLKLTSAQVRASSCQILGSPEFPLLSREGDVVIGGAFSVHSKVTQPSLSHQ
EKPAQISCSSVNLREFRFAQSMIFAIEEINKSDFLLPNVSIGYRIYDTCGSTLSSVRAAMALMN
GESTAGKNCSYRSSVHAIIGESESFSTIVLSRLTGPFEIPVISHSATCECLSDRKEHPSFFRTIA
SDLYQSRALAQLVKHFGWTWVGAVNSDSDYGNNGMAIFLTAAQEEGVCVEYTEKFHRAEPE
KLLKVVEVIRRGTARVIVGFLAYVEMNNLLQQLSLHNVTGLQFVGVEAWITANSLVTPTSFGVL
GGSLGFAVEKAAISDLDDFLIGDFWETEFECKETIEDGMAGPATCQENGDLAAFKGYADDVA
ELRYSGNIYKAVYAVAHSLHSTLQCSISGVCDKTVKVTHQQVEIHL
>Tr_8_5_Y
QGWALLQLLLLVSFSQAEDRVCLQMGDPENPQLSKDGDIILGGIFSFHRSWINRRDTYMHK
PLPLQCISLNFRGFQYAQAMLFAIDEINNSSDLLPGITLGCKIYDSCGSIARGVRASLALINSQ
ETIFKLSDKCTKPAQVQAIMGESSSSPNMAVATVIGPFHIPLVGKSQHGFFHYVCVIVMLFPCL
SLKEEITHF*NQRECSGHEDLAGIKNTFTDMSLMPIFYNIYKEVYAVAHALHDILSCNNTCNKT

AQLDPFTILQHIKRIRFKTKEGDEVYFNENGDPPAKYEIINWQPTENGNVEFVPVGLYDASLPA
DRQLTLTNRTLVWAQNSEQVPVSVCSEKCPPGTRKVLQKGKPVCCYDCLRCADGEISNSTG
QVFQ
TVIIIKPTEKRRTPIVRANNSELSFLLLFSLTLCFLCSLTFIGRPSGWSCMLRHTAFGITFVLCISC
VLGKTMVVLMVFRATLPGSKVMKWFGPAQQRLSVLGFTLIQAFICLLWLTISPPFPFKNIKDF
KDRIILECALGSAVGFWAVLGYIGLLAMFCFILAFLARKLPDNFNEAKFITFSMLIFCAVWVTFI
PAYVSSPGKFSVAVEIFAILSSGFGLLICIFIPKCYIILLKPDRNTKRNLMGKE
>Tr_8_6_FP
XLDGAVGLSIPKAHVTGMKEFILDVKPLNSSIEELFREFWEALFDCKFEDSVSTTTENQRECS
GHEDLAVVKNTFTDMSLMPIFYNVYKEVYAVAHALHDILSCNNTCNKTAQLDPFTILHHIKK
RFKTKEGDEVYFNENGDPPAKYEIINWQPTENGNVEFVPVGLYDASLPADRQLTLTNRTLVW
AQNSEQVPVSVCSEKCPPGTRKVLQKGKPVCCYDCLRCADGEISNSTDSISCVRCHSEFW
SNERRDACIKKKEEFLSYEEMMGALLTAASLLGTCLTAVVMFIFFRYRRTPIVRANNSELSFLL
LFSLTLCFLCSLTFIGRPSGWSCMLRHAAFGITFVICISCVLGKTMVVLMAFRATLPGSNVMK
WFGPAQQRLSVLGFTLIQAFICLLWLTISPPFPFKNIKDFKDRIILECALGSAVGFWAVLGYIGL
LAMLCFFLAFLARKLPDNFNEAKFITFSMLIFCAVWVTFIPAYVSSPGKFSVAVEIFAILSSGFG
LLICIFIPKCYIILLKPERNTKRNLMGKE
>Tr_8_4_Y
QSLLGLLELLLLASFCQNEELVCRQIGAVERPQLSKDGDILLGGIFSFHSKWKDKWNTYKQK
PPPLQCTSLNFRGFQFAQAMLFAIDEINNSTNLLPGILGCKVYDSCGSIARGVRASLALINSQ
ETTFKLSDKCTKPAQVQAIMGESSSSLNMAAATVIGPFHIPLISHFATCDCLSDKNKYPSFLRT
IPSDHYQSRALAQLVKYFGWTWVGAIRTNDDYGNNGMATFIETAEELGICVEYSVAVFRTDPP
EKIQQIIDVIKASTSRVIVGFLSHRDIYVLILEMSQHNLTGYQWVGSEGWIFDSHTAAMDVHYIL
DGAVGLSIPKAHVTGMKEFILDVKLLNSSSKELFREFWEALFDCKFEDSASTTTENQRECSG
HEDLAGIKNTFTDMSLMPIFYNIYKEVYAVAHALHDILSC
>Tr_8_1_F
MTPTLMWPEKVWGLFLLVLSFSQACGQVCRHIGEVEKPQLSKEGDVILGGIFYFHNRWKTR
ENAYTAKPLPTECRSLNFRGFQFAQAMLFTINEINKSTDLLPDVIVGYKIYDSCANIVNSIKLVL
ALTNRQDKESASDEESCTKPAQVQAIMGESSSSPCTAIASVIGPFHIPVISHFATCACLSDKNK
YPSFLRTIPSDHYQSRALAQLVKYFGWTWVGAVRSNDDYGNNGMATFIETAEELGICVEYSV
AVFRTDPMIKILQIIDIIKSSTSKVIVTFLSPGDLNVLLQEFSQHNLTGYQWVGSESWISDSHIAA
MDVHHILDGAVGLSIPKAHVTGMKEFIMDVKQLSSSSKELFREFWEALFDCKFEDSVSTTTE
NQRECSGHEDLAGVKNIFTDMSLMPIFYNIYKGVYAVAHALHDILSCNNTCNKTAQLDPFTIL
QHIRSTHFKTKEGDEVYFNENGDPPAKYEIINWQPTENGNVEFVPVGLYDASLPADRQLTLTN
RTLVWAQKKSLQVPVSVCSEKCPPGTRKVLQKGKPVCCYDCLRCADGEISNSTDSISCVRC

HSEFWSNERRDACIKKKEEFLSYEEMMGALLTAASLLGTCLTAVVMFIFFRYRQTPIVRANNS
ELSFLLLFSLTLCFLCSLTFIGRPSGWSCMLRHTAFGITFVLCISCVLGKTMVVLMAFRATLPG
SNVMKWFGPAQQRLCVLGFTLIQVIICILWLSISPPSPNKNFNVAKDRIILECALGSALGFWAV
LGYIGLLAMFCFILAFLARKLPDNFNEAKFITFSMLIFCAVWVTFIPAYVSSPGKFSVAVEIFAILS
SSFGLLICIFIPKCYIILLKPERNTKKNLMGKE
>Tr_7_1_F
MQLAVGVLFVTLMAVRGGDLVCQTYGTKELSQFSMEGDINIGGIFSFHQNPVTIDPALHFNP
EMIQCEGLDPGELQYAFTMMFAINEINNSSDLLPGLILGYRIFDSCPSVPLSIRASLNLMNRYE
SGGDSCSKLSNVHAVIGETTSTSTIGIARTMGPFLIPVISHSATCACLGNRRDYPAFFRTIPSDI
YQSQALAKLVKHFGWTWVGAIRTNSDYGNGGMTAFLKAAEKEGVCVEYSVAIYRTDPRKWF
LEVVDIIKKSTSKVIVAFVDGTDLDILVKELHAQSVTGLQWVGSEGWITYRFIASPENYAVVQG
AVGFAALNTHLPGLQEFLADSRPSTTPGNNGLVELWETVFKCTLSPRAQSPLAACTGKESLR
DASTRFTDVSDASLLNNVYKATYAIAHALHLLLTCKEGEGPFENNTCADRHNVQPWQVLHYL
TLVNFTTKIGDNVHFDEMGDPVPRYALVNWQMDEAGYVLFETIGDYDASRPEGHQFQMND
GVKALWAGENLEVPRSVCSESCLPGTRRAFVKGRPICCFDCIACADGKFSNSTNAVKCDKC
PPEYKSNEERNNCNLKAIEFLTFRELMGILLVAFSVFGACLSTTIALIFFHFRQTPIVRANNSEL
SFLLLFSLTLCFLCSLTFIGRPSEWSCMLRHTAFGITFVLCISCILGKTMVVLMAFRATLPGSN
MMKWFGAAQQRLSVLTFTLVQVLICILWLTINPPFPFKNTKHYKDKIILECALGSALGFWAVLG
YIGLLAVLCFVLAFLARKLPDNFNEAKFITFSMLIFCAVWITFIPAYASSPGKFTVAVEIFAILASS
YGMLFCIFLPKCYVILLKPENNTKKKLMGKV
>Tr_10_1_F
MPVCVCVMLLFALFHGAFGAEDNLKCKMLGRPEFPLLSQEGDITIGGAFTLHSQMSKPSLS
FEETPEDLTCSRINLREFRFAQTMIFAIEEINNSSSLLPNISIGYKVFDTCGLTLPSTRAVMALM
NGKTRTPEGGCSSRTSVHAIIGASESSSTIVMLQISGIFQIPVISHFATCACLSNRKEYPSFFRT
IPSDFYQSRALAKLVKHFGWTWVGAVKSDNDYGNNGLATFIMAAEQEGVCVEYSEGFSWT
DPSEQIARVVTVIKSGSARVLVAFLAQSEMSALLEEAVKQNLTGLQWVGSESWITAGHLALKK
YSAILTGSLGFTIRKTKITGLQEFLLQVNPSQNPQNNLLKEFWETTFGCSFQSDVHGATQCS
GVEKLKDIQNPFTDVSELRISNNVYKAVYAVAHAMHSMLKCGQSGEAVNQSCTTKKDFELK
QVVEHLQSVNFTLQSGERVYFDDYGDPAATYELVNWQRSPEGNTVFVVVGNYDASQPNGR
QFTMNNINITWAARLQKRPLSVCSQSCIPGFRQAVIKGKPICCFTCVACAAGEISNSSNSAEC
LQCPLEFWSNEDHSQCVPKVIEFLSFEETMGALLAAVSLFGAALTSLVFCVFFRFRHTPLVKA
SNSELSFLLLFSLTLCFLCSLTFIGRPSRWSCVLRHTAFGITFALCMSCVLAKTVAVLFAFTAKR
PGNTVFYCSVPLQRTSVFACITLQVIICVLWLTLAPPHPHKNTAHAKERIILECNLGSPVWFWV
VLGYIGLLAVICFILAFLARKLPDNFNEAKFITFSMLIFCAVWVTFIPAYVSSPGKFTVAVEIFAILA
SSFGLLFCIFAPKCYILILKPEKNTKKHMMGRN

>Tr_9_1_FP
NSKVPVSVCSESCRPGTRKVPQKGKPLCCYDCISCAEGEVSNSTDANDCKKCPEEFWPNQ
NRDACVPKQVEFLSFTESMGVVLVFFTLLGFFLTLSVTALFVINKNTPLVKANNSELSFLLLFSL
TLCFLCSLTFIGRPSGWSCMLRHTAFGITFVLCISCVLGKTMVVLMAFRATLPGSNVMKWFG
PAQQRLSVLAFTLVQVLICILWLTIGPPFALKNTKHYKEKIILECALGSAVGFWAVLGYIGVLAVL
CFILAFLARQLPDNFNEAKFITFSMLIFCAVWITFIPAYVSSPGKFTVAVEIFAILASSYGLLFCIFA
PKCFII
>Tr_9_2_FP
MPQVIAELFFIGLLLKLTSAQVRASSCQILGSPEFPLLSREGDVVIGGAFSVHSKVTQPSLSYR
EKPAQISCSSVNLREFRFAQSMIFAIEEINKSDFLLPNVSIGYRIYDTCGSTLSSVRAAMALMN
GESTAGKNCSYRSSVHAIIGESESFSTIVLSRLTGPFEIPVISHSATCECLSDRKEHPSFFRTIA
SDLYQSRALAQLVKHFGWTWVGAVNSDSDYGNNGMAIFLTAAQEEGVCVEYTEKFHRAEPE
KLLKVVEVIRRGTARVIVGFLAYVEMNNLLQQLSLHNVTGLQFVGVEAWITAXFLLKTEDIMWP
GGKREANICHYSNIYILGGLICGKNEIEL
LFNSKVPVSVCSESCRPGTRKVPQKGKPLCCYDCISCAEGEVSNSTDANDCKKCPEEFWP
NQNRDACVPKQVEFLSFTESMGVVLVFFTLLGFFLTLSVTALFVINKNTPLVKANNSELSFLLL
FSLTLCFLCSLTFIGRPSGWSCMLRHTAFGITFVLCISCVLGKTMVVLMAFRATLPGSNVMK
WFGPAQQRLSVLAFTLVQVLICILWLTIGPPFALKNTKHYKEKIILECALGSAVGFWAVLGYIGV
LAVLCFILAFLARQLPDNFNEAKFITFSMLIFCAVWITFIPAYVSSPGKFTVAVEIFAILASSYGLL
FCIFAPKCFII
>Tr_12_1_F
MSWMGWIPSQRGVQLLCLLCCMIIPVVIALLDQSQHCRVIPGSMSLPVLEKRGDIILGGLFSL
HDMVVEPNLSFTSTPPPTQCTRFSFRTFRWMQTMIFAVEEINRNAEILPDITLGYKIYDSCSTP
HQSLKAAIDLMGSEKDSQFEGKLQREGCDGNVPAVIGDGGSTQSLVVARFLGVFHVPQVSY
FSSCACLSDKTQFPAFLRTMPSDLFQVGALVQLVKYFGWTWVGVIAGDDAYGRGGAAIFAN
EVRRLGACIALYEMIPKTQSQAAISSIISNIRSSGARVVLVFAVEQDVARLFDEAVRQKLTGIQW
LASEAWSTAAILSTPKRYHHILQGSMGFAIRRADIPGLQDFLLRLHPSSAEADDDPFLIPFWEE
VFQCSLDPHGHSEAKRPCSGTEELRSVKNIYSDVSQLRISYNVYKAVYALAYAIKAMRSCEK
GSGPFSQQACPDLDNIHPWQLHHYIKQVNYTNRFGDEIKFDENGDPAAMYDLINWQLTPG
GDMDFVTVGKFDDIAGTGRKNLHIEEEKIVWNGNNTQVPLSVCSSICPPGTRKAIRPNYPICC
HDCVVCTAGEISNQTDAIECARCLPEFWSNADRTACVPKQVEFLSFGDTIGIALLVVSLIGSFL
TCAVALVFFYHRTSPIVRANNSDLSFLLLFSLTLCFLCSLTFISPPSQWSCMLRHTAFGITFVLC
ISCILGKTIVVLMAFRATLPGSDVMKWFGPGKQKAIITFSTLVQVVICTVWLVVAPPTPRQYMP
RESAIIILLCDEGSTIAFSLVLGYIGVLACMCFLLAFLARKLPDNFNEARLIAFSMLIFCAVWVAF
VPAYISSPGKYSTLTEIFAILASSYGLLGCIFAPKCYIILMKSEKNTRKHLMSKS

>Tr_8_3_Y

QGWALLQLLLLVSFSQAEDRVCLQMGDPENPQLSKDGDIILGGIFSFHSSWKNRRDTYMHK
PLPLQCISLNFRGFQYAQAMLFAIDEINNSSDLLPGITLGCKIYDSCGSIARGVRASLALINSQ
ETIFKLSDKCTKPAQVQAIMGESSSSPNMAVATVIGPFHIPLISHFATCACLSDKNKYPSFLRTI
PSDHYQSRALAQLVKYFGWTWVGAIRTNDDYGNNGMATFIETAEELGICVEYSVAVFRTDPP
KKIQQIIDIIKASTSRVIVGFLSHLDIDVLILEMSQHNLTGYQWVGSE
WIFESHIAAMDVHHILDGAVGLFIPKAHVTGMKEYILGVKQLNSSSKELFRELWEALFDCKFE
DSVSTTTENQRECSGHEDLAGVKNTFTDMSLMPILYNIYKGVYAVAHTLHDVLSCNNTCNKT
AQLDPFTILQHIKRIRFKTKEGDEVYFNENGDPPAKYEIINWQPTENGNVEFVPVGLYDASLPA
DRQLTLTNRTLVWAQNSEQVPVSVCSEKCPPGTRKVLQKGKPVCCYDCLRCADGEISNSTG
SNWMSKCKLLFGHKHQSHSLILILQQLVLTRLYIHSPDLFEENTKNLTPHIIARPFVQ*HSCSS
VVVENVTRAMLLHFKNNLSQVIKHCSGR
KEGDNTFRTIPSDHYQSRALAQLVKYFGWTWVGAIRTNDDYGNNGMATFIETAEELGICVEY
SVAVFRTDPPDKIKQIIDVIKASTSRVIVGFLSHRDIYVLILEMSQHNLTGYQWVGSEGWIFDSH
TAAMDVHHILDGAVGLSIPKAHVTGMKEFILDVKLLNSSSKELFREFWEALFDCKFEDSASTT
TENQRECSGHEDLAGIKNTFTDMSLMPIFYNIYKEVYAVAHALHDILSCNNTCNKTAQLDPFT
ILHHIKRIRFKTKDGDEVYFNENGDPPAKYEIINWQPTENG
