# Supplementary material for: Evolution and origin of vomeronasal-type odorant receptor gene repertoire in fishes
Source: BMC Evol Biol. 2006 Oct 3;6:76. doi: 10.1186/1471-2148-6-76 (PMC1601972; doi:10.1186/1471-2148-6-76)
Supplement: Additional File 10 — Deduced amino acid sequences of pufferfish V2Rs. [file 1471-2148-6-76-S10.pdf]

>Tn_12_1_F
TFLSLLWCVINATTTSHHCRVIPGSLSLPVLEKRGDIILGGLFSLHDTVVEPNMTFTSTPAPTQ
CTRFSFRTFRWMQTMIFAVEEINRNPELLSNITLGYKIYDSCSTPHQSLKAAIDLMGSEKDSQ
SEGEMQSEGCDGNVPAVIGDGGSTQSLVVARFLGVFHVPQVSYFSSCACLSDKK
FPAFLRTMPSDLFQVGALVQLVKHFGWTWVGVVAGDDAYGRGGAAIFANELQKLGACVAFY
EMIPKIQSQAAVSAIISNIRSSGARAVLVFAVEQDAARLFEEADREGLTGIQWLASEAWSTAAVL
STPKKYHHILQGSMGFAIRRAHIPGLQDFLLRLHPSSPDADEDPFLVPFWEEVFQCSLGTRS
KDRHTRSEGKPPCSGTENLQSVKNIYCDVSQLRISYNVYKAVYAIAHAIKAMRSCVRGNGPF
SQQACPDLDNIQPWQLHHYIKQVNYINRFGDEIKFDVNGDPAAMYDLVNWQLTPSGDMEFV
TVGKFDKIGADGRKNLHIEEEKIVWNGNKSQVPLSVCSSICPPGSRKAIRPHFPICCHDCVVC
AAGEISNQTDAIECVRCLPEFWSNADRTACIPKQVEFLSFSDIIGIVLLVISLIGSFLTCTVALVFF
YHRTTPIVKANNSDLSFLLLFSLTLCFLCSLTFISQPSRWSCILRHTAFGITFVLCISCILGKTIVV
LMAFRSTHSSKVLKWFGPVRQKAIITFSTLVQVVICTVWLVLAPPTPQRYMPREGAIIILLCDE
GSTLAFSLVLGYIGLLASLCFILAFLARKLPDNFNEARLIAFSMLIFCAVWVAFVPAYISSPGKY
STLTEIFAILASSYGLLGCIFAPKCYIILIKSEKNTRKHLMSKR
>Tn_9_1_F
MPQVMTALLFVGPLLTWAVPVRAFSCKILGSPERPLLSRRGDVVIGGAFSIHSKITQPPLSYQ
TKPAQLLCFSVNLREFRFAQTMIFAIDEINRSEFLLPNVSIGYRIYDNCGSTLSSVRAAMALMN
DDEKTAGNNCSRLSSVHAIIGESESSSTIVLSRLAGPFKIPVISHSATCECLSDRKAHPSFFRTI
ASDRYQSRALAQLVKLFGWTWVGAVNSDSDYGNNGMAIFLVCVGYTEKFHRAEPEKLLKVV
EVIR
STARVIVGFLAHVEMNDLLQQLSLHNVTGLQFIGVEAWITANSLVTPTSFNVLGGPLGFAVEK
ANIDGLDDFLIRDFWETKSECKEMKEDDATGTTTCGENHDLTEFKGYADDVAELRYSSNIYK
AIYAVAHSLHSFLKCSESNVCNKTMNVTPQQVGITHWDSLKAVNFTTKTGEQVWFDGTGAT
VARYEVVNWQRSSDGSIQFKPVGFYDASLPPGLRFLLRVEDIMWPGGKTEVPVSLCSESCR
PGTRKVPQTGKPLCCHDCISCAEGEISNSTDANDCEKCPEELWPNQNRDACVPKTVEFLYF
TEFMGIVLVFFTLLGVFFTLTVTALFLIHKDTPLVRANNSELSFLLLFSLTLCFLCSLTFIGRPSE
WSCMLRHTAFGITFVLCISCVLGKTMVVLMAFRATLPGSHVMKWFGPAQQRLSVLTFTLVQV
LICILWLTNDPPFPLRNTKHYKGKIILECALGSAIGFWAVLGYIGVLALLCFILAFLARQLPDNF
NEAKFITFSMLIFCAVWITFIPAYVSSPGKFTVAVEIFAILASSYGLLFCIFAPKCFIIVLKPQLNTK
KHMMGKT
>Tn_10_1_F
MSDSTCVIFLFVLFHEAFGAEDSLRCEMMGRPEFPLLSKKGDIIGGAFTLHNQMSKPSLSFE
VTPEDLTCSRINLREFRFAQTMIFAIEEINNSSLLLPNISIGYKIFDTCGLTLPSTRAVMGLMNG
KTNASDRGCSRRASVHAIIGSSESSSTIVMLQISGIFHIPMISHFATCACLSNRKQYPSFFRTIP

SDFYQSRALAKLVKHFGWTWVGTVKSDNDYGNNGLATFIMAAKQEGVCVEYSEGFSWTDP
SEQIARVVTVIKSGSAKVLVAFLAQSEMSVLLEEAVKQNLTGLQWVGSESWITAGHLAVKKYS
AILMGSLGFTIRKTKITGLQKFLLQVNPSQDPQNNLLKEFWETTFSCSFQSDMHQGTQCSG
TEKLNDIQNPFTDVSELRISNNVYKAVYAVAHAMHAMFKCDQSGEAVNQSCTSNEDFELKQ
VVEHLQRVNFTLQSGETVYFDDFGDPAATYELVNWQRSQAGNTVFMVVGNYDASQPNKRQ
FTMNSMNITWAAGLQKRPQSVCSQSCIPGFRQAVIKGKPICCFTCIACADGEISNSSNSAEC
LPCPLELWSNEHHSQCVPKMIEFLSFEETMGALFAAVSLFGAALTSLVFCIFFRFRHTPLVKAS
NSELSFLLLFSLYLCFLCSLTFIGRPTRWSCMLRHTAFGITFALCMSCILAKNVAVLFAFTSKRP
GKTFFYCSVPLQRTSVFSCIILQVLICVLWLTFAPPFPYKNTTHAKEHIILECHLGSPVWFWVV
MGYIGLLAVICFILAFLARKLPDNFNEAKFITFSMLIFCAVWITFIPAYVSSPGKFTVAVEIFAILAS
SFGLLFCIFAPKCYILVFKPEKNTKKHMMGRN

>Tn_7_1_PF
TTLLAVIEMF
SLENNVSVDTCKICTVCLFVRQNCLAYSCSKLSNVHAVIGETTSTSTIGVARTMGPFLIPVISH
SATCACLGNRRAYPSFFRTIPSDIYQSQALAKLVKHFGWTWVGAIRTNSDYGNGGMTAFLEA
AQKEGVCVEYSVAIYRTDPRKWFLEVVDTIKKSTSKVIVAFVDGTDLDILIKELYAQSVTGLQW
VGSEGWITYRFIASPVNYAVVKGAVGFSALNAHLPGLQEFLTESRPSTTPGNDGLVELWETVF
KCTLNLGVQSSLAACTGKESLRNASTRFTDVSDGSLLNNVYKATYAIAHALHMIFTCKDGEG
LFENNTCADRHNVQPWQVLHYLKQVNFTTKIGDHVFFDEMGDPVPRYALVNWQMDDAGYV
MFKTIGDYDASRPEGRQFQMKDGVKALWAGGNLEVPKSVCSKNCLPGTRRAFVRGKPICC
FDCITCADGEFSNSTNAVKCDKCLPEYKPNEERNKCHLKAIEFLTFRELMGTLLATFSVFGAC
LSMAMALIFFYFRQTPLVKANNSELSFLLLFSLTLCFLCSLTFIGRPSDWSCMLRHTAFGITFV
LCISCVLGKTMVVLMAFRATLPGSHVMKWFGPAQQRLSVLTLTLGQVLICILWLTIDPPFPFK
NTKHYKEKIILECAMGSAVGYWAVLGYIGLLAVLCFILAFLARKLPDNFNEAKFITFSMLIFCAV
WITFIPAYASSHGKFTVAVEIFAMLASSYGMLFCIYLPKGYIILLKPEYNTRKNLIGKV

>Tn_8_1_F
QGWALLQVLLVVSFSQAEDSVCLQWGGPQNPQLSKDGDIILGGIFSFHSSWKNTRDTYVNK
PLPLQCISLNFRGFQFAQAMLFAIDEINNSSDLLPALTLGYKIYDACGSIARGVKVALALANSE
ERTFNLLEKCSKPAKVQAILGETSSSPCMAIATVIGPFYIPLISHFATCACLSDKNKYPSFLRTIP
SDYYQSRALAQLVKYFGWTWVGTVRSNDDYGNNGMATFVDTAQELGICVEYSVSVFRTDP
PDKIEQIIDIIKASTSRVIVAFLSHMDIDVLIHEMSHHNLTGYQWVGSEGWIFDHHTAATDLHHI
LDGAIGLSIPKADVAGMKKFILDVKQLNSSSQELFREFWETLFNCKFKDLAPPSENQRECSG
HEDLTGVKNSFTDMSLMPIFYNVYKGVYAVAHALHDILSCNNTCNKTAQLDPFTILQRIKKIHF
KTKEGDEVYFNENGDPPAKYEIINWQPTQNGIVDFVTVGLYDASLPAKKQLKLQNKTLVWAQ
NSEQVPVSACSKKCPPGTRKLLQKGKPVCCYDCLRCADGEISNSTDSITCERCHSEFWSNE

RRDRCIKKKEEFLSYEEMMGALLAASSILGTCVTAVVMFIFFKFRQTPIVRANNSALSFLLLFS
LMLCFLCSLTFIGRPSDWSCMLRHTAFGITFVLCISCVLGKTMAVLMAFRATLPGSHVMKWF
GPAQQRVSVLGFTLIQVFICILWLTISPPFPFKNIKDFKDRIILECALGSAVGFWVVLGYIGLLAM
FCFILAFLARKLPDNFNEAKFITFSMLIFCAVWVTFIPAYVSSPGKFSVAVEIFAILSSGFGLLICI
FLPKCYIILLKPERNSKKNLMGRA
>Tn_8_2_F
ERRCALLQVLLVVTFSQAEDSVCLQWGGPQNPQLSKDGDIILGGIFSFHSSWKDTRDTYVN
KPLPLQCISLNFRGFQYAQAMLFAIDEINNSSDLLPAITLGYKIYDACGSIARGVKVALALANS
EERTFNLLEKCSKPAKVQAILGETSSSPCMAIATVIGPFYVPLISHFATCACLSDKNKYPSFLR
TIPSDYYQSRALAQLVKYFGWTSVGTVKSNDDYSNYGMATFVDTAQELGICVEYSVSVFRTD
PPDKIEQIIDIIKAAASRVIVAFLPNMDIEVLIHEMSHHNLTGYQWVGSEGWIFEKHTAAKDLYHI
LDGAIGLSIPKADVAGMKKFILDVKQLNSSSQELFREFWETLFNCKFKDLAPPSENQRECSG
HEDLTGVINGFTDMSLMPIFYNVYKGVYAVAHALHDILSCNNTCNKTAQLDPFTILQRIKKIHF
KTKEGDEVYFNENGDPPAKYEIINWQPTQNGIVDFVTVGLYDASLPAKKQLKLQNKTLVWAQ
NSEQVPVSACSKKCPPGTRKLLQKGKPVCCYDCLRCADGEISNSTDSITCERCHSEFWSNE
RRDRCIKKKEEFLSYEEMMGALLAASSILGTCVTAVVMFIFFKFRQTPIVRANNSALSFLLLFS
LMLCFLCSLTFIGRPSDWSCMLRHTAFGITFVLCISCVLGKTMAVLMAFRATLPGSHVMKWF
GPAQQRVSVLGFTLIQVFICILWLTISPPFPFKNIKDFKDRIILECALGSAVGFWVVLGYIGLLAM
FCFILAFLARKLPDNFNEAKFITFSMLIFCAVWVTFIPAYVSSPGKFSVAVEIFAILSSGFGLLICI
FLPKCYIILLKPERNSKKNVMGRA
>Tn_6_1_F
VCRLRDFAQMPELAQDGDFVIGGIFSFRTGQDYVSDTFQHIPEAPKCKNFNYREFKFAQTVIF
AVGEINRNPDLLPNLKLGYKIYNACGTMGILRAALALVSGLENEINDENCTKTETIQAILGHSG
SRPTIAFAQVVGRFHIPVISHFATCACLSNRKEYPTFFRTIPSDYYQSTALAKLVKHFGWTWIG
AIAVDNEYGQGGIATFIQAAQEYGVCTEYSESFSSEGRPDTLQRIVNVVKRATSKVIVAFMSH
REIGLLAQELHKQNITGLQWVGSDAWITDHSLTDSEGHSILVGSLGFTVSKAKILGLEEHLRQ
LHPAQFPVSQFMVDFWEDRFDCSLNATGNKQRRPCSGFESLQNDTSLFTDETELRFTNNVY
KSVYA
AHALDNLMKCEKGKGP
SNGGCADPKHIQPWQILHYINTVRFNTSEGETIYFDNNGDSPARYELVSLQMTNKGTLEGAT
VGIYDASLPENLQFIISDGAIIWGNGKMEVPVSVCSNSCLPGTHKVLQKGKPICCFDCVPCPA
GEISNITNSIHCIKCPTLFWSSKERDACVPKTIEFLAHEELLGTLLVLCSLLGVFLTTIMFLVFYC
HKETPLVRANNSELSFLLLFSLTLCFLCSLTFIGRPSEWSCMLRHTAFGITFVLCISCVLGKTVV
VLLAFRATLPGSNVMKWFGPAQQRLSVLAFTFVQVLTCTLWLTISPPYPFQNMEYYNDRIILE
CALGSAVGFWAVLGYIGVLAMFCFILAFLARNLPDNFNEAKFITFSMLIFCAVWITFIPAYVSSP

GKFTVAVEIFAILASSFGLLFCIFLPKCYIILFRPELNTKKHIMGKA
>Tn_6_2_F
IVLFTYCLLLIGAMSENQTCTVRGQTGFMEFSKEGDLIIGGVFSLTSTRVLVDNDYQAIPYTYC
NRWNDRELKFARTVIFTVEEINRDAELLPGVMLGYRLYNGCGSENLIRAALEAVTTESCSGQV
QALLGHSSSGVTEDINVILSPLSIPQVSHLSTCACLSDKKQYPTFFRTVPSDHFQISGLVQLLK
IFNWRWVGIVYSVGSYSDEGTAHFVKEAKKDGICVEYRLPFSQVSRKKTRAIVQALQESSSR
VVLLFLSLSNTKYFLQEMESYNISDKQWLGSESWITQVDLASAKRRHILQGTLGFALPQAPIP
GLGDFLLSLKPSDEPQSDLIKAMWEIFFKCSFSLSNTSAACTGTEDLRMVSNDYTDVRHFRA
ENNVYKAVYLVAHALHALLQCKNGSNPTTGKPCVNKTEVKPKLLLEHIKYVNFTTKYGSKVF
FDENGDSVAQYDLVNWQMKEDGSVNIVTIGQYDTS
GEELKLTDSTKIVWGGNHKEVPRSVCREPCPPGTRKAINKLKPVCCFDCFECPQGTISNQTD
SLDCFSCLPELWPNDQKNQCLPKPSEYLSYREITGALLCGFGCLGVFLSVLTTIIFLIHKETPIV
KANNSELSFLLLLSLKLCFLCSLTFIGRPSEWSCMLRHTAFGITFVLCISCVLGKTVVVLMAFR
ATLPGSKVMKWFGPAQQRLSVLIFTLIQVVICIFWLTTNPPFPLRNMSYYKEKIILECALGSAV
GFWAVLSYIGILAILCFILAFLARKLPDTFNEAKLITFSMLIFCAVWITFIPAYISTPGKFTVAVEIFA
ILASSFGLLFCIFFPKCYIIIFKPERNAKKVCWERY
>Tn_2_1_F
DLISLYSFCFVTVFSDLMTNATFPPSSLCRLQNTFQPGFAASGDFIIGGMFPLHFNQETPDLN
NTYRPAPVKCNGFDPRAYRWAQTMKLAVEEINQSKDLLPNHILGYKLFDSCGYPLTGQRAA
LSLLNDPSTDGSPTCTGTPPLLAVIGESSSSLSVMLSGILQPFRIPVISYFSSCACLSDKRKYP
NFFRVIPNDDYQVKAIAQ
LVHFNWTWVGLLLCDREYGQFAAEGLLRELRGTKVCGAYQKIIPLVYDRQRIQEILQVMRSS
SAKVVVVFSAEVEMIPLMRDYIKQNITGIQWIASEAWVTASVFTGSKYYPYLGGTIGLGIRKGH
IPRLSDYLLTVNPQTYPNDLLFEELWEALYGCRPSISSSSSVPLCTGQEPLLEQHSAFMNTSS
PRVAYNVYKAVYAIAHSLHNLLVCQPKPGPFKNDTCVQNNNVQPWQLQYYLQEVRFKIAGE
EVDFDQKGDSIPYYDIISWQMGVDGKIKFVNVGLFDGSKPPGKELVIQEDRIVWAGHQREAS
LSICSASCLPGTRKAIRKEVPVCCFDCIPCDTGKISNETDSVDCMFCPEDFWSNPNKTACIPK
KVEYLAYDSLGIALMVTAVVGACTTIAIFAVFFYHRNTPIVRVNNAELSFFILLALVLCFLCSLVFI
GEPTSWSCMLRHTAFSITFSLCFSCILGKTLVVLAAFTATRPGYNIMKWLGPKQQRAIIFSCTL
VQVIICIAWLIEAPPAPFRNTEYEHSKVILECSVGSSVAFWCVLGYIGLQACLSFILAFLARKLP
GNFNEAKFITFSMLIFCAVWLAFIPAYVSSPGNYSDAVESFAILASSFGLLFCMFAPKCYIILLK
PERNTKQNLMAKE
>Tn_14_1_Y
TLLFWAIASIPFPVFSGLQQIANEKNNIILDSGLEADGGNDAFASLPQCVKATDAEHPALHAG
GDVVIGGIFPLHYSASVPPQTYTNKPELLTCSGFDHRAFRWMMTMVFAVMEINNNSSLLPG

VKLGYRIVDGCDHIPTSLQALLSLVKESMAGEQVMKTIPACLTDSPVAAVIGLASSSPTRAAA
HILGSFNIPLVSYFATCTCLNDKHEYPSFLRTVPSDLFQIGGLVQMVTFMGWFWVGTIGTTDD
YSLYGIQAFSHQLRQQSGCVEFQLIIPKSPTAAEIKELADKLQSSTARVVVVFAREGQLLDFFL
ELIYRNVTGIQWIASEAWVTASLLTTPRFHALLGGTLGFSFPGVEIPGLKEFLINVRPSPEPGM
EFVNMFWEDIFGCVLKFTGEDLRGDNVNALKESMKLNDLGRSLDTKPICTGSEDLRYTHSS
YIDVSQVRISYSVYKAVYAIAHALHSLLKCESPVSDMDLTCKKHEPFTSKQLLQHLKSVNFTN
QFKEKVYFDEKGEPVPLYDVINWQKDSRSNIRFVKVGSYDGSAPLKQRLQIDPNAIVWTGG
QSKVPVSQCSAPCPPGNRQARRPGQPQCCFDCVPCADGQISNQTGSTECQSCPEYYWP
DKDKVKCLPGIEEFLSFSEAMGIVLVILSLLGVILAFTLTIIFHHFRSTAIVKANNSEISFLLLLSLK
LCFLCSLLFIGQPSLWKCRLRQAAFGISFVLCLSCLLVKTIVVLFAFR
ANLKARCLAPKLFGPSRQRTLILLTTAPQVCLCTAWLLAAPSFPFKNPTYQALTGKIVVECKEP
WPPGFYLVLG
I
LLAFIYLLLAFVRRKLPDTFNEAKLITFNMMIKWDVWISLKPAYVRSTGKLTVAVEVFAILASSF
GLLLCIFLPKCFILLLHPERNVKRGMTGKY
>Tn_4_2_PY
SALFLGLISLCDLSSAANLKASGNSLEEQVGLREDRTGAGAPFEKCQLQGSARLPAFSMAG
DFVIGGVFSIHR
KVTEEHNYTTVPEPFRCSGRIHPAQLQFSRAMVFAIEEINNSTELLPGIRLGYQIYDSCAAVPIA
VHAAFQLLNGLDPVFDTGDNCSQSGMVMAIVGESGSTHSISISRVIGSFDIPLVSHFSTCACL
SDKQMYPTFFRTIPSDQFQADALAKLVKHFGWTWIGAVCSDSDYGNNGIAAFLRAAQKEGI
CVEYSECL
>Tn_4_1_F
STLFLGLISLCDLNSAANLKAAGNSLEQQVGLREARTGAGAPFMKCQLQGSARLPAFSMAG
DFVMGGVFSLHHYKVTEEHNYTTVPEPFRCSGSIDPRELRLSHAMVFAIEEINNSTELLPGIR
LGYQIHDSCAAVPIAVHVAFQLLNGLDPVFDTGDNCSQSGMVMAVVGESGSTPSISISRIIGS
FDIPLVSHFATCACLSDKQMYPTFFRTIPSDQFQADALAKLVKHFGWTWIGAVCSDSDYGNN
GIAAFLHAAQKEGICVEYSESFYRTHPYSRIKRVADVIRRSTANVVVAFTSSGDLRILLEELSRE
PPPPRQWIGSESWVTDLDMLRFSFCAGTIGFAIQRSVIPGLREFLLGLSPSKAASSSLLTEFW
EDSFNCRLDKAAAAGERVCDGSEDITTLQSPYTDTSE
RITNMVYKAVYAIAHAIHNAVCQGSNSTNQCDKFTRINPKQVLTELKRVNPFHNGETVSFDAN
GDPVASYELVNWKKRDSGSIDVVPVGYYDASQPEGQELRIYRDITWVDGRTQVPQSVCSNS
CPPGTRKVLQKGKPICCYDCVQCPEGEISNVTDSPDCVPCINNFWPNPVSNACFPKPVEFL
SFSEVLGMILAVFSVGGACLAVIIAAVFFHHRACPIVRANNSELSFLLLFSLTLCFLCSLTFIGAP
SQWSCMLRHTAFGVTFVLCISCVLGKTVVVLMAFRATLPGSNVMKWFGPPQQRLTVFTFTAI

QVLICIVWLVVSPPYPVKNLSTYKDRIILECALGSALGFWAVLGYIGLLAAVCLVLAVLARKLPD
NFNEAKLITFSMLIFCAVWITFIP
YVSSPGKFTVAVEIFAILASTFGLILCIFAPKCFIILFKPEKNTKKKLMNKK
>Tn_16_1_Y
AQPFFFFFFFFHCLMFEKQPTYV
SCFGEKSFIEMIFKIFFQKLGLGNHFS
MCHCITFMLRELKTIPSTLYQTFLFIRYFFVFFSGLNKIMKHLGAKIHSMSPKVEARMAKISTAT
VNFPG
SYFATCSCLSNRQMFPSFFRTIPSDAFQVRAMIQILKHFGWTWVGLLVSNDDYGHHAARSF
QSDLAESGGGCLAYLEVLPWGEDWPELRRIVDVMKKSTARVVIVFAHESHMITLMEEVMRQ
KVTGLQWIASEAWTAASVLHTPRLVPYLAGALGIAIRRGEISGFREFLLQIHPGEHFNDSVETN
MVKQFWEHTFQCRFAPPPAGQADGRERLCTGRELLKDAETEFMDLSNLRPEYNVYKAVYA
LAYALDNMLKCKQGQGPFSGQRCGRLNQLEQWQLVYYLEKVNFTTPFGDQVSFDENGDVL
PIYDIMNWLWLPDGKIKVQNVGEVKKSPLRREEVRIQENQIFWNFEFKQPPQSVCSQSCPP
GTRVSRRKGQPVCCFDCLTCSEGKFSNKTNSMECTSCPEDFWSCSQRDHCVPKKTEFLSY
HDPLGICLTVASLLGTVISAVVLGIFIHHRSTPIVRANNSELSFLLLVSIKLCFFCSLLFIGHPRLW
TCQLRHAAFGISFVLCVSCILVKTMVVLAVFSASKPGGVAILKWFGAVQQRGTVLGLTLVQAAI
CIVWLLSSPPKPHKNTQYHTDKIIFECVIGSTVGFAVLLSYISLLAILSFLLAFLARNLPDNFNEA
KLITFSMIIFSAVWVAFVPAYINSPGKYADAVEVFAILTSSFGLLVALFGPKCYIILFQPERNTKRA
IMAR
>Tn_4_3_P
VWLVVSPPYPVKNLSTYKDRIILECALGSALGFWAVLGYIGLLAAVCLVLAVLARKLPDNFNEA
KLITFSMLIFCAVWITFIPAYVSSPGKFTVAVEIFAILASTFGLILCIFAPKCFIILFKPEKNTKKYLM
NKK
>Tn_16_2_PY
SHLLPNVTLGYSLYDNCIKLAIGFRAALSLISGEEEQFILTDDCKGSPPVIGIVGDSSSTPSIAIS
TVLGLYRVPMVSYFATCSCLSNRQMFPSFFRTIPSDAFQVRAMIQILKHFGWTWVGLLVSND
DYG
HAARSFQSDLAESGGGCLAYLEVLPWGRRLARV
RIVDVMKKSTARVVIVFAHESHMITLMEEVMRQKVTGLQWIASEAWSAAFVLHTPRLVPYLAG
TLGIAIRRGEISGFREFLLQIHPGEHFNDSIETNMVKQFWEHTFQCRFAPPPAGQADGRERLC
TGRELLKD
>Tn_7_CaSR_F
MRLALCYLVLLGSGYVISTYGPNQRAQMTGDILLGGLFPIHFGISSKDENLAARPESTKCVRF
NFRGFRWLQAMVFAIEEINNSSTLLPNITLGYRIFDTCNTVSKALEATLSFVAQNKIDSLNLDE

FCNCTDHIPATIAVVGAAGSAVSTAVANLLSLFYIPQISYASSSRLLSNRNQYKSFMRTIPTDEY
QATAMADIIEYFEWNWVIAVASDDDYGRPGIEKFEKEMEERDICIHLNELISQYFGDHEIKALV
DRIENSTAKVIVVFASGPDTEPLIKEMVRRNITDRIWIASEAWASSSLIAKPEYLDVVAGTIGFVL
KAGNIPGFREFLQQVQPKRDGHNEFIREFWEETFNCYLEDSPRLHESENGSDSFRPLCTGE
EDITSVETPYLDHTHLRISYNVYVAVYSIAQALQDILSCTPGHGLFANNSCADIKKMEAWQVL
KQLRHLNYTNNMGEKVHFGENADMEANYTIINWHRSAEDGSVVFREVGYYHMHARRGAKL
LIDNTKIMWNGYSSEVPFSNCSD
CEPGTRKGIIDSMPTCCFECTECSDGEYSNHKDASICTKCPNNSWSNGNHTFCFLKEIEFLA
WSEPFGIALAICAVLGVLLTAFVMGVFVKFHNTPVVKASNRELSYVLLFSLICCFSSSLIFIGEP
QDWSCRLRQPAFGISFVLCISCILVKTNRVLLVFEAKIPTSVHRKWWGLNLQFLLVFLCTFVQV
MICVVWLYNAPPSSYRNHDIDEIIFITCHEGSVMALGFLIGYTCLLAAICFFFAFKSRKLPENFT
EAKFITFSMLIFFIVWISFIPAYFSTYGKFVSAVEVIAILASSYGMLACIFFNKVYIILFKPCRNTIEE
V
>Tn_4_4_YP
SALFLGLISLCDLSSAANLKASGNSLEEQVGLREDRTGAGAPFEKCQLQGSARLPAFSMAG
DFVIGGVFSLHDFRVTEKPNYTTVPEPFRCSGRIHPAELQFSRAMVFAIEEINNSTELLPGIRL
GYQIHDSCAAVPIAVHAAFQLLNGLDPVFDTGDNCSQSGMVMAVVGESGSTQSISISRIIGSF
DIPLVSHFATCACLSDKQMYPTFFRTIPSDQFQADALAKLVKHFGWTWIGAVCSDSDYGNNG
IAAFLHAAQKEGICVEYSETFYRTHPYSRIKRVADVIRRSTAVVIVAFAAPTEMRILLEELSREPP
PPRQWIGSESSVTDPDMLRFSFCAGTIGFAI
>Tn_16_3_Y
HETLFLFVTLVTLFLSAFTSIKAPSCKLWRTFNLNEMHKPGDVLLGG
FQVHYSSVFPEWAFN
EPQQPTCSGFDILGFRHVM
MAFAIEEINKNSDLLPNVTLGYRLYDNCGTLIVGFSGALSLASGQEEEFPLQDDCAGSPPVLG
IVGDSPSTFTIASASVLGLYQLPMVSYFATCSCLTNRQRFPSFFRTIPSDAFQVQAMIQILKHF
RWTWVGLLVSDDDYGLHVGRSFQSDLVQSGEGCLAYLEVLPWDGDPVEIRRIVNVIKKSTAR
VLMVFAHKFHVISLMNEVVRQNVTGLQWLASEAWTGAAEIQTPQFMPYLRGTLGIAIRRGEIT
GLRDFLLQIRPEQISNITRNNMVQQFWEYSFQCKFGGSNSSEACRGDEDIERVDPEFLDVSN
LRPEYNIYKAVYALAYALDDLLRCEPGRGPFSEHSCADIHRLKPWQLVHYLQHVNFSTTFGD
QVSFDQNGDVLPIYDIVNWLWFPDGRVKVQNVGEVKRTLLRGDEVTLHGDKIFWSFEYNKP
PQSVCSQSCPPRTRVSRRKGQPVCCFDCLTCSEGKFSNKTNSMECTSCPEDFWSSSQRD
HCVPKKTEFLSYHDPLGICLTVASLLGTVISAVVLGIFIHHRSTPVVRANNSELSFLLLVSIKLCF
LCSLLFIGRPRLWTCQLRHAAFGISFVLCVSCILVKTMVVLAVFSASKPGGVAILKWFGAVQQ
RMTATILTFIQVAICILWILLASPFPRKNTQYSNEKIVYECAIGSTVGFSLLLSYIGFLAILSCLIAF

YSRNLPDSFNEAKLIAFSMLIFSAVWVVFVPAYISSPGKYADAVEVFAILASSFGLLVTLFGPKC
YIILLRPERNTKKAIMGRV
>Tn_16_4_PY
HETMFLFVTLVILFLSAFTSIKAPSCKLWRTFNLNEMHKPGDVLLGGLFKVHYRSIFPEWAFT
SEPQQPTCSGFDILGFRHVMTMAFAIEEINKNSNLLPNVTLGYRLYDNCGTLIVGFSGALSLA
SGQEEEFPLQDDCAGSPPVLGIVGDSYSTFTIASASVLGLYQLPMVSYFATCSCLTNRQRFP
SFFRTIPSDAFQVQAMIQILKHFRWTWVGLLVSDDDYGLHVGRSFQSDLVQ
GKGCLAYLEVLSWGRDPVEIRRTVNVIKKSTARVLMVFAHEFHMIPLMNEVVRQNVTGLQWL
ASEAWTGSAALQTPQFMPYLRGTLGIAIRRGEITGLRDFLLQIRPEQISNITRYNIVQQFW
YSFQCKFGGSNSTEACRGDEDIERVDPEFLDVSNLRPEYNIYKAVYALAYALDDLLRCEPGR
GPFSEHSCADIHRLKPWQLVHYLQHVNFSTTFGDQVSFDQNGDVLPIYDIVNWLWFPDGRV
KVQNVGEVKRTLLRGDEVTLHGDKIFWSFEYNKPPQSVCSQSCPPGTRVSRRKGQPVCCF
DCLTCSEGKFSNKT
>Tn_15_2_Y
SRIFTLLLGFGGRELGRGGLLQLVQAQTCTQWSTPSEPGLFQDGDVVVGGLFNLHYKPPDT
THSFTQQPNYKPCTGVSFLISGLENLPLQYIYAMVFAVEEINHSSTLLPGVKLGFHIRDSCALH
PWTTQAALCLVGGDNASCYFSTPSGYSPEIVEKKGADSVPLIIGGASSNAAKILLSTLSPLSV
PLISYTASCPCLSDRRQYPTFFRTMPSDIYQAQAVAQLALRFNWTWIGAVVANNDYGLMAVK
VFQEETQGKGVCLAFVETLQRETIVSDARRAALTIQASTAKVILVFSWYTDVREVFYQLHKMN
VTDRQFLASEAWSTSEVLLKDPITSRVSSGVVGVAIASEHIPGFNGFLRGLNPSLRPWDQFL
QEFWEQEFGCSPSPPPNVTGHLKASLPPCSGAESLEERQHPFTDTSQLRVTYNVYLAVYAA
ANALHSLLSCPGHNSPPGSSNCTSSKSIKPTELLQHLSRVNFTTPQGELLYFQGADIPAKYD
LINWQRGADGTLKLVLIGRVAGFDLRLDGSAIEWSTRNNEVPVSVCSESCPPGTRKANRKG
EPLCCFDCIPCADGEISNTSGSLQCERCPPEFWSNDGRTACAPRQLDFLSFNETLGITLTTVA
VSGAVVTTAVFVVF
HYRHTPMVRANNSELSFLLLVSLKLCFLCSLVFI
RPSVWSCRFQQAAF
ISFVLCVSCLQVKTIVVLAAFRSARPGASALMKWFGPSQQRGSVCIFTFVQARVIICIIWLSLS
PPVPQADLDMPGLQVTLECAMASVVGFSLVLGYIGLLACTCLLLAFLARKLPDNFNEAKLITF
SMLIFCSVWVAFVPAYISSPGKYSVAVEIFAILASSYGLLFCIFAPKCFIILLRPEKNTKKHLMMR
>Tn_15_1_F
SPTWLLFLCVVGGHVGLNVVSTVLCSHWGQRSDRNLSVDGDVMIGGLFNLYYIPSAIQQEY
TQLHPYERCSSLDIESLKNMYAMVFTVEEINRDATLLPGVRLGYRIRDTCSRYPWALDGALSL
VTGGLSSCNMTASSTDSVGGKMAADEKVVPLLIGPASSTTGIMLSSILQTLSVPIISFLASCPC
LSDRTKFPTFFRTIPSDIYQARAMAQLAIRFHWTWIGAVVVNNDYGQLAIQMFQEEIRGKEICL

EFIETVYRETVMTDARRIALTVQAATTRVILIFCWYKDAKEILLELAKRNVTGRQFLASEAWSTS
EELLQELAISEVANGVLGVAIQSSTIPGFEHFLRSLNPIHRPNDVFLRDLWEMEFKCSPSSFS
NASLPPCSGTESLVEMKHPFTDTSQLRVAHNVYLAVYAAAYALHSLLFCPGQDSPPGKPNC
TSPHHIQPIDVLQHLNKVNFTTPRGETFYFQGSDLTTRYDLVNWQKTPRGPLKLALVGRVDG
FNLMLNESAIHWSTGLNQVPVSVCSESCPPGTRKANRKGEPLCCFDCIPCADGEISNTSGS
LQCERCPPEFWSNDGRTACAPRQLDFLSFNETLGITLTTVAVSGAVVTTAVFVVFLHYRHTPM
VRANNSELSFLLLVSLKLCFLCSLVFIGRPSVWSCRFQQAAFGISFVLCVSCLLVKTLVVLAVF
RSAQPGSRATKWFGPSQQRGSVCLFTSIQIIICTVWLSVSPPNPDRNLGFQGSKVTLECAMA
SVVGFSLVLGYIGLLACTCLLLAFLARKLPDNFNEAKLITFSMLIFCAVWVAFVPAYISSPGKYV
VAVEIFAILASSYGLLFCIFAPKCFIILLRPERNTKKDMMSRT
>Tn_V2R2_F
ARLFLLPLVCLWLLHLRVSADSTCKLKAKFNLSGYKSVEKKTVVVGGMFPVHMRVASSGRN
TSRVPVSSGCEGFNFRTFRWTRTMLFAIDEINRRSDLLPDTDLGYVIYDSCFTISKAVEGTLTY
LTGQDEAVPNYRCGSGPPLAALVGAGGTDLSIATARILGLYHFPQVSYCSTCSALESKFQFPT
FLRTVPSDQHQSSAIAKLVIGFGWTWVG
ISANDDYGKYGIKDFKEQVEEAGVCISFSETLPKGNSPEDIQRIVQTVVESTAKIIVVFSSDVDL
SPLVAELLRNNVTNRTWIASEAWVTSALLLEPGASSLLGGTLGFAVRRGSIPGLQRYLLDLDP
YGDPLTEEFWETVFNCTLDYGKALRQSGQGLCSGLESLAQLNNTYSDISQLRITYSVYKAVY
AVAHALHNLEHCQQGQGPFNGKDCADIGSFEPWQLMYYLKNVRYT
PHTGEEVFFNDGTVEAFYDLINWQFSSDGEISYVRVGHFNASAAPEEQMSISNSSILWNNDT
PEPPRSVCSDNCQPGTRKGIRQGEPVCCFDCIPCADGEISNTTNARECILCGEDDWSNQAH
DACVPKIIEFLAFGEPLGITLIVISACGAVVTIAVAVVFILNASTPLVKANDAVLSLLLLFSLVVTFL
CSIVFLGEPQDWSCMTSQVALALGFALCLSCIMTRSCRREIWRCFLIPQAVACTVWLVLFPP
HAVKNTSAQNIKIILECDEGSIVFICCIFAYDILLALIAFIFAFIARKLEDHFSEGKSMTFGMLVFFI
VWISFVPAYLSTRGKFMVAVQIFAILASSFGLLTCIFLPKCYILLIKPERNKEDMMRP
